# *N*-Alkylation and *N*-Methylation of Amines with Alcohols Catalyzed by Nitrile-Substituted NHC–Ir(III) and NHC–Ru(II) Complexes

**DOI:** 10.1021/acsomega.2c06341

**Published:** 2023-02-02

**Authors:** Sinem Çakır, Serdar Batıkan Kavukcu, Onur Şahin, Salih Günnaz, Hayati Türkmen

**Affiliations:** † Department of Chemistry, Faculty of Science, Ege University, Bornova, 35100 Izmir, Türkiye; ‡ Department of Occupat Health & Safety, Faculty of Health Sciences, Sinop University, Sinop 57000, Türkiye

## Abstract

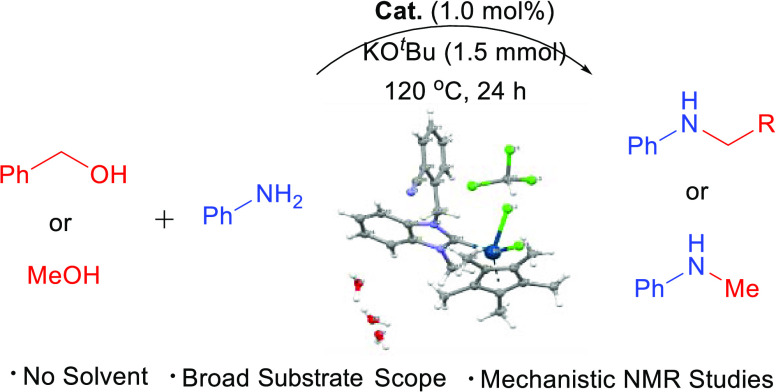

A series of nitrile-modified *N*-heterocyclic carbene (NHC) complexes of Ir(III) (**2a**–**e**) and Ru(II) (**3a**–**d**) have been prepared by transmetallation of [IrCp*Cl_2_]_2_ and [RuCl_2_(*p*-cymene)]_2_ forming an *in situ* NHC–Ag complex. The structures of all complexes were characterized by ^1^H NMR, ^13^C NMR, and Fourier transform infrared (FT-IR) spectroscopies. And the structures were clearly elucidated by performing X-ray diffraction studies on **2b**, **3a**, and **3c** single crystals. The complexes of NHC–Ir(III) (**2a**–**e**) and NHC–Ru(II) (**3a**–**d**) were investigated in the *N*-alkylation reaction of aniline derivatives with benzyl alcohols to form *N*-benzyl amines and in the *N*-methylation reaction of aniline derivatives with methanol. Both reactions were performed in solvent-free media. The Ir(III) complexes (**2a**–**e**) were found to perform essentially better than similar Ru(II) complexes (**3a**–**d**) in the *N*-alkylation and *N*-methylation reactions. Among the Ir(III) complexes (**2a**–**e**), the best results were obtained with **2b**. The catalytic mechanisms of both reactions were revealed by ^1^H NMR study. Formation of Ir-hydride species was observed for both reactions. This new report provides useful information to evaluate the activity of complexes and the differences in sensitivity between the NHCs.

## Introduction

In recent years, *N*-heterocyclic carbenes (NHCs) have been increasingly used as alternatives to phosphine ligands due to their easy preparation in organometallic chemistry for homogeneous catalyst, their enhanced stability resulting from strong metal–NHC bonds, and high tunability of steric and electronic properties.^1^ In addition, NHC ligands, unlike phosphine ligands, have good σ-donating properties and low toxicity, which is one of the main reasons why they are so preferred.^2^ It was determined that NHC complexes are versatile tools in homogeneous catalysis thanks to these properties. To date, NHCs have been involved in many reactions in the field of catalysis because they form stable complexes with many transition metals regardless of their oxidation state.^3^ Examples of these reactions are transfer hydrogenation,^4^ C–C coupling,^5^ olefin metathesis,^6^ hydrosilylation,^7^ and *N*-alkylation of amines.^8−10^

The transition-metal-catalyzed *N*-alkylation reaction was first performed by Grigg et al.^9^ and Watanable et al.^11^ Grigg’s rhodium and Watanable’s ruthenium catalysts performed the alkylation reaction of different amines with alcohols. To date, many catalytic reactions have been reported for the *N*-alkylation reaction. Many catalysts with different metal complexes including Ru, Ir, Fe, Co, Mn, Cu, Pd, Ni, and Cr have been synthesized for the *N*-alkylation of amines with alcohol.^12^ Especially, Ir and Ru complexes with NHC ligands have emerged as one of the most effective catalysts in the *N*-alkylation reactions.

The synthesis of target products from inexpensive, accessible, and nontoxic starting materials using transition-metal catalysts with a green chemistry approach is the focus of both inorganic and organic chemists.^13^ Amine-containing compounds are widely used in the manufacture of many materials as pharmaceutical, agrochemical, and synthetic intermediates.^14−16^ Therefore, the synthesis of C–N bond formation is of great interest to researchers. Many synthetic methods have been developed.^17,18^ Although most of the developed methods are realized with high efficiency, they produce high amounts of waste. In addition to these, since the materials used are highly toxic, they pose a significant problem for the environment.^19^ Therefore, the development of efficient and environmentally friendly catalytic strategies in the methods designed by researchers has become an important goal in synthesis chemistry. In recent years, methods based on borrowing hydrogen (BH) or hydrogen auto transfer (HA) methodology for transition-metal-catalyzed *N*-alkylation of amine with alcohol have attracted much attention due to their more sustainable and environmentally friendly nature.^20−23^ The use of alcohols as inexpensive and greener alkylating agents is one of the main reasons why the reaction is preferred. The BH methodology is also a powerful atom-economical catalytic strategy.^24^

Our previous results obtained by CN-modified Pd(II) catalysts in Suzuki–Miyaura cross-coupling reactions^25^ encouraged us to investigate the catalytic activities of Ir(III) and Ru(II) complexes of similar NHC ligands. The previous results prompted us to synthesize Ir(III) (**2a**–**e**) and Ru(II) (**3a**–**d**) complexes of similar NHC ligands and to explore the catalytic activities in the *N*-alkylation and *N*-methylation reactions. Our objective was to investigate the catalytic properties of Ir(III) and Ru(II) complexes bearing CN-modified NHC ligand in the different catalytic reactions. So, we reported NHC–Ir(III) (**2a**–**e**) and NHC–Ru(II) (**3a**–**d**) complexes of different azole skeletons (imidazole, benzimidazole, and benzothiazole) with CN substituent. The catalytic activity of complexes was investigated in the *N*-alkylation of aniline derivatives with benzyl alcohols. And we also report here that instead of reactive and toxic methyl halides that require their use in traditional *N*-methylation methods, it can be applied with good yields in direct *N*-methylation of amines with methanol by means of synthesized catalysts.

The strategies have been reported for both *N*-alkylation and *N*-methylation reactions with Ir and Ru catalysts. Huang et al. have reported the *N*-alkylation of amines with alcohols by the hetero-bidentate NHC-phosphine Ru catalyst with low catalyst loading (0.25 mol %).^8a^ Williams et al. developed an *in situ* catalytic system using [Ru(*p*-cymene)Cl_2_]_2_ with the bidentate phosphines dppf or DPEphos ligands.^8b^ The authors reported the preparation of some simple pharmaceutical drugs by means of *N*-alkylation reactions. Valerga et al. reported the use of Ru(II) arene complexes with picolyl-functionalized NHC ligands to the *N*-alkylation of amines with alcohol reaction.^8c^ There are many reported studies by Ozdemir et al. using NHC–Ru complexes including aromatic substituents for the *N*-alkylation reaction.^8d−8f^ Also, Ozdemir et al. reported acetal-functionalized NHC–Ru complexes to evaluate the *N*-alkylation of pyrrolidine and morpholine with benzyl alcohol.^8g^ Seayed et al. prepared NHC–Ru complexes with benzannulated ligands, and they used *N*-alkylation reaction for the synthesis of pharmaceutically important amines.^8h^ Bruneau et al. developed *in situ* catalytic conditions with benzimidazolium sulfanate salts and [RuCl_2_(*p*-cymene)]_2_ for the *N*-alkylation reaction.^8i^ Although studies of Ir(III) complexes are not as famous as Ru(II) complexes, some studies have been reported with NHC–Ir(III) complexes. Hou et al. produced an efficient and recyclable catalyst for *N*-alkylation reaction with silica-supported NHC–Ir complexes.^9a^ Royo et al. reported water-soluble NHC–Ir complexes bearing ester and amide groups for the *N*-alkylation reaction in water.^9b^

*N*-Alkylation of amines with methanol using NHC complexes is limited in the literature. Fujita et al. reported NHC–Ir(III)Cp* complexes as catalysts for the *N*-alkylation of primary amines with methanol.^10a^ They developed N,C(carbene)-chelated Ir(III) complexes with different electronic effects and steric hindrances.^10b^ Other researchers reported Ir-bis(NHC) catalysts for the transformation of amine monoalkylation.^10c^ These literature reports demonstrate the ability of iridium NHC complexes in *N*-alkylation reactions. Our study also provides good results that can compete with the literature reports and include in-depth mechanism studies in both reactions.

## Results and Discussion

### Synthesis of Azolium Salts, and Ir(III)–NHC and Ru(II)–NHC complexes

**Scheme 1:**
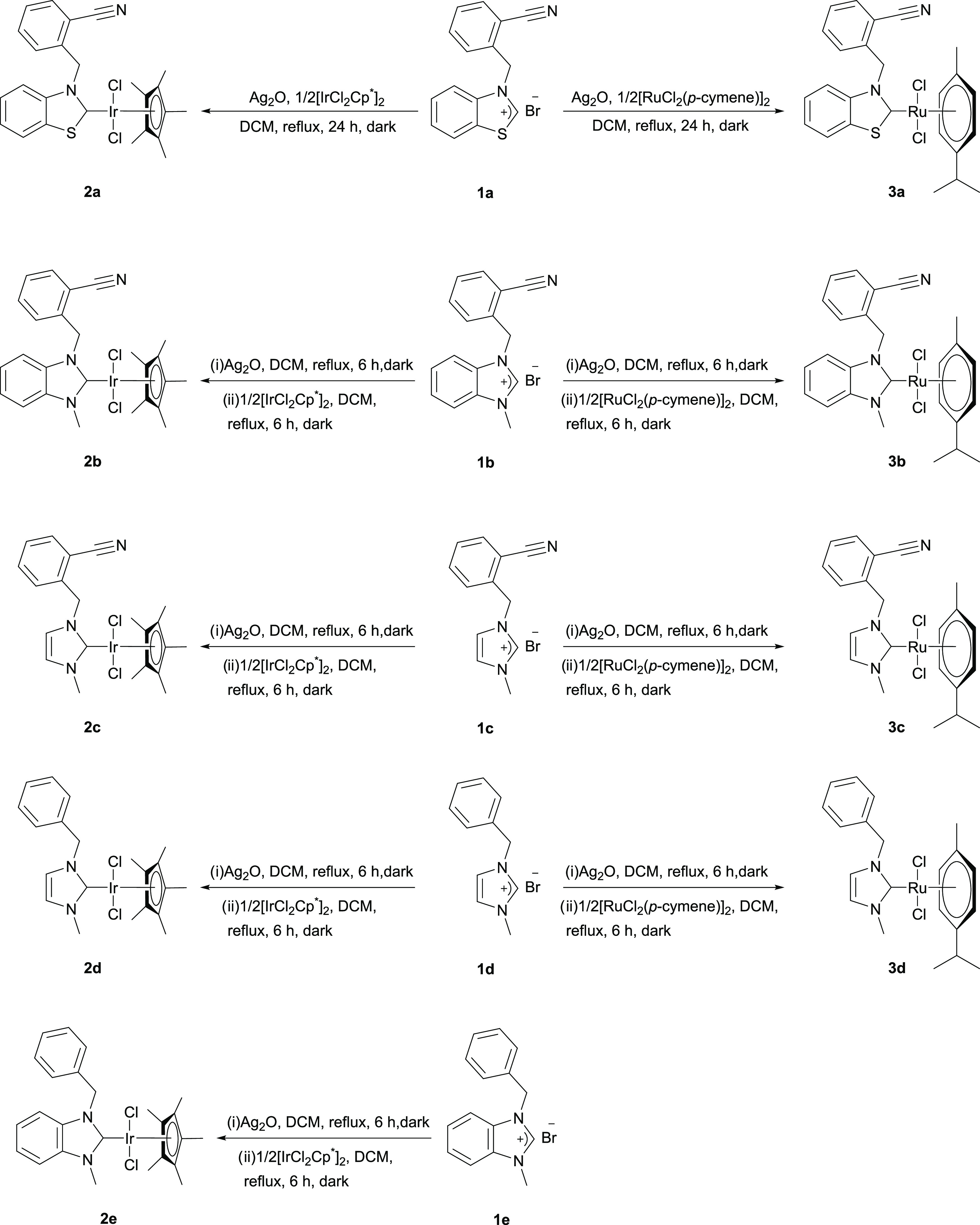
Synthesis of Ir(III)–/Ru(II)–NHC Complexes **2a**–**e** and **3a**–**d**

Azolium salts (**1a**–**d**) were synthesized in a one-pot reaction according to the previously developed procedure.^25^ Ligands were characterized by ^1^H-, ^13^C-NMR, and Fourier transform infrared (FT-IR) spectroscopic studies, and the spectroscopic data of the compounds obtained agree with the previous report. The characteristic signal of the −C≡N– group of azolium salts around 117 ppm confirmed the presence of −C≡N– in ^13^C NMR spectra. Also, the down-field signal at 10.75, 9.84, and 10.22 ppm proved the formation of benzothiazole, benzimidazole, and imidazole salts, respectively. [IrCl_2_(Cp*)(NHC)] and [RuCl_2_(*p*-cymene)(NHC)] complexes (**2a**–**e** and **3a**–**d**) were obtained in 75–86% yields by transmetalation from Ag–NHC derivatives using a two-step procedure (Scheme 1). The complexes (**2d**,**e** and **3d**) were synthesized to better understand the importance of the nitrile group. Column chromatography was used for the purification of the complexes. NHC iridium and ruthenium complexes (**2a**–**e** and **3a**–**d**) were isolated as orange-brown and air-stable solids with good yields. All of the resulting complexes were found to be well soluble in chlorinated solvents such as CH_2_Cl_2_ and CHCl_3_.

**Figure 1:**
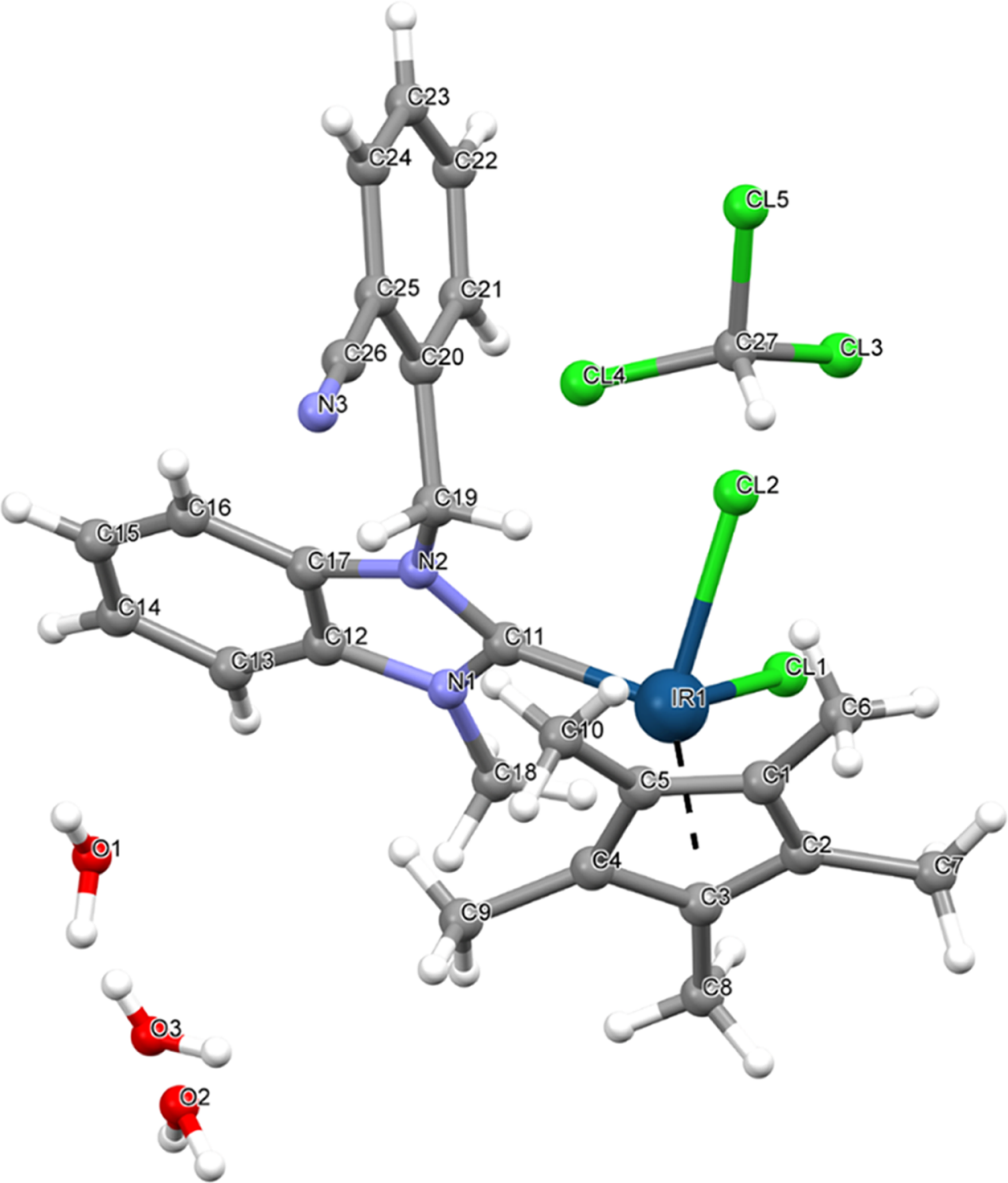
Molecular structure of **2b** showing the atom numbering scheme.

**Figure 2:**
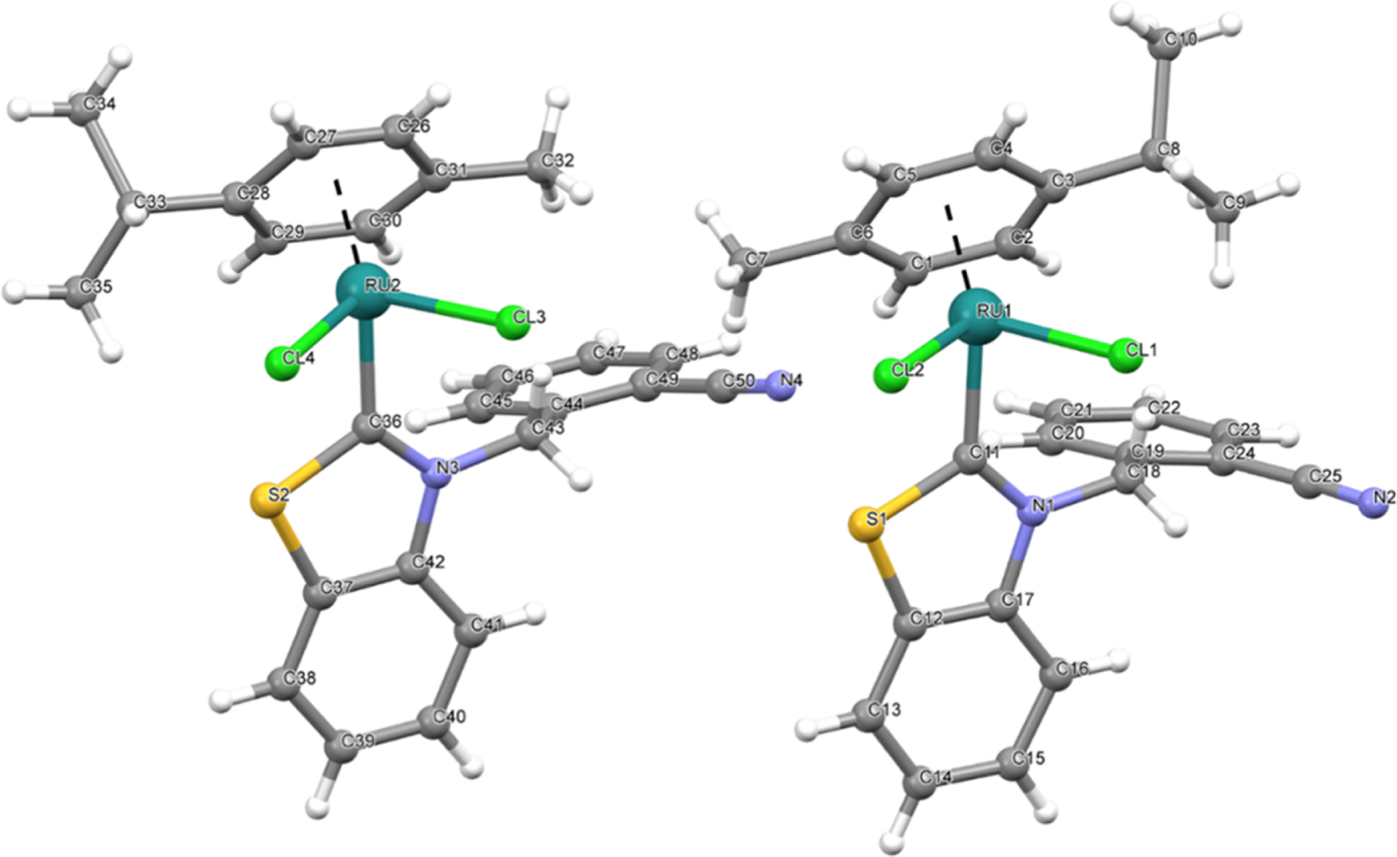
Molecular structure of **3a** showing the atom numbering scheme.

**Figure 3:**
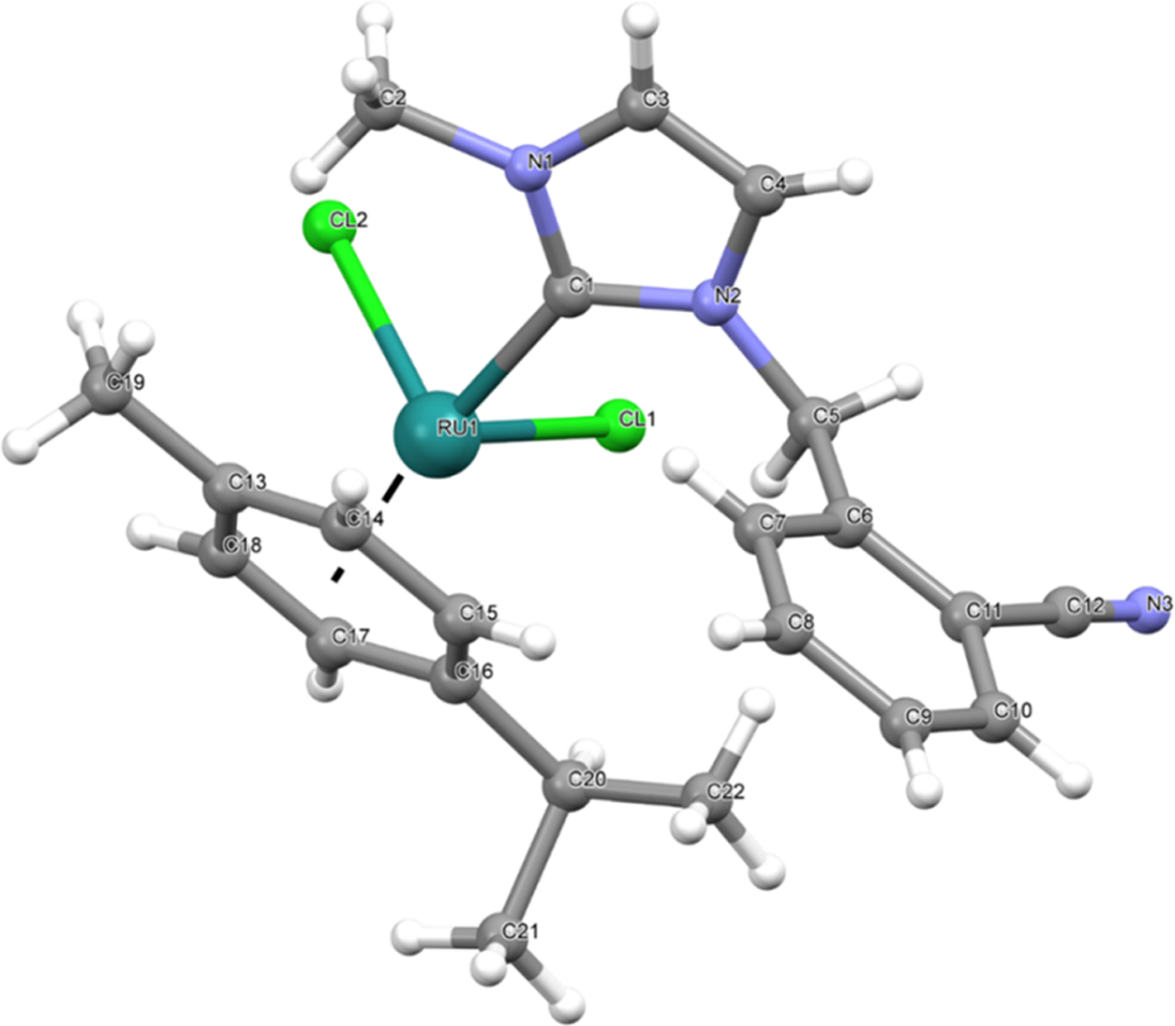
Molecular structure of **3c** showing the atom numbering scheme.

The examination of the structures of complexes (**2a**–**e** and **3a**–**d**) was provided by ^1^H and ^13^C NMR spectra. The absence of characteristic down-field signals of NC*H*N protons for the corresponding ligands (**1a**–**e**) in the ^1^H NMR spectra of the complexes (**2a**–**e** and **3a**–**d**) proved their formation. Meanwhile, Ir(III) (**2a**–**e**) and Ru(II) (**3a**–**d**) complexes showed characteristic Ru–Ccarbene and Ir–Ccarbene signals at δ = 203.3, 172.2, 157.9, 156.6, 170.9, 229.2, 191.5, 230.7, and 174.2 ppm, respectively. In the ^1^H NMR spectrum, the benzylic CH_2_ protons of **3a** showed a single peak, while the benzylic CH_2_ protons of **2b** and **3c** were not observed in the spectrum. This difference in **2b** and **3c** may be due to the intermolecular interaction of hydrogen. The structures of the complexes (**2b** and **3c**) were confirmed by X-ray diffraction studies (Figures 1–3) and the datas were represented in Table 2. The analytical data of the synthesized complexes (**2a–e** and **3a**–**d**) are summarized in Table 1. The FT-IR spectra of the complexes (**2a**–**e** and **3a**–**d**) were almost the same with similar structures (Figures S30–S38). The stretching vibrations of the nitrile functionality (C≡N) involved in the structure were demonstrated by observing a sharp band of medium intensity at 2219–2222 cm^–1^. The presence of the −C=N– group in the complexes was confirmed by the presence of ν(C=N) bands in the spectrum between 1656 and 1510 cm^–1^.

**Table 1:** Melting Points, Yields, and Selected ^13^C NMR Data of **2a**–**e** and **3a**–**d**

entry	complex	yield (%)	m.p. (°C)	*υ* (C≡N) (cm)^−1^	^13^C NMR
1	**2a**	80	290.3	2221	203.3
2	**2b**	75	275.1	2221	172.2
3	**2c**	78	281.1	2219	157.9
4	**2d**	61	239.4		156.6
5	**2e**	52	229.8		170.9
6	**3a**	85	243.0	2221	229.2
7	**3b**	83	248.4	2221	191.5
8	**3c**	86	230.4	2222	230.7
9	**3d**	57	211.3		174.3

**Table 2:** Selected Bond Distances (Å) and Angles (°) for Complexes **2b**, **3a**, and **3c**

complex **3a**			
C11–Ru1	2.059(10)	C36–Ru2	2.038(10)
Cl1–Ru1	2.417(3)	Cl2–Ru1	2.418(3)
Cl1–Ru1–Cl2	87.27(10)	C11–Ru1–Cl1	89.0(3)
Cl1–Ru1–Cl2	87.27(10)	C36–Ru2–Cl3	90.3(3)
C36–Ru2–Cl4	86.3(3)	Cl4–Ru2–Cl3	86.01(11)
complex **2b**			
C11–Ir1	2.050(15)	Cl1–Ir1	2.450(5)
Cl2–Ir1	2.424(5)		
C11–Ir1–Cl1	93.7(5)	Cl2–Ir1–Cl1	84.25(19)
complex **3c**			
C1–Ru1	2.082(3)	Cl1–Ru1	2.4350(11)
Cl2–Ru1	2.4287(10)		
C1–Ru1–Cl1	88.56(10)	C1–Ru1–Cl2	90.08(10)

### Catalytic *N*-Alkylation of Amines with Alcohols

*N*-Alkylation of amines with alcohols has become of interest because of its relevance to the atom-economic synthesis of pharmaceutically important molecules.^26^ Initially, in our study, *N*-alkylation reaction of aniline (**10a**) and benzyl alcohol (**11a**) was chosen as a model reaction to determine the catalytic potential of NHC–Ir(III) and NHC–Ru(II) complexes (**2a**–**e** and **3a**–**d**). The results of the relevant studies are listed in Table 3. The reaction was carried out in the presence of complex (**2a**–**e** and **3a**–**d**) as a catalyst and KO^*t*^Bu (1.5 mmol) in a solvent-free setting at 120 °C open to air for 24 h (Table 3, entries 1–6). In general, NHC–Ir(III) complexes (**2a**–**e**) exhibited higher catalytic activity than NHC–Ru(II) complexes (**3a**–**d**). Among the NHC–Ir(III) complexes, complex **2b** containing benzimidazole skeleton was the most effective catalyst and the product (**12aa**) was obtained in 80% yield after 20 h. A significant decrease in the catalytic activity was observed with the Ir(III) complex (**2a**), which has different heteroatoms in the structure of the catalyst. Here, due to the inability of the methyl group of the sulfur atom in the structure, the electronic and steric effects change and affect the charge density on the metal, leading to a decrease in the catalytic activity (Table 3, entry 1). Here again, the reason why **2b** was more active than **2c** is that it has a benzene ring fused in the 4,5-position of the imidazole ring in the structure. This was a result of electron density originating from the benzene ring (Table 3, entries 2 and 3). In the next step, the effect of base was investigated. Replacing KO^*t*^Bu with KOH, Cs_2_CO_3_, or K_2_CO_3_ did not positively affect yield. In fact, the use of K_2_CO_3_ significantly reduced the yield (Table 3, entries 7–9). When the catalyst loading was reduced from 1 to 0.5 mol %, an unsatisfactory effect on the yield was observed and the yield decreased to 48% (Table 3, entry 11). Thus, the optimum catalyst loading in this reaction was determined as 1.0 mol %. [IrCl_2_Cp*]_2_ did not catalyze the reaction (Table 3, entry 12). However, *in situ* experiment did not display good efficiency in the catalytic cycle (Table 3, entry 13). There was no yield in the absence of catalyst (Table 3, entry 14). Also, an air atmosphere was required for the reaction (Table 3, entry 15). As a result, the reaction conditions were determined (Table 3, entry 10). The catalytic results of **2d**, **2e**, and **3d** showed the importance of the nitrile group (Table 3, entries 16–18). To better understand the effect of the nitrile group, **2b** was heated in the presence of NaBF_4_. The newly formed cationic nitrile coordinated complex could not be isolated and it may be unstable. FT-IR analyses of the samples taken from the solution medium were performed at the beginning and end of the experiment. According to the FT-IR spectra, the changes in the fingerprint region and the newly formed metal–nitrogen bond around 630 cm^–1^ support the formation of the cationic complex (Figures S43 and S44). This shows that the nitrile can coordinate to the metal in the catalytic process. Literature reports indicate that the presence of the nitrile group in the side chain provides stability to the complex.^41^

**Table 3:**
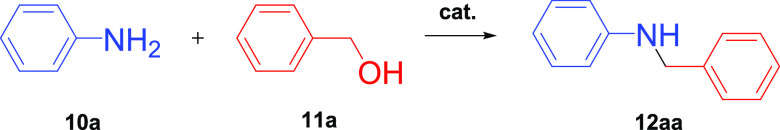
Optimization of Reaction Conditions for *N*-Alkylation of Aniline with Benzyl Alcohol^a^

entry	cat.	base	yield (%)
1	**2a**	^*t*^BuOK	55
2	**2b**	^*t*^BuOK	80
3	**2c**	^*t*^BuOK	72
4	**3a**	^*t*^BuOK	40
5	**3b**	^*t*^BuOK	67
6	**3c**	^*t*^BuOK	67
7	**2b**	Cs_2_CO_3_	62
8	**2b**	K_2_CO_3_	33
9	**2b**	KOH	58
10^b^	**2b**	^*t*^BuOK	93
11^c^	**2b**	^*t*^BuOK	48
12^d^	[IrCl_2_Cp*]_2_	^*t*^BuOK	22
13^e^	**1b**/[IrCl_2_Cp*]_2_	^*t*^BuOK	42
14		^*t*^BuOK	trace
15^f^	**2b**	^*t*^BuOK	69
16	**2d**	^*t*^BuOK	62
17	**3d**	^*t*^BuOK	58
18	**2e**	^*t*^BuOK	64

a Reaction conditions: Aniline (1.0 mmol), benzyl alcohol (1.5 mmol), ^*t*^BuOK (1.5 mmol), cat. (1.0% mol), 20 h, 120 °C, air atmosphere.

b 24 h.

c (0.5% mol).

d Only [IrCl_2_Cp*]_2_ complex was used as the catalyst.

e *In situ* generated catalytic system with **1b** carbene precursor and [IrCl_2_Cp*]_2_ was used.

f Under argon atmosphere. Yields were determined using ^1^H NMR spectroscopy.

Many different methods can be applied in the synthesis of secondary amines. However, conventional methods use environmentally harmful organic solvents and alkyl halides or stoichiometric amounts of reducing agents.^27−29^ In recent studies, the use of cheap and low-toxic alcohols instead of environmentally harmful organic solvents has attracted a lot of attention. *N*-Alkylation of amines with alcohols is a green method in the synthesis of substituted amines, which are of great importance in synthetic applications. Ru- and Ir-catalyzed amine alkylation has previously attracted the attention of several groups and has been reported.^8,9^ The extent of Ir-catalyzed *N*-alkylation of amines with benzyl alcohols under optimal reaction conditions was investigated (Table 4). A wide variety of substrates bearing electron-donating or electron-withdrawing substituents on the aryl ring of aniline were successfully completed with benzyl alcohol in high yields at 120 °C (Table 4). *N*-Alkylation of 4-methylaniline and 2-methylaniline with benzyl alcohol was investigated using **2b** as the catalyst to obtain *N*-benzyl-4-methylaniline (**12b**) and *N*-benzyl-2-methylaniline (**12c**) with 72 and 65% yields, respectively. For the di-alkylated substituent, such as 2,4-dimethylaniline, *N*-benzyl-2,4-dimethylaniline (**12d**) was obtained with relatively low conversion compared to mono-alkylated substrate. Moreover, 64% catalytic yield was achieved by 2-nitroaniline and benzyl alcohol, resulting in product *N*-benzyl-2-nitroaniline (**12e**). It was observed that the methoxy substituent in the *para*-position of aniline had a better formation rate of the corresponding product than in the *ortho*-position substituent. And the desired products *N*-benzyl-4-methoxyaniline (**12f**) and *N*-benzyl-2-methoxyaniline (**12g**) were converted in 86 and 71% yields, respectively. The products of halogen-substituted anilines, *N*-benzyl-4-bromoaniline (**12h**) and *N*-benzyl-4-chloroaniline (**12i**), were obtained in yields of 82 and 80%, respectively. In addition, aliphatic amine such as *N*-hexylamine was converted to the desired product, *N*-benzylhexan-1-amine (**12j**), in a yield of 45%. Aniline derivatives containing sterically hindered heterocyclics also resulted in good yields (89 and 79%) in *N*-alkylation reactions with the products of *N*-benzylnaphthalen-2-amine (**12k**) and *N*-benzyl-[1,1′-biphenyl]-4-amine (**12l**).

**Table 4:**
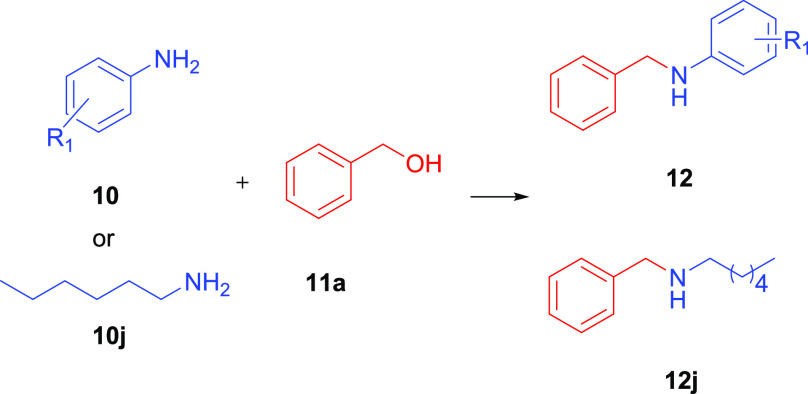

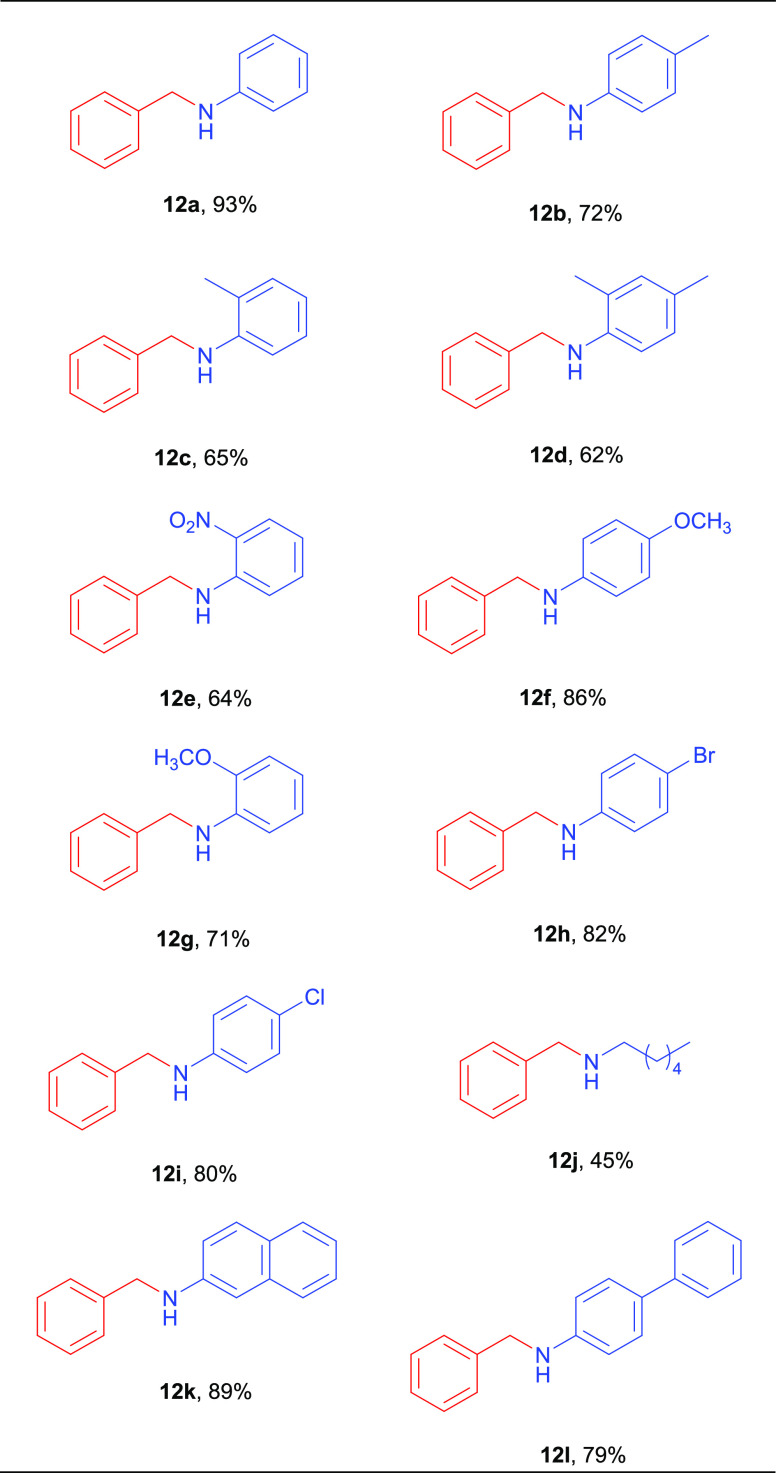
*N*-Alkylation of Amines Using Benzyl Alcohol^a^

a Reaction conditions: **10** (1.0 mmol), **11a** (1.5 mmol), KO^*t*^Bu (1.5 mmol), **2b** (1.0% mol), 24 h, 120 °C. Isolated yields.

To determine the broader applicability of catalytic conversions, we investigated the *N*-alkylation of aniline with benzyl alcohol derivatives (Table 5). As shown in Table 5, the reaction of both electron-rich and electron-deficient benzyl alcohols was investigated, and the reactions proceeded smoothly. The desired products were obtained in 60–93% yields. First, benzyl alcohol-containing electron-donating group at the *para*-position, namely, CH_3_ was tested, and good yields were obtained with *N*-(4-methylbenzyl)aniline (**13a**). The reaction of aniline with benzyl alcohol derivatives bearing 4-bromo and 2-bromo yielded corresponding products *N*-(4-bromobenzyl)aniline (**13b**) and *N*-(2-bromobenzyl)aniline (**13c**) in 73–65% yields, respectively. When using alcohols consisting of 4-Cl, 4-OCH_3_, and 2,4,6-CH_3_ groups, the corresponding products *N*-(4-chlorobenzyl)aniline (**13d**), *N*-(4-methoxybenzyl)aniline (**13e**), and *N*-(2,4,6-trimethylbenzyl)aniline (**13f**) were obtained in good yields of 81, 93, and 60%, respectively.

**Table 5:**
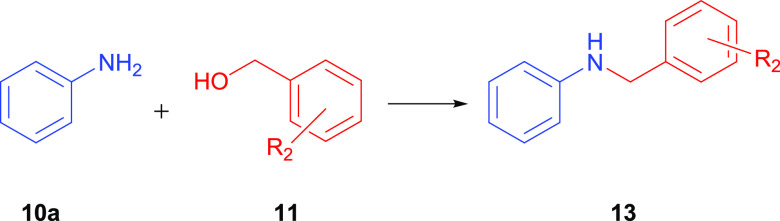

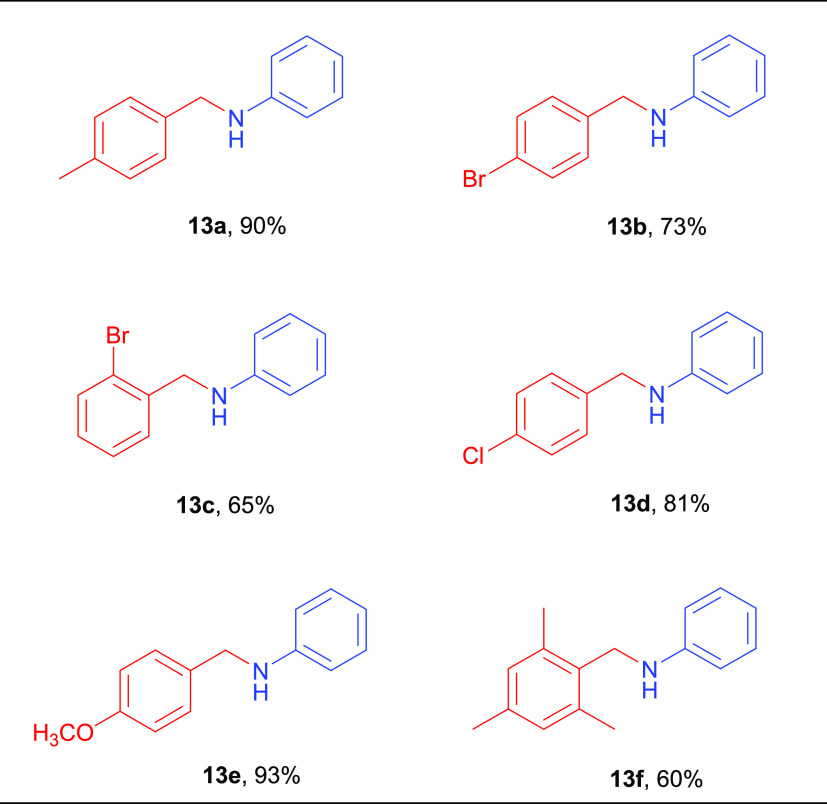
*N*-Alkylation of Aniline Using Benzyl Alcohol Derivatives^a^

a Reaction conditions: **10a** (1.0 mmol), **11** (1.5 mmol), KO^*t*^Bu (1.5 mmol), **2b** (1.0% mol), 24 h, 120 °C. Isolated yields.

**Scheme 2:**
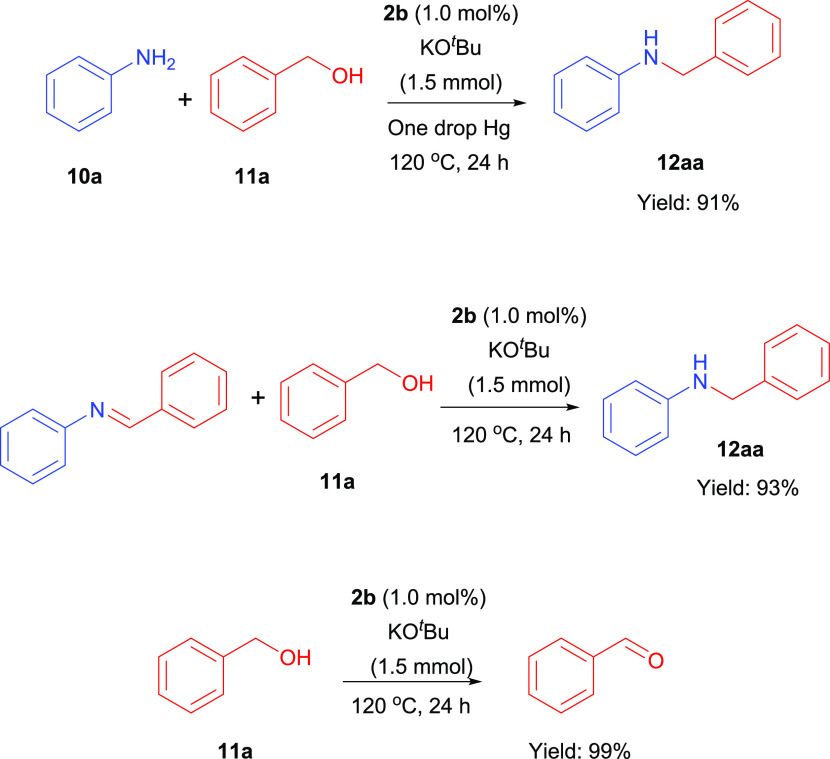
Mechanism and Control Experiments for *N*-Alkylation of Aniline with Benzyl Alcohol

The control experiments were conducted to understand the progress of the reaction (Scheme 2). The mercury test was performed for precatalyst **2b** using one drop of Hg. Observation of 91% efficiency supported homogeneous catalysis. The formation of amine from the imine was controlled, and the amine was successfully formed in 93% yield. The catalyst (**2b**) was proven to successfully convert benzyl alcohol to benzaldehyde.

**Figure 4:**
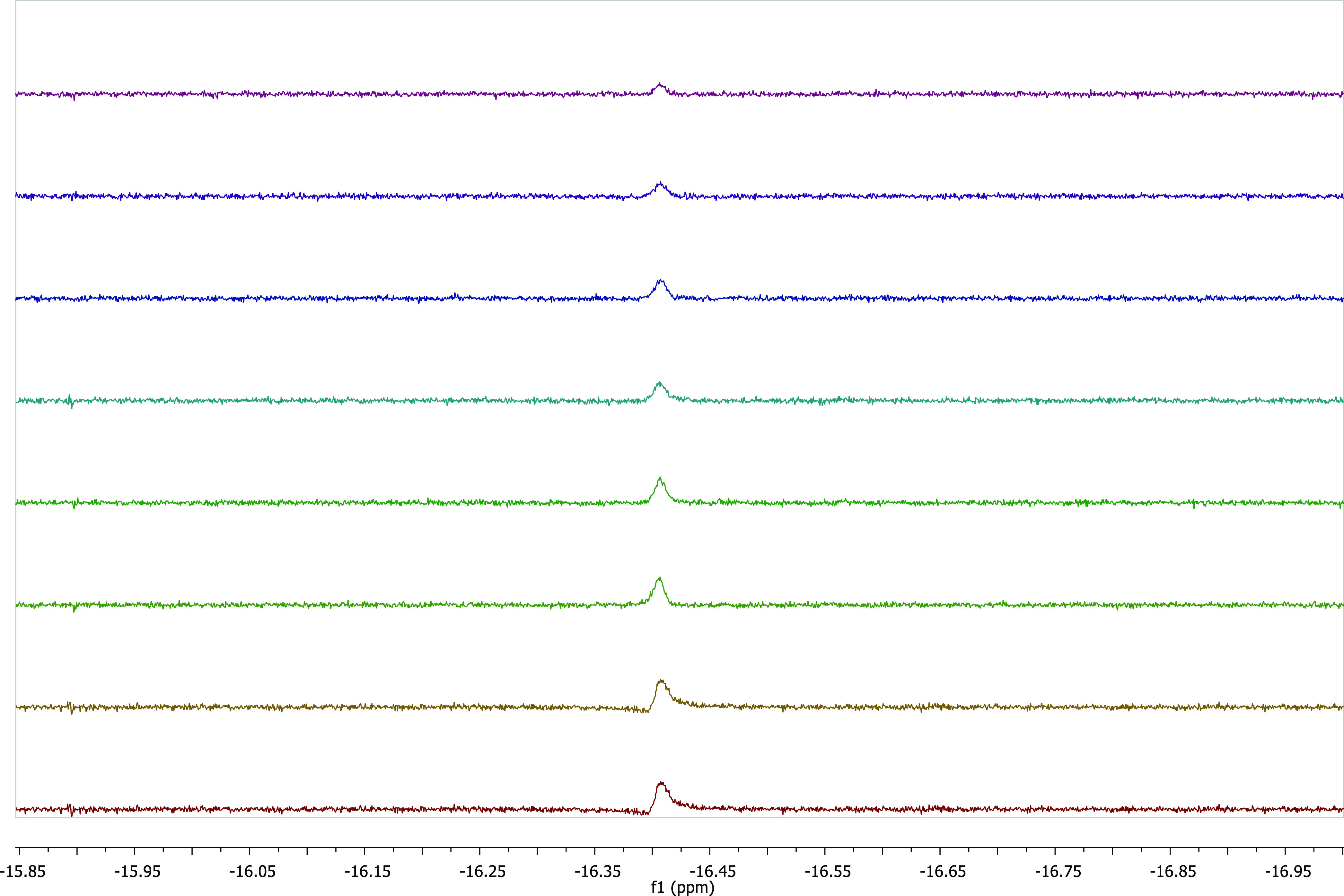
^1^H NMR monitoring of the hydride signal of Ir-hydride (located at −16.40 ppm and zoomed in): aniline (0.1 mmol), benzyl alcohol (0.15 mmol), KO^*t*^Bu (0.15 mmol), **2b** (5 mmol %), CD_3_OD (1 mL), 120 °C.

**Scheme 3:**
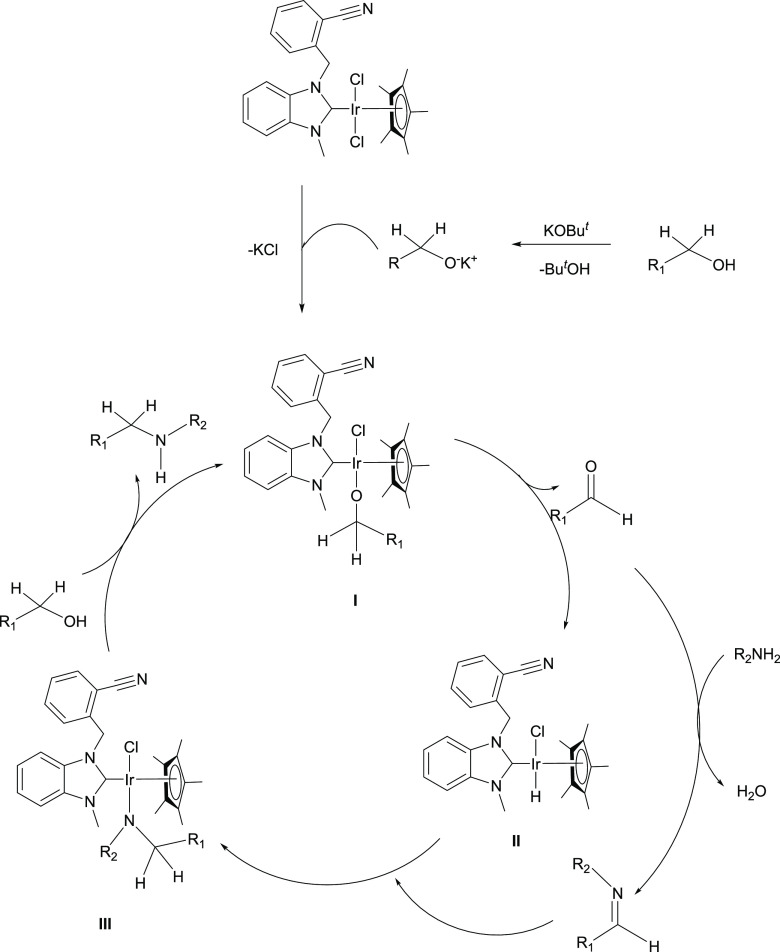
Proposed Reaction Pathway for the Iridium(III)–NHC-Catalyzed *N*-Alkylation

We performed NMR experiment to investigate the mechanism of *N*-alkylation of aniline with benzyl alcohol (Figure 4). The NMR experiment was carried out in CD_3_OD (1 mL) at 120 °C for 24 h in the presence of **2b** as the catalyst and KO^*t*^Bu (0.15 mmol). The possible mechanism of the *N*-alkylation of amines with primary alcohols is presented in Scheme 3. The mechanism starts with the leaving of proton of benzyl alcohol with help of KO^*t*^Bu. One of the metal-coordinated chlorine anions is separated to form KCl precipitate. Deprotonated alkoxy derivative coordinates to the metal from the oxygen atom (I). It loses one of its benzyl protons and leaves as an aldehyde. This step is essential for the formation of metal hydride species (II). The imine species is formed by the reaction of aldehyde and amine. The imine coordinates to the metal center and is reduced to amine (III). Imine formation and imine reduction to amine are rate-determining steps for this reaction. Imine and Ir-H formation were observed as soon as the reaction started and persisted throughout the reaction. A main hydride peak appeared in the hydridic region of the ^1^H NMR spectrum (δ = −16.408 ppm), which suggested that the catalytically active species were related to Ir-H formation (Figure 4). The imine peak was observed at 8.54 ppm in the low area of the ^1^H NMR spectra (Figure S39). Since the imine was formed as soon as the reaction starts, no aldehyde peak was observed. All ^1^H NMR spectra of the reaction mechanisms are presented in the Supporting Information. The reaction mechanism is completely dependent on the formation of imine. Under our reaction conditions, double-alkylated products were not observed.

### *N*-Methylation of Anilines with Methanol

*N*-Methylamines are widely used as basic intermediates and building blocks for the synthesis of many bulk and fine chemicals. The direct *N*-methylation of amines has attracted the attention of many researchers due to its significant selectivity and increased overall product yields, without using reactive and toxic methyl halides as methylating agents^30^ used in conventional *N*-methylation methods.^31,32^ In our study, *N*-methylation of amines with methanol was also reported. As shown in Table 6, *N*-methylation of anilines with methanol was successfully performed at 120 °C in the presence of 1.0 mol % **2b** and 1.5 mmol of base. The products were obtained in a yield of above 80%. The reaction of methanol with 4-methyl-, 2-methyl-, 2,4-methyl-, and 2,4,6-methyl-bearing aniline derivatives gave products 4-methyl-*N*-methylaniline (**14b**), 2-methyl-*N*- methylaniline (**14c**), 2,4-methyl-*N*-methylaniline (**14d**), and 2,4,6-methyl-*N*-methylaniline (**14e**), respectively, with yields in the range of 68–80%. In addition, *N*-methylation of 2-NO_2_-, 2-OCH_3_-5-CH_3_-, 4-OCH_3_-, 2-OCH_3_-, 4-Br-, and 4-Cl-substituted anilines gave the desired products 2-nitro-*N*-methylaniline (**14f**), 2-methoxy-5-methyl-*N*-methylaniline (**14g**), 4-methoxy-*N*-methylaniline (**14h**), 2-methoxy-*N*-methylaniline (**14i**), 4-bromo-*N*-methylaniline (**14j**), and 4-chloro-*N*-methylaniline (**14k**), respectively, in 60–91% yields. Anilines composed of different substituents, 4-Br-2,6-CH_3_, 2-CF_3_, naphthalene, and biphenyl, gave products 4-bromo-2,6-methyl-*N*-methylaniline (**14l**), 2-trifluoromethyl-*N*-methylaniline (**14m**), *N*-methylnaphthalen-2-amine (**14n**), and *N*-methyl-[1,1′-biphenyl]-4-amine (**14o**) in good yields of 72, 59, 88, and 85%, respectively. Although we increased the amount of methanol, double *N*-methylation did not occur, which led to a mixture of dimethylated amines. However, we performed the reaction with aliphatic amine, which is generally more nucleophilic than aromatic amines, such as *N*-octadecylamine. In our study, attempts for *N*-methylation of *N*-octadecylamine did not result in positive results and only trace yields were achieved.

**Table 6:**
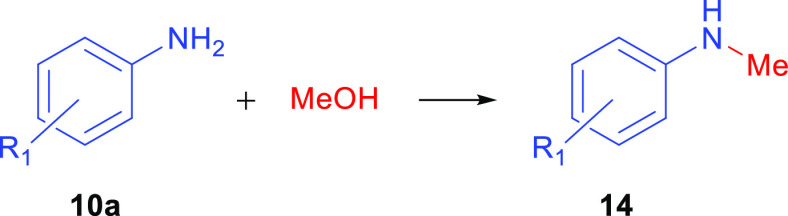

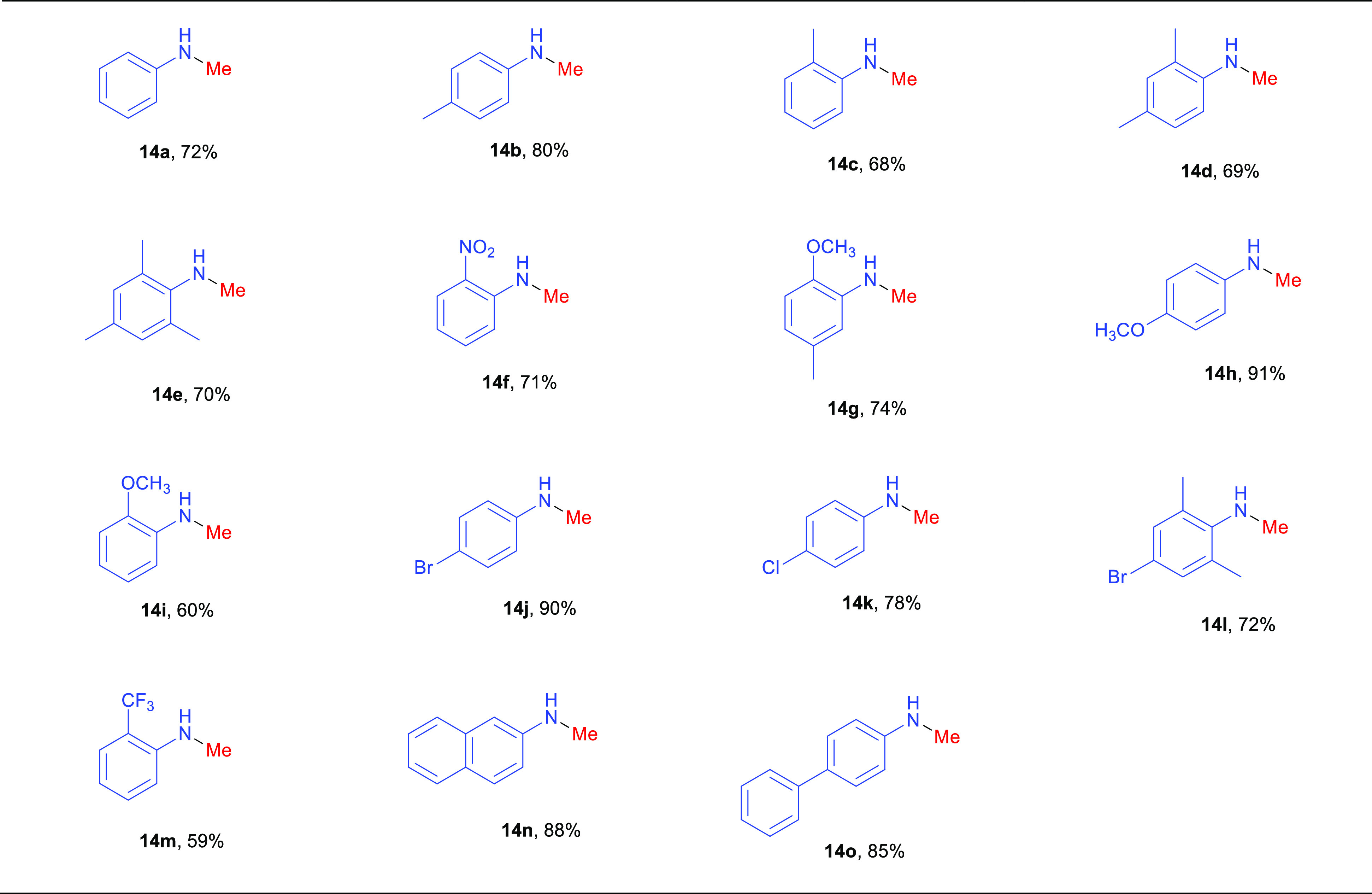
*N*-Alkylation of Amines with Methanol^a^

a Reaction conditions: **10a** (1.0 mmol), MeOH (0.4 mL), KO^*t*^Bu (1.5 mmol), **2b** (1.0% mol), 24 h, 120 °C. Isolated yields.

**Figure 5:**
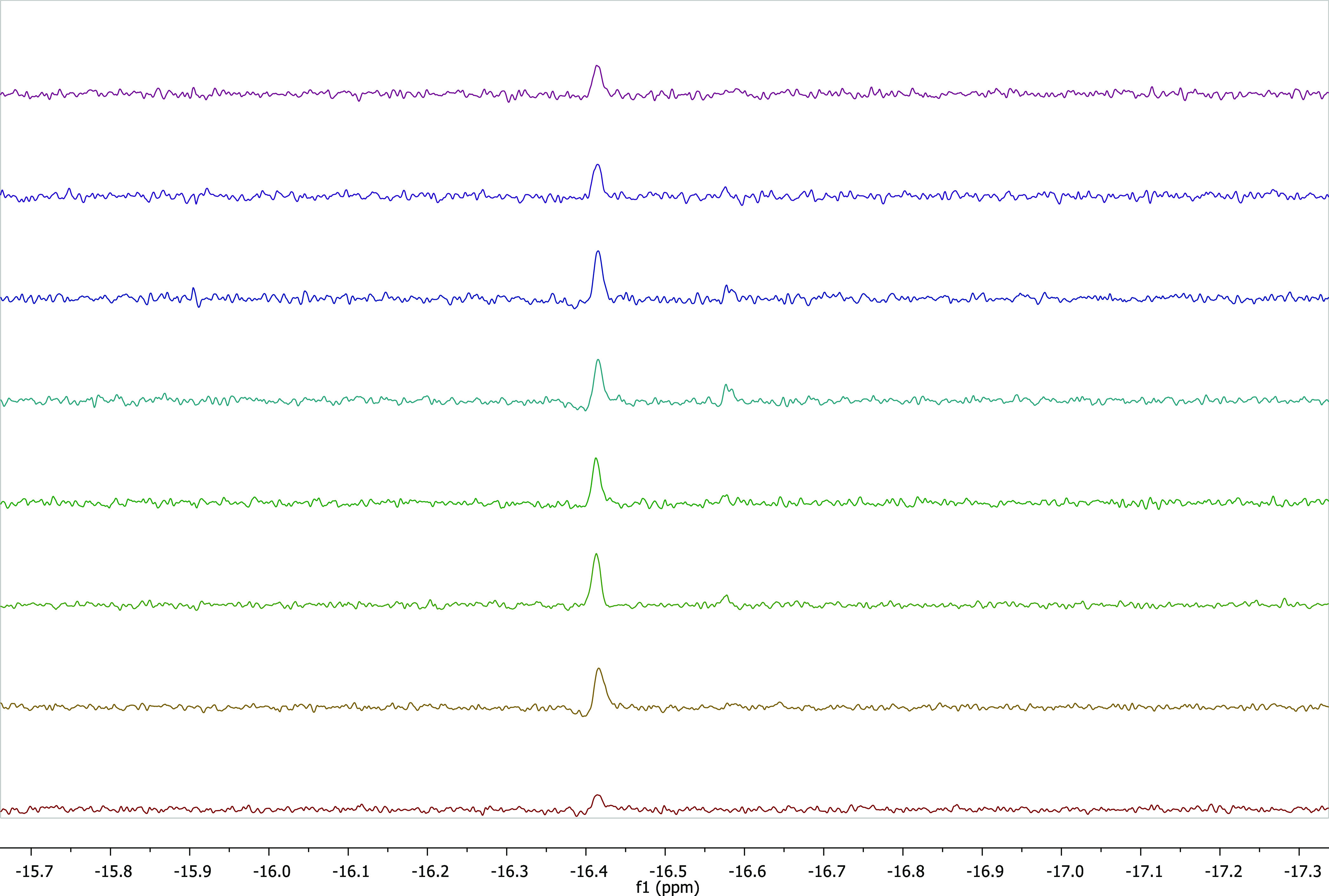
^1^H NMR monitoring of the hydride signal of Ir-hydride (located at −16.41 ppm and zoomed in): aniline (0.1 mmol), KO^*t*^Bu (0.15 mmol), **2b** (5 mmol %), CD_3_OD (1 mL), 120 °C.

**Scheme 4:**
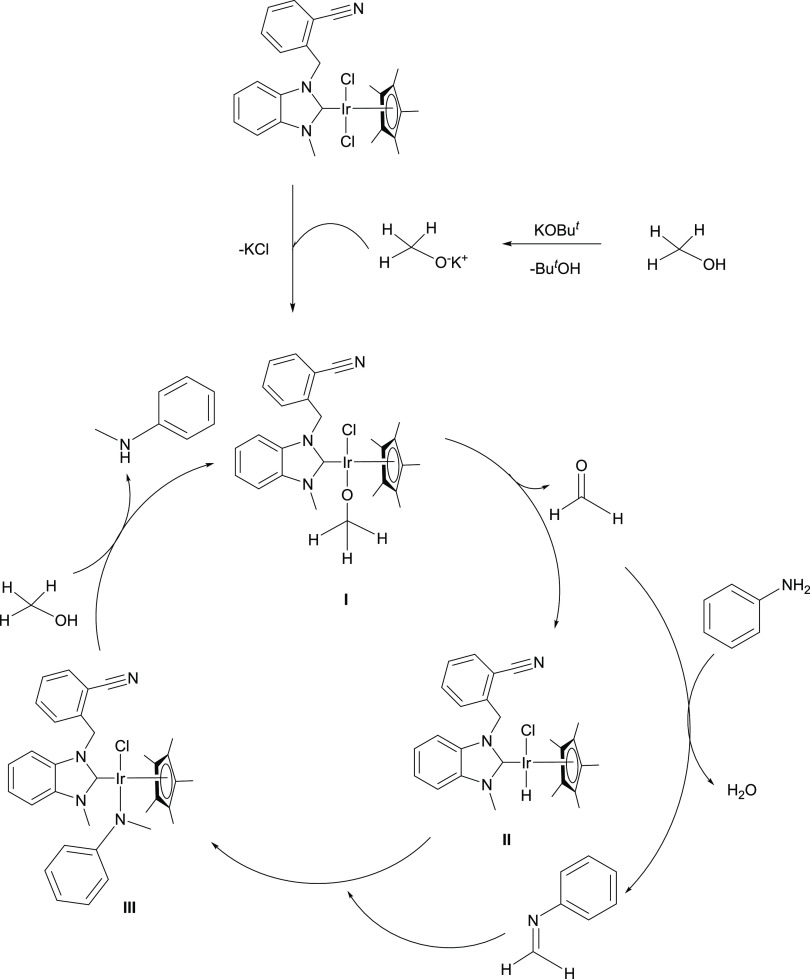
Proposed Reaction Pathway for the Iridium(III)–NHC-Catalyzed *N*-Methylation

We also performed NMR experiment to investigate the mechanism of *N*-methylation of aniline with methanol. The NMR experiment was carried out in excess CD_3_OD (1 mL) at 120 °C for 24 h in the presence of **2b** as the catalyst and KO^*t*^Bu (0.15 mmol) (Figure 5). The possible mechanism of the *N*-alkylation of amines with methanol is presented in Scheme 4. The mechanism is quite like the *N*-alkylation of amines with primary alcohols. The mechanism starts with the leaving of a proton of methanol with help of KO^*t*^Bu. One of the metal-coordinated chlorine anions is separated to form KCl precipitate. Deprotonated methanol derivative coordinates to the metal from the oxygen atom (I). It loses one of its protons and leaves as a formaldehyde. This step is essential for the formation of metal hydride species (II). There is a high probability of double proton bonding to the metal center by dissociating the other chloride ion. Other unstable peaks seen in the hydride region of the ^1^H NMR spectra support this. The aldehyde peak was observed in the first ^1^H NMR spectrum as soon as the reaction was prepared (Figure S40). But from the second spectrum, it quickly disappeared, and the imine was formed. The imine species is formed by the reaction of formaldehyde and amine. The imine coordinates to the metal center and is reduced to amine (III). Imine formation and imine reduction to amine are rate-determining steps for this reaction. Ir-H formation was observed as soon as the reaction started and persisted throughout the reaction. A main hydride peak appeared in the hydridic region of the ^1^H NMR spectrum (δ = −16.413 ppm), which suggested that the catalytically active species were related to Ir-H formation (Figure 5). The imine peak was observed at 8.15 ppm in the low area of the ^1^H NMR spectra (Figure S40).

**Scheme 5:**
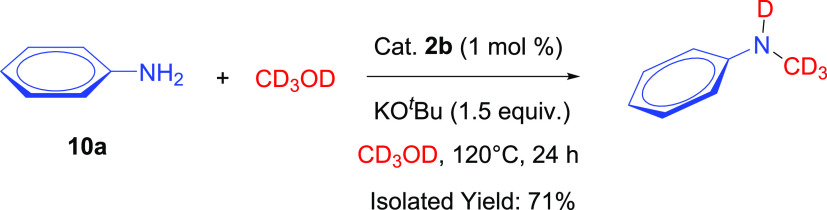
*N*-Methylation of Aniline with Methanol-*d*_4_

*N*-Methylation of aniline was performed using deuterated methanol to investigate the further mechanism (Scheme 5). Deuterated *N*-methylaniline was obtained with 71% isolated yield. ^1^H NMR spectra of deuterated and protonated *N*-methylaniline support the successful formation and isolation of deuterated *N*-methylaniline (Figures S41 and S42).^35^

## Conclusions

In conclusion, a series of NHC–Ir(III) (**2a**–**e**) and NHC–Ru(II) (**3a**–**d**) complexes bearing nitrile moiety were prepared. The complexes (**2a**–**e** and **3a**–**d**) were fully characterized *via*^1^H, ^13^C NMR and FT-IR spectroscopies. The crystal structures confirmed that complexes **2b**, **3a**, and **3c** exhibited piano-stool geometry. The complexes (**2a**–**e** and **3a**–**d**) were evaluated as catalysts in the *N*-alkylation of amines with primary alcohols and *N*-methylation of aniline with methanol. High conversions were obtained under mild conditions in both reactions. Mechanistic studies of both reactions were performed by NMR and their catalytic cycles were established. The Ir(III) complex (**2b**) was the most effective catalyst in both reactions. For both reactions, the mechanism proceeds through the metal hydride mechanism. The process of imine formation and reduction of the imine to the amine determines the rate of these reactions. The Ir-hydride species of **2b** were detected at −16.408 and −16.413 ppm.

## Experimental Section

### X-ray Crystallography

Suitable crystals of **2b**, **3a**, and **3c** were selected for data collection, which was performed on a D8-QUEST diffractometer equipped with a graphite-monochromatic Mo Kα radiation. The structure was solved by direct methods using SHELXS-201336 and refined by full-matrix least-squares methods on F2 using SHELXL-2013.^36,37^ All nonhydrogen atoms were refined with anisotropic parameters. The water H atoms were refined freely. The other H atoms were located from different maps and then treated as riding atoms with C–H distances of 0.93–0.97 Å. The following procedures were implemented in our analysis: data collection: Bruker APEX2;^38^ program used for molecular graphics: MERCURY program;^39^ software used to prepare material for publication: WinGX.^40^ Details of data collection and crystal structure determinations are given in Table 2.

### Materials

Unless otherwise noted, all operations were performed without taking precautions to exclude air and moisture. The glass equipment was heated under vacuum to remove oxygen and moisture, and then they were filled with argon. Starting compounds and reagents were obtained from Merck, Fluka, Alfa Aesar, and Acros Organics; Ruthenium(III) chloride hydrate, iridium(III) chloride hydrate, and α-terpinene were obtained from Alfa Aesar, and dichloromethane, diethyl ether, and toluene were obtained from Merck and Ridel de Haen. [IrCl_2_Cp*]_2_ was synthesized according to the published procedures by a reaction of iridium(III) chloride and pentamethylcyclopentadiene.^33^ [RuCl_2_(*p*-cymene)]_2_ was prepared according to the method reported by Bennett and Smith through the reaction of ruthenium(III) chloride with α-terpinene.^34^ Catalytic reactions were carried out under the inert atmosphere on Carousel 12 Plus Reaction Station system. ^1^H and ^13^C NMR spectra were recorded on a Varian AS 400 Mercury instrument. Melting points were measured on Gallenkamp electrothermal melting point apparatus without correction. FT-IR spectra were recorded on a PerkinElmer Spectrum 100 series.

#### Synthesis of Compound **1e**

1-Methylbenzimidazole (250 mg, 1.89 mmol) was dissolved in 5 mL of toluene, and benzyl bromide (324 mg, 1.89 mmol) was added to boil under reflux for 24 h. The precipitate was then filtered off, washed with diethyl ether, and dried under vacuum to yield. Yield = 82%, 470 mg. ^1^H NMR (400 MHz, CDCl_3_): δ 11.15 (s, 1 H, NC*H*N), 7.69 (d, *J* = 8.4 Hz, 1 H, Ar–*H*), 7.59 (d, *J* = 8.4 Hz, 1 H, Ar–*H*), 7.49 (t, *J* = 8.4 Hz,1 H, Ar–*H*), 7.43 (m, 3 H, Ar–*H*), 7.19 (m, 3 H, Ar–*H*), 5.78 (s, 2H, N–C*H*_2_), 4.16 (s, 3H, N–C*H*_3_). ^13^C NMR (100 MHz, CDCl_3_): δ 142.6, 132.7, 132.0, 130.8, 129.3, 129.2, 129.0, 128.4, 128.3, 127.3, 113.8, 113.1, 53.5, 51.2, 34,1. IR, *ν*_max_ (cm^–1^) (CH_2_Cl_2_): 3434, 3141, 3033, 2055, 1811, 1705, 1612, 1569, 1490, 1456, 1427, 1358, 1349, 1274, 1265, 1209, 1195, 1166, 1132, 1093, 1020, 855, 821, 791, 750, 705, 660, 605, 569, 560, 532, 467, 423.

#### Synthesis of Complex **2a**

In a balloon, **1a** (250 mg, 0.75 mmol) under argon gas was suspended in dichloromethane. Ag_2_O (175 mg, 0.75 mmol) and [IrCl_2_Cp*]_2_ (301 mg, 0.38 mmol) were added over and stirred at 39 °C for 24 h protected from light. The remaining solid residue was washed with diethyl ether and then dried under vacuum. Yield = 80%, 400 mg. Elemental analyses for C_25_H_26_Cl_2_IrN_2_S (648.67): C, 44.9310; H, 4.2528; N, 3.9534; S, 2.7159. ^1^H NMR (400 MHz, CDCl_3_): δ 7.79 (d, *J* = 8.0 Hz, 1 H, Ar–*H*), 7.70 (d, *J* = 7.2 Hz, 1 H, Ar–*H*), 7.39 (d, *J* = 7.6 Hz, 1 H, Ar–*H*), 7.35 (m, 3 H, Ar–*H*), 7.30 (m, 1 H, Ar–*H*), 7.12 (t, *J* = 8.0 Hz, 2 H, N–C*H*_2_), 1.68 (s, 15 H, C_5_(C*H*_3_)_5_). ^13^C NMR (100 MHz, CDCl_3_): δ 203.3, 143.6, 139.1, 136.6, 133.4, 132.3, 128.8, 128.3, 126.6, 125.3, 121.9, 117.3, 114.5, 109.9, 91.1, 55.3, 8.6. IR, *ν*_max_ (cm^–1^) (CH_2_Cl_2_): 3418, 3060, 2965, 2910, 2221, 1599, 1510, 1483, 1454, 1379, 1357, 1314, 1284, 1270, 1207, 1141, 1116, 1053, 1029, 982, 913, 793, 762, 718, 674, 604, 552, 450, 432.

#### Synthesis of Complex **2b**

In a balloon, **1b** (250 mg, 0.76 mmol) and Ag_2_O (177 mg, 0.76 mmol) under argon gas were suspended in dichloromethane (5 mL) and stirred at 39 °C for 6 h protected from light. Then, [IrCl_2_Cp*]_2_ (303 mg, 0.38 mmol) was added and refluxed for 6 h. The remaining solid residue was washed with diethyl ether and then dried under vacuum. Yield = 75%, 380 mg. Elemental analyses for C_26_H_28_Cl_2_IrN_3_ (645,13): C, 46.4660; H, 4.8401; N, 5.8835. ^1^H NMR (400 MHz, CDCl_3_): δ 7.69 (d, *J* = 7.2 Hz, 1 H, Ar–*H*), 7.41 (d, *J* = 8.0 Hz, 1 H, Ar–*H*), 7.38 (d, *J* = 6.4 Hz, 1 H, Ar–*H*), 7.33 (t, *J* = 7.2 Hz, 1 H, Ar–*H*), 7.27 (t, *J* = 8.8 Hz, 2 H, Ar–*H*), 7.11 (t, *J* = 8.0 Hz, 1 H, Ar–*H*), 6.79 (d, *J* = 8.0 Hz, 1 H, Ar–*H*), 4.26 (s, 3 H, N–C*H*_3_), 1.69 (s, 15 H, C_5_(C*H*_3_)_5_). ^13^C NMR (100 MHz, CDCl_3_): δ 172.2, 140.5, 136.1, 134.3, 133.2, 132.2, 129.2, 127.9, 123.7, 123.5, 117.6, 111.3, 110.6, 110.0, 89.9, 51.3, 35.7, 9.2. IR, *ν*_max_ (cm^–1^) (CH_2_Cl_2_): 3485, 3059, 2970, 2915, 2221, 1943, 1601, 1483, 1452, 1435, 1381, 1359, 1343, 1287, 1266, 1241, 1194, 1141, 1129, 1092, 1028, 976, 828, 747, 662, 615, 589, 559, 546, 444.

#### Synthesis of Complex **2c**

Prepared according to procedure **2b** using **1c** (250 mg, 0.89 mmol), Ag_2_O (208 mg, 0.89 mmol), and [IrCl_2_Cp*]_2_ (358 mg, 0.45 mmol). Yield = 78%, 430 mg. Elemental analyses for C_22_H_26_Cl_2_IrN_3_ (595,11): C, 46.4570; H, 5.2603; N, 6.2050. ^1^H NMR (400 MHz, CDCl_3_): δ 7.80 (d, *J* = 8.0 Hz, 1 H, Ar–*H*), 7.66 (d, *J* = 7.6 Hz, 1 H, Ar–*H*), 7.55 (t, *J* = 7.6 Hz, 1 H, Ar–*H*), 7.39 (t, *J* = 7.6 Hz, 1 H, Ar–*H*), 6.94 (d, *J* = 2.0 Hz, 1 H, N–C*H*CH–N), 6.65 (d, *J* = 2.0 Hz, 1 H, N–CHC*H*–N), 5.07 (d, *J* = 14.4 Hz, 1 H, N–C*H*_2_), 4.02 (s, 3 H, N–C*H*_3_), 1.66 (s, 15 H, C_5_(C*H*_3_)_5_). ^13^C NMR (100 MHz, CDCl_3_): δ 157.9, 140.3, 133.6, 132.1, 130.8, 129.5, 123.9, 121.5, 117.6, 111.6, 90.6, 89.1, 51.8, 38.8, 9.2. IR, *ν*_max_ (cm^–1^) (CH_2_Cl_2_): 3414, 2955, 2912, 2219, 1656, 1603, 1453, 1403, 1379, 1301, 1241, 1191, 1116, 1077, 1030, 824, 763, 729, 692, 617, 415.

#### Synthesis of Complex **2d**

Prepared according to procedure **2b** using **1d** (250 mg, 0.99 mmol), Ag_2_O (228 mg, 0.99 mmol), and [IrCl_2_Cp*]_2_ (394 mg, 0.49 mmol). Yield = 61%, 430 mg. Elemental analyses for C_25_H_29_Cl_2_IrN_2_ (620,13): C, 45.0320; H, 4.9800; N, 4.7528. ^1^H NMR (400 MHz, CDCl_3_): δ 7.29 (m, 4 H, Ar–*H*), 7.25 (m, 1 H, Ar–*H*), 6.88 (d, *J* = 2.0 Hz, 1 H, N–C*H*CH–N), 6.63 (d, *J* = 2.0 Hz, 1 H, N–CHC*H*–N), 5.97 (d, *J* = 14.8 Hz, 1 H, N–C*H*_2_), 5.15 (d, *J* = 14.8 Hz, 1 H, N–C*H*_2_), 3.95 (s, 3 H, N-C*H*_3_), 1.58 (s, 15 H, C_5_(C*H*_3_)_5_). ^13^C NMR (100 MHz, CDCl_3_): δ 156.6, 136.8, 128.6, 128.5, 127.9, 123.4, 121.8, 88.8, 54.4, 38.7, 9.2. IR, *ν*_max_ (cm^–1^) (CH_2_Cl_2_): 3167, 3051, 3107, 2994, 2941, 2917, 1578, 1510, 1496, 1458, 1403, 1385, 1370, 1354, 1290, 1219, 1181, 1160, 1109, 1082, 1028, 993, 928, 837, 785, 739, 701, 689, 607, 578, 470, 462, 447.

#### Synthesis of Complex **2e**

Prepared according to procedure **2b** using **1e** (250 mg, 0.83 mmol), Ag_2_O (193 mg, 0.83 mmol), and [IrCl_2_Cp*]_2_ (398 mg, 0.41 mmol). Yield = 52%, 266 mg. ^1^H NMR (400 MHz, CDCl_3_): δ 7.62 (d, *J* = 7.6 Hz, 1 H, Ar–*H*), 7.45 (d, *J* = 7.6 Hz, 1 H, Ar–*H*), 7.34 (d, *J* = 7.6 Hz, 1 H, Ar–*H*), 7.23 (m, 3 H, Ar–*H*), 7.08 (d, *J* = 7.2 Hz, 1 H, Ar–*H*), 7.00 (t, *J* = 7.2 Hz, 1 H, Ar–*H*), 6.84 (t, *J* = 7.2 Hz, 1 H, Ar–*H*), 5.13 (d, *J* = 14.0 Hz, 1 H, N–C*H*_2_), 4.88 (d, *J* = 14.0 Hz, 1 H, N–C*H*_2_), 4.12 (s, 3 H, N–C*H*_3_), 1.71 (s, 15 H, C_5_(C*H*_3_)_5_). ^13^C NMR (100 MHz, CDCl_3_): δ 170.9, 143.3, 141.2, 138.0, 135.6, 134.0, 127.8, 124.5, 122.6, 122.3, 122.1, 109.9, 109.3, 91.2, 52.7, 33.9, 9.4. IR, *ν*_max_ (cm^–1^) (CH_2_Cl_2_): 3436, 3049, 2970, 2914, 1733, 1575, 1558, 1484, 1454, 1430, 1406, 1390, 1334, 1258, 1211, 1190, 1155, 1092, 1029, 853, 821, 756, 748, 575, 545, 435.

#### Synthesis of Complex **3a**

Prepared according to procedure **2a** using **1a** (250 mg, 0.75 mmol), Ag_2_O (175 mg, 0.75 mmol), and [RuCl_2_(*p*-cymene)]_2_ (231 mg, 0.38 mmol). Yield = 85%, 365 mg. Elemental analyze for C_25_H_24_Cl_2_N_2_RuS (556.51): C, 52.5850; H, 4.8610; N, 4.4582; S, 4.1602. ^1^H NMR (400 MHz, CDCl_3_): δ 7.78 (d, *J* = 8.4 Hz, 1 H, Ar–*H*), 7.74 (m, 1 H, Ar–*H*), 7.37 (m, 2 H, Ar–*H*), 7.32 (d, *J* = 7.6 Hz, 1 H, Ar–*H*), 7.28 (d, *J* = 7.6 Hz, 1 H, Ar–*H*), 7.13 (d, *J* = 8.0 Hz, 1 H, Ar–*H*), 6.98 (m, 1 H, Ar–*H*), 6.51 (s, 2 H, N–C*H*_2_), 5.46 (d, *J* = 6.0 Hz, 2 H, *p*-cymene-Ar–*H*), 5.31 (d, *J* = 5.6 Hz, 2 H, *p*-cymene-Ar–*H*), 2.87 (m, 1 H, *p*-cymene-C*H*), 2.16 (s, 3 H, p-cymene-C*H*_3_), 1.26 (d, *J* = 6.8 Hz, 6 H, p-cymene-(C*H*_3_)_2_). ^13^C NMR (100 MHz, CDCl_3_): δ 229.2, 143.8, 139.3, 136.4, 133.4, 132.9, 128.5, 128.2, 126.4, 124.9, 121.6, 117.1, 114.2, 110.1, 107.5, 100.8, 87.0, 86.9, 65.8, 55.8, 30.7, 22.3, 18.3, 15.2. IR, *ν*_max_ (cm^–1^) (CH_2_Cl_2_): 3447, 3038, 2963, 2221, 1629, 1598, 1457, 1377, 1315, 1202, 1162, 1141, 1113, 1081, 1055, 980, 908, 762, 752, 673, 551, 512, 448.

#### Synthesis of Complex **3b**

Prepared according to procedure **2b** using **1b** (250 mg, 0.76 mmol), Ag_2_O (177 mg, 0.76 mmol), and [RuCl_2_(*p*-cymene)]_2_ (233 mg, 0.38 mmol). Yield = 83%, 360 mg. Elemental analyses for C_26_H_27_Cl_2_N_3_Ru (553.06): C, 54.7640; H, 5.3754; N, 7.2315. ^1^H NMR (400 MHz, CDCl_3_): δ 7.68 (d, *J* = 7.2 Hz, 1 H, Ar–*H*), 7.41 (d, *J* = 8.0 Hz, 1 H, Ar–*H*), 7.35 (m, 2 H, Ar–*H*), 7.30 (m, 1 H, Ar–*H*), 7.15 (d, *J* = 8.0 Hz, 1 H, Ar–*H*), 7.12 (m, 1 H, Ar–*H*), 6.79 (d, *J* = 8.0 Hz, 1 H, N–C*H*_2_), 5.53 (d, *J* = 2.8 Hz, 2 H, *p*-cymene-Ar–*H*), 5.23 (d, *J* = 6.0 Hz, 2 H, *p*-cymene-Ar–*H*), 4.29 (s, 3 H, *N*-C*H*_3_), 3.01 (m, 1 H, *p*-cymene-C*H*), 2.09 (s, 3 H, *p*-cymene-C*H*_3_), 1.28 (d, *J* = 7.2 Hz, 6 H, *p*-cymene-(C*H*_3_)_2_). ^13^C NMR (100 MHz, CDCl_3_): δ 191.5, 140.5, 136.2, 134.5, 133.1, 132.3, 129.4, 127.9, 123.5, 123.3, 117.6, 110.9, 110.4, 110.2, 110.0, 99.2, 86.5, 83.5, 51.7, 36.8, 30.9, 29.7, 22.5, 18.8. IR, *ν*_max_ (cm^–1^) (CH_2_Cl_2_): 3446, 3379, 3039, 2958, 2221, 1887, 1600, 1461, 1378, 1350, 1265, 1193, 1127, 1089, 1030, 973, 925, 842, 752, 664, 558, 442.

#### Synthesis of Complex **3c**

Prepared according to procedure **2b** using **1c** (250 mg, 0.89 mmol), Ag_2_O (208 mg, 0.89 mmol), and [RuCl_2_(*p*-cymene)]_2_ (275 mg, 0.45 mmol). Yield = 86%, 403 mg. Elemental analyses for C_22_H_25_Cl_2_N_3_Ru (503.05): C, 49.1370; H, 5.2781; N, 8.0532. ^1^H NMR (400 MHz, CDCl_3_): δ 7.65 (t, *J* = 8.0 Hz, 2 H, Ar–*H*), 7.51 (t, *J* = 8.0 Hz, 1 H, Ar–*H*), 7.38 (t, *J* = 7.6 Hz, 1 H, Ar–*H*), 6.99 (d, *J* = 2.0 Hz, 1 H, N–C*H*CH–N), 7.66 (d, *J* = 2.0 Hz, 1 H, N–CHC*H*–N), 5.47 (d, *J* = 4.8 Hz, 2 H, *p*-cymene-Ar–*H*), 5.20 (d, *J* = 6.0 Hz, 2 H, *p*-cymene-Ar–*H*), 4.05 (s, 3 H, N–C*H*_3_), 2.95 (m, 1 H, *p*-cymene–C*H*), 2.10 (s, 3 H, *p*-cymene–C*H*_3_), 1.27 (d, *J* = 6.8 Hz, 6 H, *p*-cymene–(C*H*_3_)_2_). ^13^C NMR (100 MHz, CDCl_3_): δ 230.7, 178.1, 133.5, 132.3, 131.0, 128.6, 124.4, 121.9, 111.7, 109.0, 99.0, 83.1, 52.5, 39.8, 30.9, 18.7. IR, *ν*_max_ (cm^–1^) (CH2Cl2): 3414, 3090, 2959, 2222, 1617, 1549, 1450, 1384, 1232, 1084, 865, 769, 689, 618, 468.

#### Synthesis of Complex **3d**

Prepared according to procedure **2b** using **1d** (250 mg, 0.99 mmol), Ag_2_O (228 mg, 0.99 mmol), and [RuCl_2_(*p*-cymene)]_2_ (302 mg, 0.49 mmol). Yield = 57%, 403 mg. Elemental analyses for C_21_H_26_Cl_2_N_2_Ru (478.05): C, 48.8320; H, 5.8370; N, 5.6175. ^1^H NMR (400 MHz, CDCl_3_): δ 7.32 (m, 3 H, Ar–*H*), 7.25 (m, 2 H, Ar–*H*), 6.97 (d, *J* = 2.0 Hz, 1 H, N–C*H*CH–N), 7.81 (d, *J* = 2.0 Hz, 1 H, N–CHC*H*–N), 5.65 (s, 2 H, N–C*H*_2_), 5.30 (s, 2 H, *p*-cymene–Ar–*H*), 4.97 (s, 2 H, *p*-cymene–Ar–*H*), 3.99 (s, 3 H, N-C*H*_3_), 2.88 (m, 1 H, *p*-cymene–C*H*), 2.01 (s, 3 H, *p*-cymene–C*H*_3_), 1.21 (d, *J* = 6.8 Hz, 6 H, *p*-cymene–(C*H*_3_)_2_). ^13^C NMR (100 MHz, CDCl_3_): δ 174.3, 137.6, 128.8, 127.9, 127.6, 123.9, 122.9, 108.5, 98.8, 54.7, 39.7, 30.7, 18.7. IR, *ν*_max_ (cm^–1^) (CH_2_Cl_2_): 3527, 3180, 3140, 3126, 3103, 3090, 3057, 3028, 2964, 2922, 2872, 1965, 1869, 1825, 1664, 1628, 1602, 1582, 1558, 1495, 1454, 1403, 1386, 1372, 1352, 1331, 1276, 1225, 1138, 1114, 1082, 1056, 1008, 990, 888, 836, 733, 726, 702, 682, 635, 621, 608, 470.

#### General Procedure for the *N*-Alkylation of Aniline with Alcohols

A Radley’s tube was charged with aniline (1.0 mmol), alcohols (1.0 mmol), KO^*t*^Bu (1.5 mmol), and catalyst (1.0 mol %). The mixture was stirred to 120 °C for 24 h under air atmosphere. The reaction mixture was cooled to room temperature and filtered.

#### General Method for the *N*-Methylation of Anilines with Methanol

A Radley’s tube was charged with aniline (1.0 mmol), KO^*t*^Bu (1.5 mmol), and catalyst (1.0 mol %) in methanol (0.4 mL). The mixture was stirred to 120 °C for 24 h under air atmosphere. The reaction mixture was cooled to room temperature and filtered.

### *N*-Alkylation of Aniline with Alcohol Products^42−51^

#### *N*-Benzylaniline (**12a**)

^1^H NMR (400 MHz, CDCl_3_): δ 7.38 (m, 4 H, Ar–*H*), 7.30 (m, 1 H, Ar–*H*), 7.20 (m, 2 H, Ar–*H*), 6.74 (t, *J* = 7.2 Hz, 1 H, Ar–*H*), 6.67 (m, 2 H, Ar–*H*), 4.35 (s, 2 H, C*H*_2_), 4.04 (s, 1 H, N–*H*). ^13^C NMR (100 MHz, CDCl_3_): δ 148.2, 139.4, 129.3, 128.6, 127.5, 127.2, 117.6, 112.8, 48.3.

#### *N*-Benzyl-4-toluidine (**12b**)

^1^H NMR (400 MHz, CDCl_3_): δ 7.36 (m, 4 H, Ar–*H*), 7.27 (m, 1 H, Ar–*H*), 6.99 (d, *J* = 8.4 Hz, 2 H, Ar–*H*), 6.58 (m, 2 H, Ar–*H*), 4.32 (s, 2 H, C*H*_2_), 3.91 (s, 1 H, N–*H*), 2.25 (s, 3 H, C*H*_3_). ^13^C NMR (100 MHz, CDCl_3_): δ 145.9, 139.6, 129.7, 129.6, 128.6, 128.5, 127.5, 127.4, 127.1, 126.7, 112.9, 48.6, 20.4.

#### *N*-Benzyl-2-toluidine (**12c**)

^1^H NMR (400 MHz, CDCl_3_): δ 7.56 (m, 3 H, Ar–*H*), 7.52 (m, 2 H, Ar–*H*), 7.46 (m, 1 H, Ar–*H*), 7.29 (m, 1 H, Ar–*H*), 6.88 (t, *J* = 7.2 Hz, 1 H, Ar–*H*), 6.80 (d, *J* = 8.0 Hz, 1 H, Ar–*H*), 4.53 (s, 2 H, C*H*_2_), 4.02 (s, 1 H, N–*H*), 2.34 (s, 3 H, C*H*_3_). ^13^C NMR (100 MHz, CDCl_3_): δ 146.2, 139.7, 130.3, 128.8, 127.9, 127.7, 127.4, 127.3, 122.1, 117.4, 110.2, 48.4, 17.7.

#### *N*-Benzyl-2,4-dimethylaniline (**12d**)

^1^H NMR (400 MHz, CDCl_3_): δ 7.51 (m, 1 H, Ar–*H*), 7.35 (m, 3 H, Ar–*H*), 7.04 (m, 3 H, Ar–*H*), 6.86 (t, *J* = 7.6 Hz, 1 H, Ar–*H*), 4.12 (s, 2 H, C*H*_2_), 2.29 (s, 6 H, (C*H*_3_)_2_). ^13^C NMR (100 MHz, CDCl_3_): δ 144.7, 140.8, 135.6, 128.5, 127.6, 126.9, 126.2, 118.8, 116.9, 65.3, 52.8.

#### *N*-Benzyl-2-nitroaniline (**12e**)

^1^H NMR (400 MHz, CDCl_3_): δ 8.10 (d, *J* = 8.4 Hz, 1 H, Ar–*H*), 7.35 (m, 4 H, Ar–*H*), 6.80 (d, *J* = 8.4 Hz, 1 H, Ar–*H*), 6.69 (m, 1 H, Ar–*H*), 3.33 (s, 2 H, C*H*_2_).^13^C NMR (100 MHz, CDCl_3_): δ 144.7, 135.6, 128.5, 128.4, 128.2, 127.6, 126.9, 126.7, 126.1, 118.8, 116.9, 52.8.

#### *N*-Benzyl-4-methoxyaniline (**12f**)

^1^H NMR (400 MHz, CDCl_3_): δ 7.42 (m, 4 H, Ar–*H*), 7.33 (m, 1 H, Ar–*H*), 6.88 (m, 2 H, Ar–*H*), 6.74 (t, *J* = 7.2 Hz, 1 H, Ar–*H*), 6.66 (d, *J* = 7.6 Hz, 1 H, Ar–*H*), 4.69 (s, 1 H, N–*H*), 4.41 (s, 2 H, C*H*_2_), 3.90 (s, 3 H, C*H*_3_). ^13^C NMR (100 MHz, CDCl_3_): δ 146.8, 139.6, 138.2, 128.6, 127.5, 127.1, 121.3, 116.7, 110.1, 109.4, 55.4, 48.1.

#### *N*-Benzyl-2-methoxyaniline (**12g**)

^1^H NMR (400 MHz, CDCl_3_): δ 7.39 (m, 4 H, Ar–*H*), 7.31 (m, 1 H, Ar–*H*), 6.82 (m, 2 H, Ar–*H*), 6.64 (m, 2 H, Ar–*H*), 4.31 (s, 2 H, C*H*_2_), 3.77 (s, 3 H, C*H*_3_).^13^C NMR (100 MHz, CDCl_3_): δ 152.2, 142.5, 139.7, 128.6, 127.6, 127.2, 114.9, 114.1, 55.8, 49.3.

#### *N*-Benzyl-4-bromoaniline (**12h**)

^1^H NMR (400 MHz, CDCl_3_): δ 7.36 (d, *J* = 4.8 Hz, 4 H, Ar–*H*), 7.27 (m, 3 H, Ar–*H*), 6.51 (m, 2 H, Ar–*H*), 4.31 (s, 2 H, C*H*_2_), 4.08 (s, 1 H, N–*H*). ^13^C NMR (100 MHz, CDCl_3_): δ 147.1, 138.9, 131.9, 128.7, 127.4, 127.3, 114.4, 109.1, 48.2.

#### *N*-Benzyl-4-chloroaniline (**12i**)

^1^H NMR (400 MHz, CDCl_3_): δ 7.36 (m, 5 H, Ar–*H*), 7.16 (m, 2 H, Ar–*H*), 6.58 (m, 2 H, Ar–*H*), 4.33 (s, 2 H, C*H*_2_), 4.08 (s, 1 H, N–*H*). ^13^C NMR (100 MHz, CDCl_3_): δ 146.7, 138.9, 129.1, 128.7, 127.5, 127.4, 122.1, 113.9, 48.4.

#### *N*-benzyl-hexylamine (**12j**)

^1^H NMR (400 MHz, CDCl_3_): δ 7.32 (d, *J* = 4.4 Hz, 4 H, Ar–*H*), 7.25 (m, 1 H, Ar–*H*), 3.79 (s, 2 H, C*H*_2_), 2.63 (t, *J* = 7.2 Hz, 2 H, NH–C*H*_2_), 1.53 (m, 2 H, NHCH_2_C*H*_2_), 1.33 (m, 6 H, NHCH_2_CH_2_(C*H*_2_)_3_), 0.90 (t, *J* = 6.8 Hz, 3 H, NHCH_2_CH_2_(CH_2_)_3_–C*H*_3_). ^13^C NMR (100 MHz, CDCl_3_): δ 140.6, 128.3, 128.1, 126.8, 54.1, 49.5, 31.8, 30.1, 27.1, 22.6, 14.1.

#### *N*-Benzyl-2-naphthylamine (**12k**)

^1^H NMR (400 MHz, CDCl_3_): δ 7.81 (d, *J* = 8.0 Hz, 1 H, Ar–*H*), 7.74 (t, *J* = 9.2 Hz, 2 H, Ar–*H*), 7.48 (m, 5 H, Ar–*H*), 7.41 (m, 1 H, Ar–*H*), 7.33 (m, 1 H, Ar–*H*), 6.96 (m, 2 H, Ar–*H*), 4.49 (s, 2 H, C*H*_2_), 4.21 (s, 1 H, N–*H*). ^13^C NMR (100 MHz, CDCl_3_): δ 145.9, 139.3, 135.3, 129.1, 128.8, 127.8, 127.7, 127.4, 126.5, 126.1, 122.2, 117.9, 104.8, 48.4.

#### *N*-Benzyl-4-biphenylamine (**12l**)

^1^H NMR (400 MHz, CDCl_3_): δ 7.46 (m, 4 H, Ar–*H*), 7.35 (m, 5 H, Ar–*H*), 7.22 (m, 2 H, Ar–*H*), 7.13 (d, *J* = 7.6 Hz, 1 H, Ar–*H*), 6.79 (t, *J* = 7.6 Hz, 1 H, Ar–*H*), 6.67 (d, *J* = 8.4 Hz, 1 H, Ar–*H*), 4.41 (s, 1 H, N–*H*), 4.34 (s, 2 H, C*H*_2_). ^13^C NMR (100 MHz, CDCl_3_): δ 144.9, 139.5, 139.4, 130.2, 129.4, 128.9, 128.7, 128.6, 127.7, 127.2, 127.0, 117.1, 110.7, 48.1.

#### *N*-(4-Methylbenzyl)aniline (**13a**)

^1^H NMR (400 MHz, CDCl_3_): δ 7.32 (d, *J* = 8.0 Hz, 2 H, Ar–*H*), 7.24 (m, 4 H, Ar–*H*), 6.78 (m, 1 H, Ar–*H*), 6.69 (m, 2 H, Ar–*H*), 4.33 (s, 2 H, C*H*_2_), 4.02 (s, 1 H, N–*H*), 2.41 (s, 3 H, C*H*_3_). ^13^C NMR (100 MHz, CDCl_3_): δ 148.3, 136.9, 136.4, 129.4, 129.3, 127.6, 117.5, 112.9, 48.1, 21.2.

#### *N*-(4-Bromobenzyl)aniline (**13b**)

^1^H NMR (400 MHz, CDCl_3_): δ 7.47 (m, 2 H, Ar–*H*), 7.26 (m, 2 H, Ar–*H*), 7.18 (m, 2 H, Ar–*H*), 6.74 (m, 1 H, Ar–*H*), 6.62 (d, *J* = 8.8 Hz, 2 H, Ar–*H*), 4.30 (s, 2 H, C*H*_2_), 4.06 (s, 1 H, N–*H*). ^13^C NMR (100 MHz, CDCl_3_): δ 147.8, 138.5, 131.7, 129.3, 129.0, 120.9, 117.8, 112.9, 47.7.

#### *N*-(2-Bromobenzyl)aniline (**13c**)

^1^H NMR (400 MHz, CDCl_3_): δ 7.44 (m, 2 H, Ar–*H*), 7.23 (m, 4 H, Ar–*H*), 6.77 (m, 1 H, Ar–*H*), 6.66 (m, 2 H, Ar–*H*), 4.47 (s, 2 H, C*H*_2_), 4.17 (s, 1 H, N–*H*). ^13^C NMR (100 MHz, CDCl_3_): δ 147.9, 136.9, 133.3, 132.3, 129.6, 129.4, 129.1, 128.5, 121.3, 117.9, 113.0, 45.9.

#### *N*-(4-Chlorobenzyl)aniline (**13d**)

^1^H NMR (400 MHz, CDCl_3_): δ 7.39 (m, 4 H, Ar–*H*), 7.29 (m, 2 H, Ar–*H*), 6.85 (m, 1 H, Ar–*H*), 6.70 (m, 2 H, Ar–*H*), 4.36 (s, 2 H, C*H*_2_), 4.10 (s, 1 H, N–*H*). ^13^C NMR (100 MHz, CDCl_3_): δ 147.9, 138.2, 132.9, 129.4, 129.1, 128.9, 128.8, 117.9, 113.0, 47.6.

#### *N*-(4-Methoxybenzyl)aniline (**13e**)

^1^H NMR (400 MHz, CDCl_3_): δ 7.37 (d, *J* = 8.4 Hz, 2 H, Ar–*H*), 7.26 (m, 2 H, Ar–*H*), 6.97 (m, 2 H, Ar–*H*), 6.81 (m, 1 H, Ar–*H*), 6.71 (m, 2 H, Ar–*H*), 4.32 (s, 2 H, C*H*_2_), 4.02 (s, 1 H, N–*H*), 3.87 (s, 3 H, C*H*_3_). ^13^C NMR (100 MHz, CDCl_3_): δ 158.9, 148.3, 131.5, 129.3, 128.9, 117.5, 114.1, 112.9, 55.3, 47.8.

#### *N*-(2,4,6-trimethylbenzyl)aniline (**13f**)

^1^H NMR (400 MHz, CDCl_3_): δ 7.37 (m, 2 H, Ar–*H*), 7.06 (s, 2 H, Ar–*H*), 6.89 (t, *J* = 6.8 Hz, 1 H, Ar–*H*), 6.80 (d, *J* = 8.0 Hz, 2 H, Ar–*H*), 4.34 (s, 2 H, C*H*_2_), 3.54 (s, 1 H, N–*H*), 2.51 (s, 6 H, (C*H*_3_)_2_), 2.46 (s, 3 H, C*H*_3_). ^13^C NMR (100 MHz, CDCl_3_): δ 148.8, 137.6, 137.4, 132.4, 129.4, 129.2, 117.4, 112.6, 42.5, 21.1, 19.6.

## Supporting Information Available

^1^H and ^13^C NMR spectra; IR spectrum; ^1^H NMR monitoring of *N*-methylation of aniline with methanol; elemental analysis results of the complexes **2a**–**d** and **3a**–**d**; and crystal data and structure refinement parameters for complexes **2b**, **3a**, and **3c** (PDF)

## Accession Codes

Crystallographic data for the structural analysis have been deposited with the Cambridge Crystallographic Data Centre, CCDC Nos. 1935381, 2165787, and 2165788. Copies of this information may be obtained free of charge from the Director, CCDC, 12 Union Road, Cambridge CB2 1EZ, U.K. (fax: +44-1223-336033; e-mail: deposit@ccdc.cam.ac.uk or www:http://www.ccdc.cam.ac.uk).
