# Supplementary material for: N-Alkylation and N-Methylation of Amines with Alcohols Catalyzed by Nitrile-Substituted NHC–Ir(III) and NHC–Ru(II) Complexes
Source: ACS Omega. 2023 Feb 2;8(6):5332–48. doi: 10.1021/acsomega.2c06341 (PMC9933218; doi:10.1021/acsomega.2c06341)
Supplement: Supplementary file 1 — ao2c06341_si_001.pdf [file ao2c06341_si_001.pdf]

Supporting Information

N-alkylation and *N*-methylation of amines with alcohols catalyzed by nitrile substituted NHC-Ir(III) and -Ru(II) complexes

Sinem Çakır[a], Serdar Batıkan Kavukcu[a], Onur Şahin[b], Salih Günnaz[a], Hayati Türkmen[a]*

[a] Department of Chemistry, Ege University, 35100 Bornova-Izmir, Turkey

[b] Department of Occupat Health & Safety, Faculty of Health Sciences, Sinop University, Sinop, Türkiye

e-mail: hayatiturkmen@hotmail.com

Contents

^{1}H- and ^{13}C- NMR spectra of **1e** 2

^{1}H- and ^{13}C- NMR spectra of **2a-e, 3a-d** 3-11

^{1}H- and ^{13}C- NMR spectra of **catalytic products** 12-29

Elemental analysis results of the complexes **2a-d** and **3a-d** 30

FTIR spectra of **1e**, **2a-e, 3a-d** 31-35

Crystal data and structure refinement parameters for **2b, 3a** and **3c** 36

^{1}H-NMR monitoring of *N*-alkylation of aniline with benzyl alcohol (24h) 37

^{1}H-NMR monitoring of *N*-alkylation of aniline with methanol (24h) 38

^{1}H-NMR spectra of *N*-methylaniline and *N*-(methyl-d_3)aniline-*d* 39

^{13}C-NMR spectra of *N*-methylaniline and *N*-(methyl-d_3)aniline-*d* 40

FTIR spectra of **2b** and **2b’** 41

^{1}H- and ^{13}C- NMR spectras of the compound **1e**

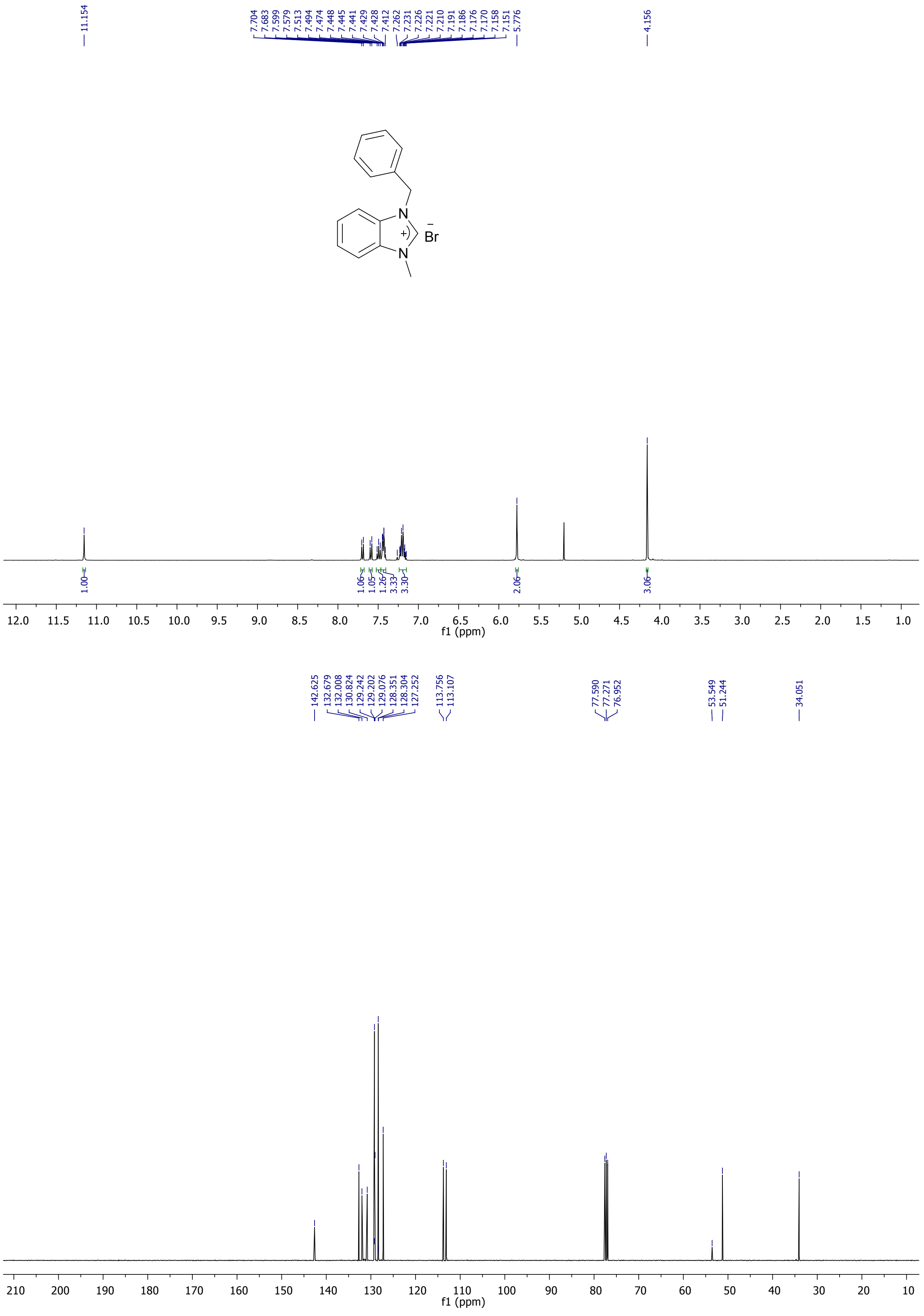

Figure S1. ^{1}H and ^{13}C NMR spectra of complex **1e** ($CDCl_3$).

^{1}H- and ^{13}C- NMR spectras of the complexes **2a-e, 3a-d**

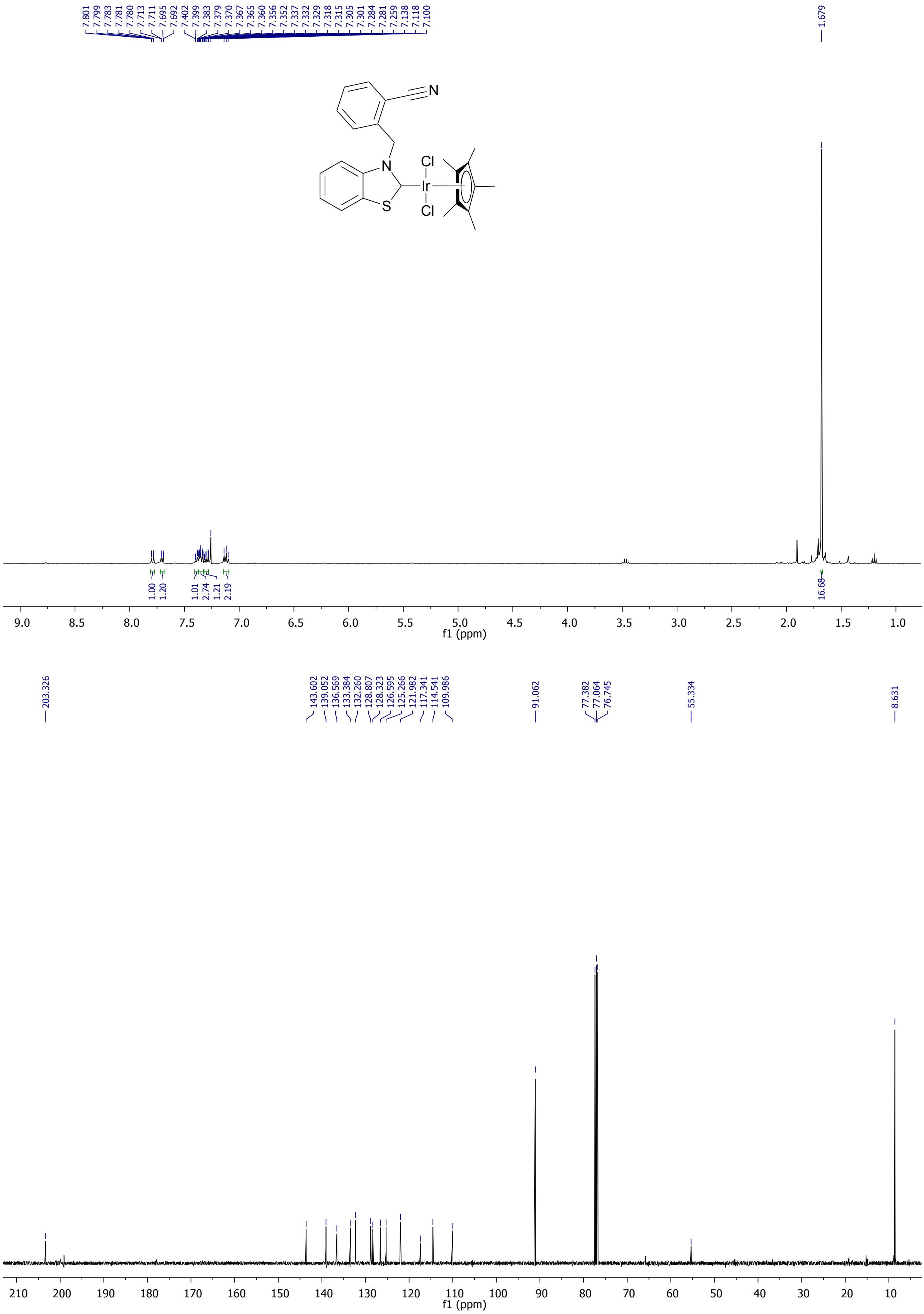

Figure S2. ^{1}H and ^{13}C NMR spectra of complex **2a** ($CDCl_3$).

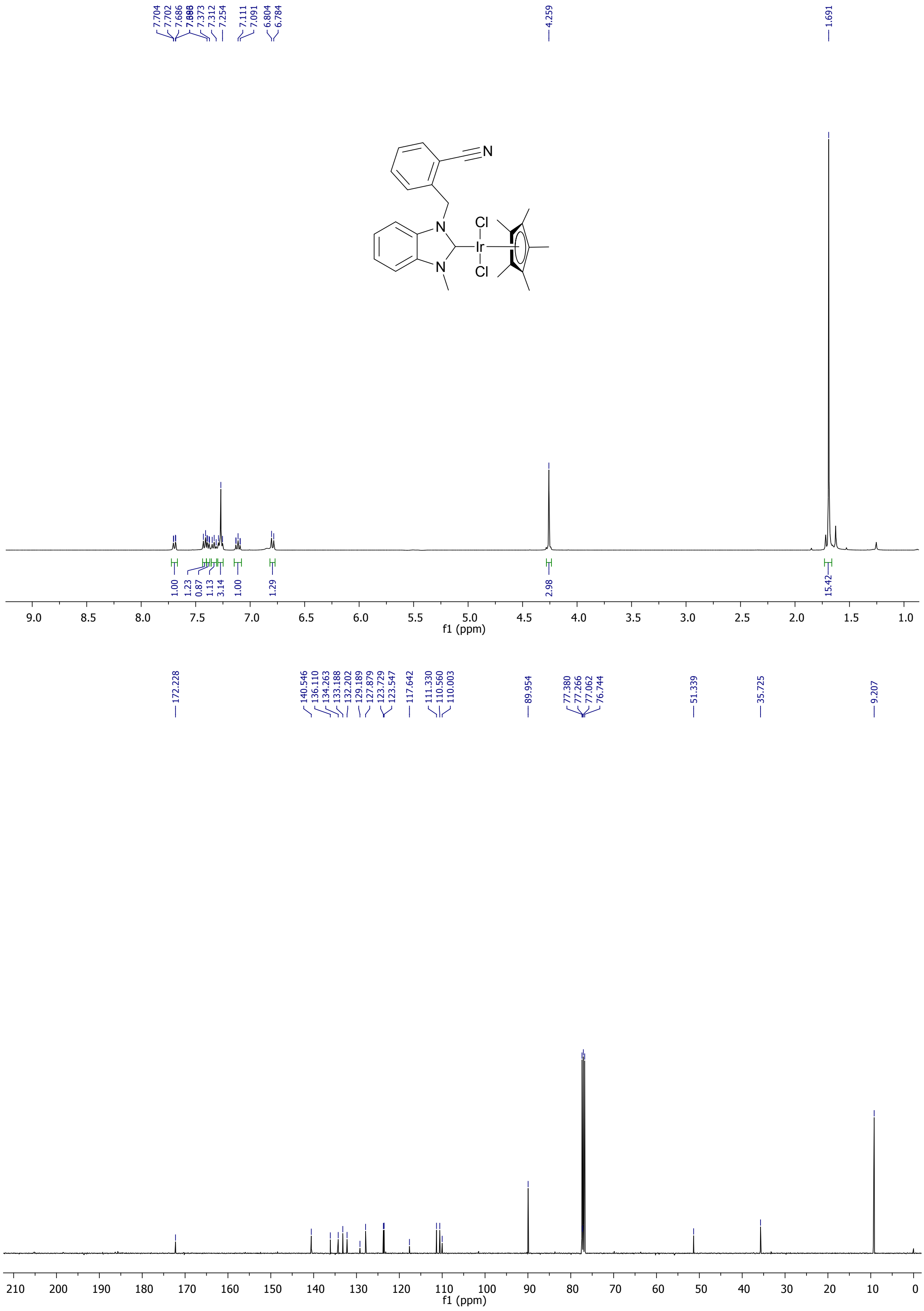

Figure S3. ^1H and ^{13}C NMR spectra of complex **2b** ($CDCl_3$).

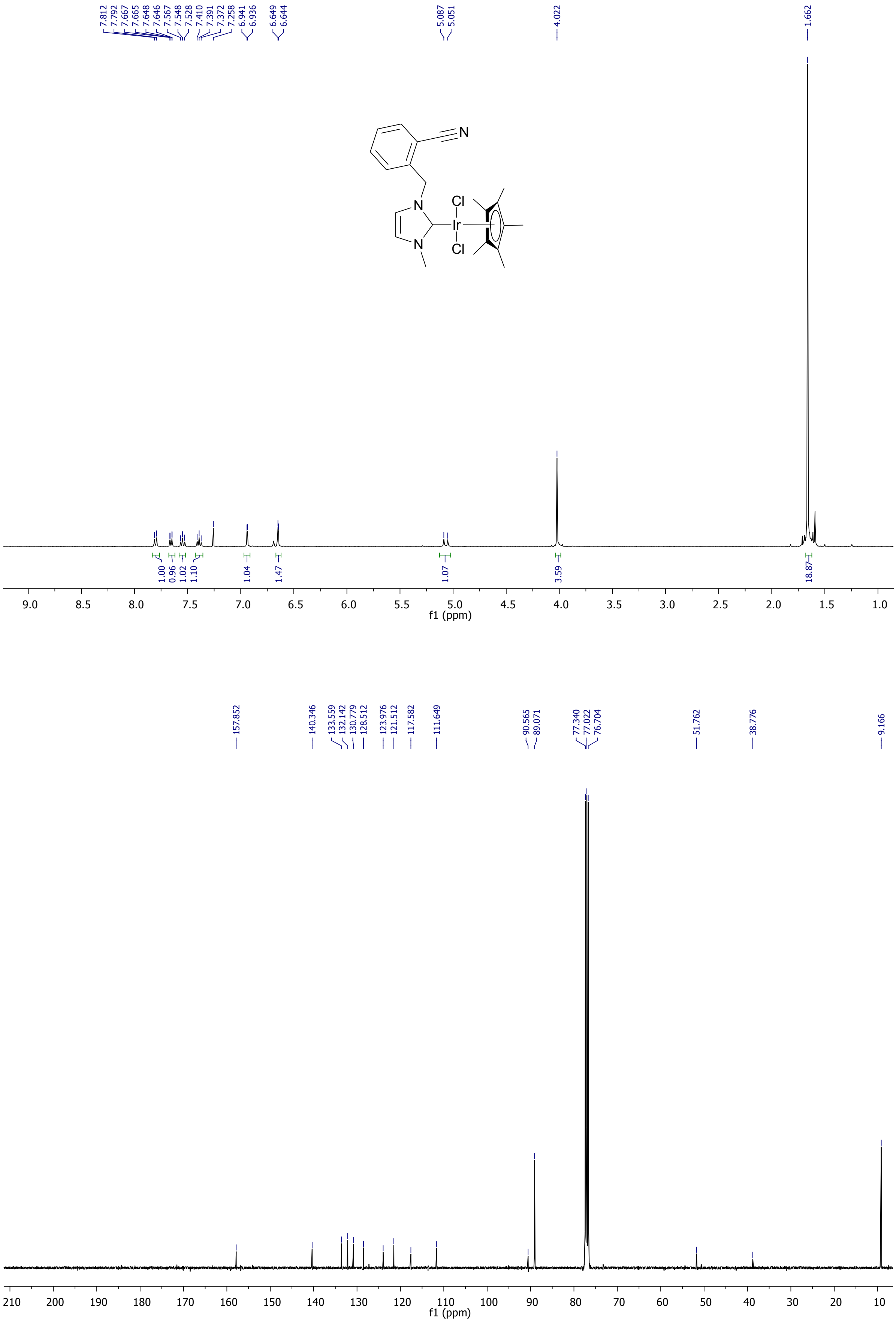

Figure S4. ^{1}H and ^{13}C NMR spectra of complex **2c** ($CDCl_3$).

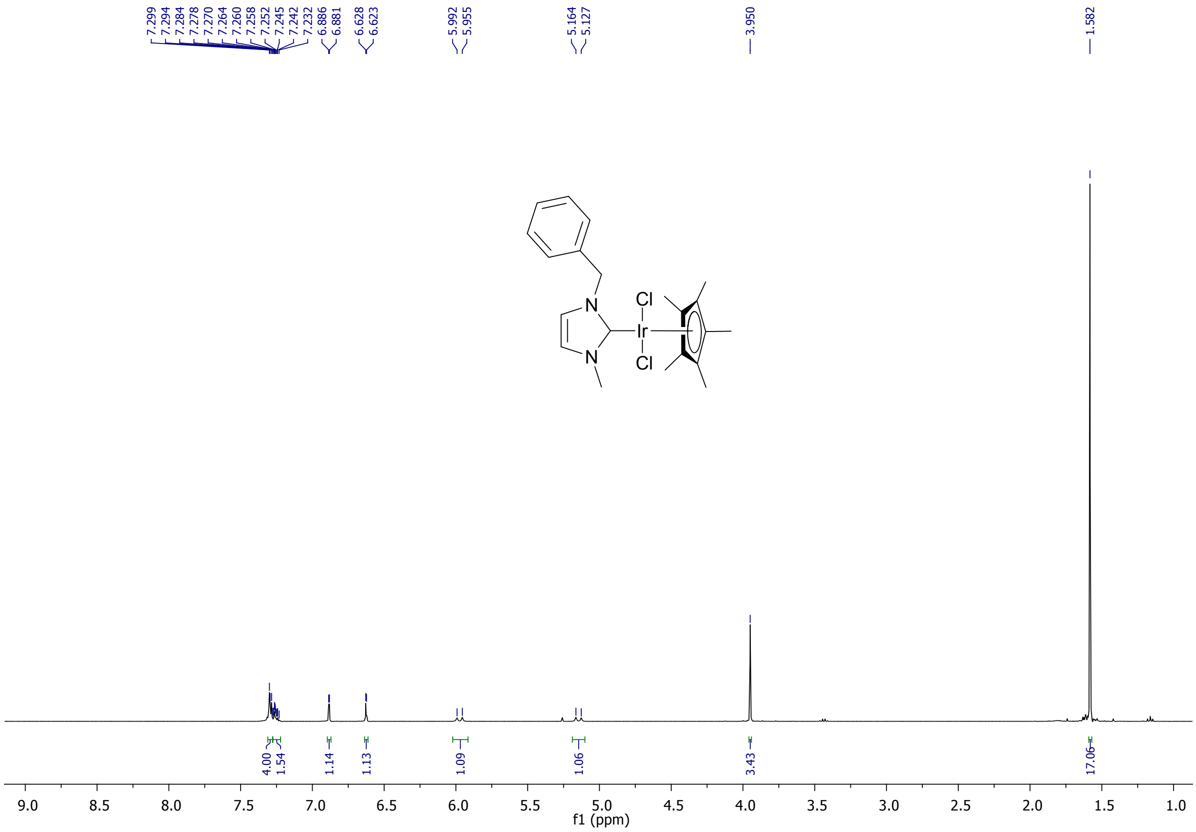

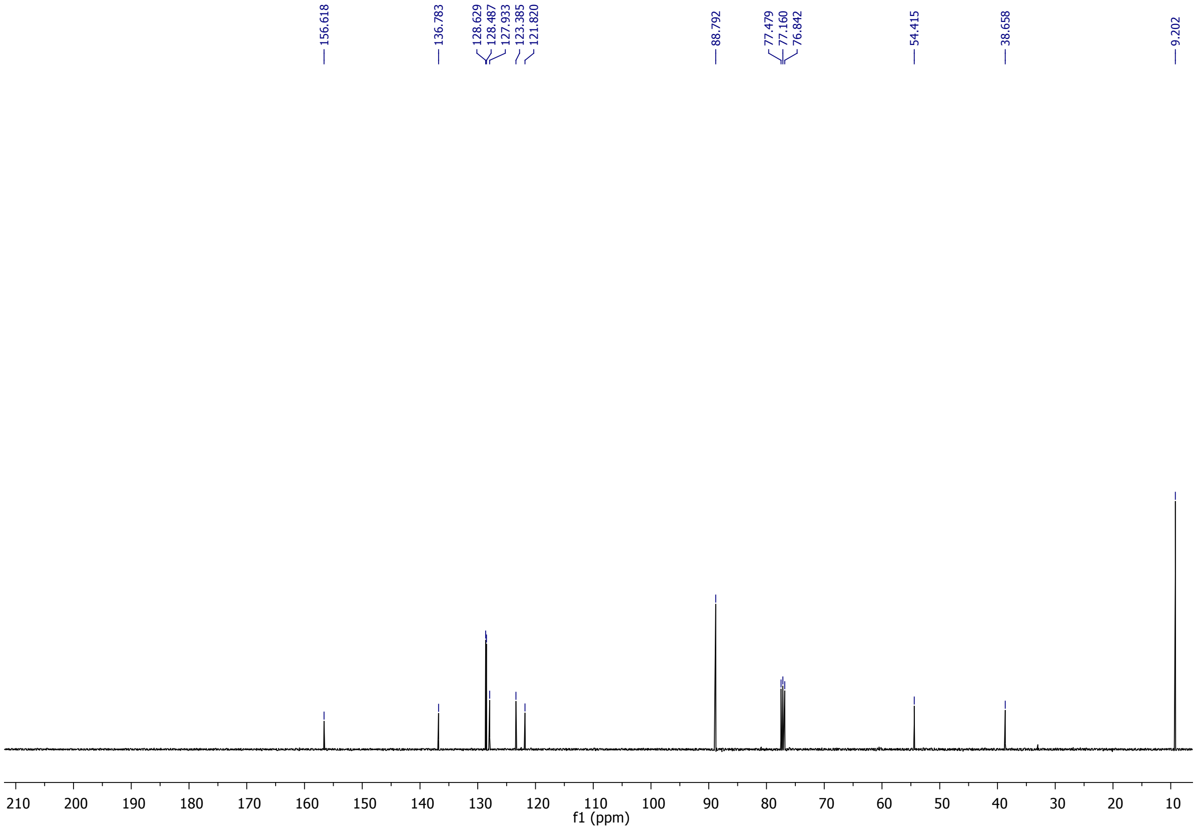

Figure S5. ^{1}H and ^{13}C NMR spectra of complex **2d** ($CDCl_3$).

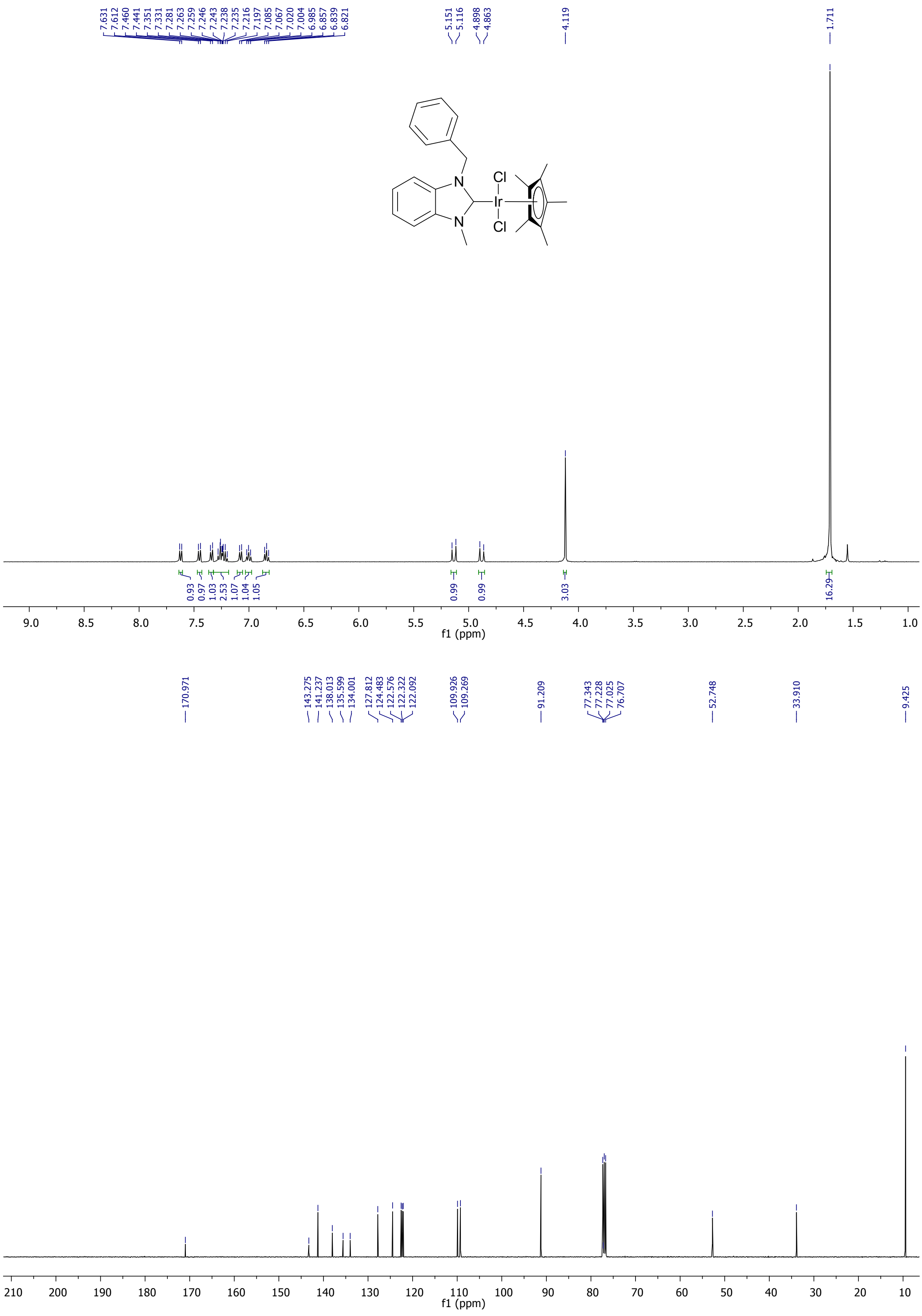

Figure S6. ^{1}H and ^{13}C NMR spectra of complex **2e** ($CDCl_3$).

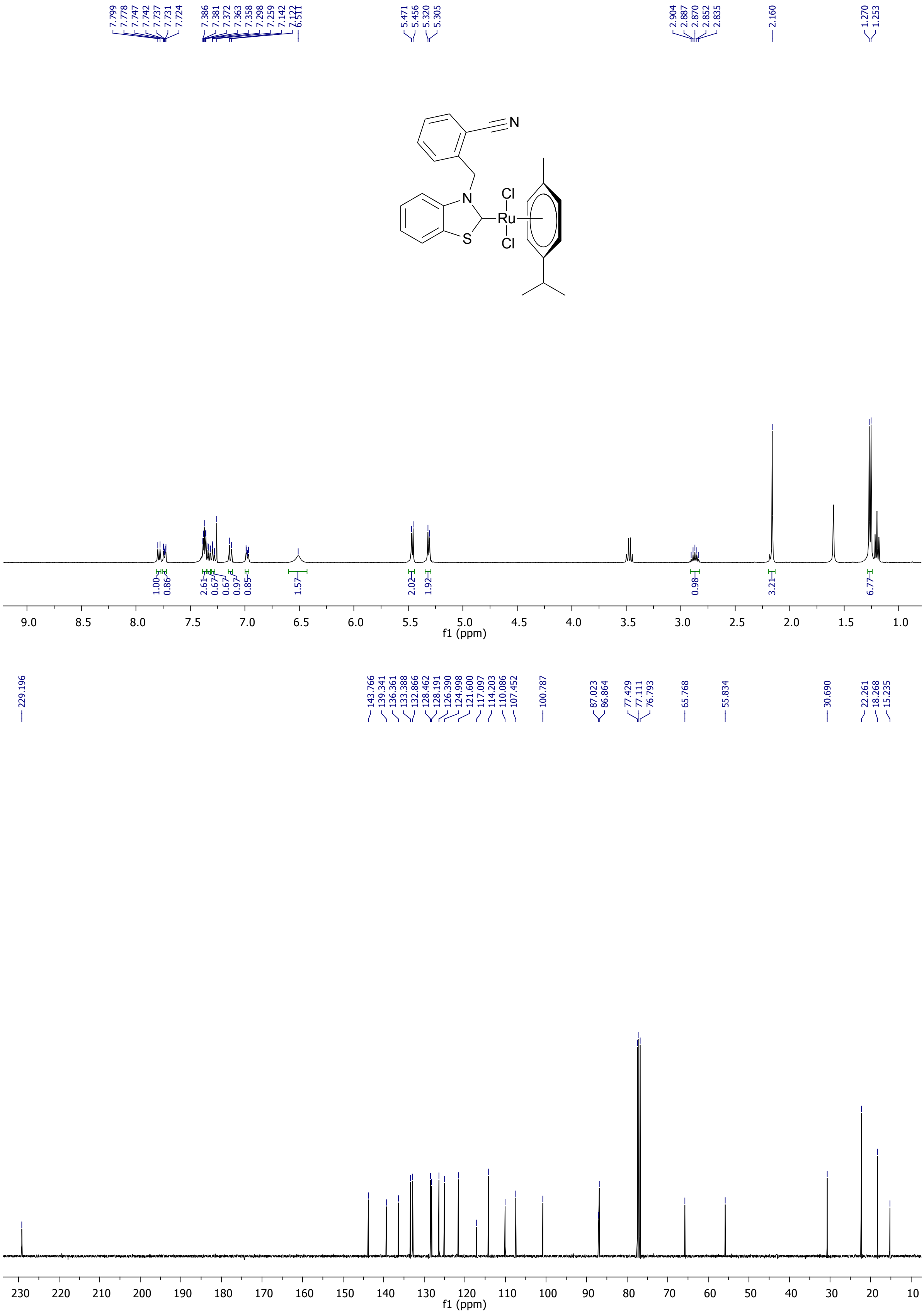

Figure S7. ^{1}H and ^{13}C NMR spectra of complex **3a** ($CDCl_3$).

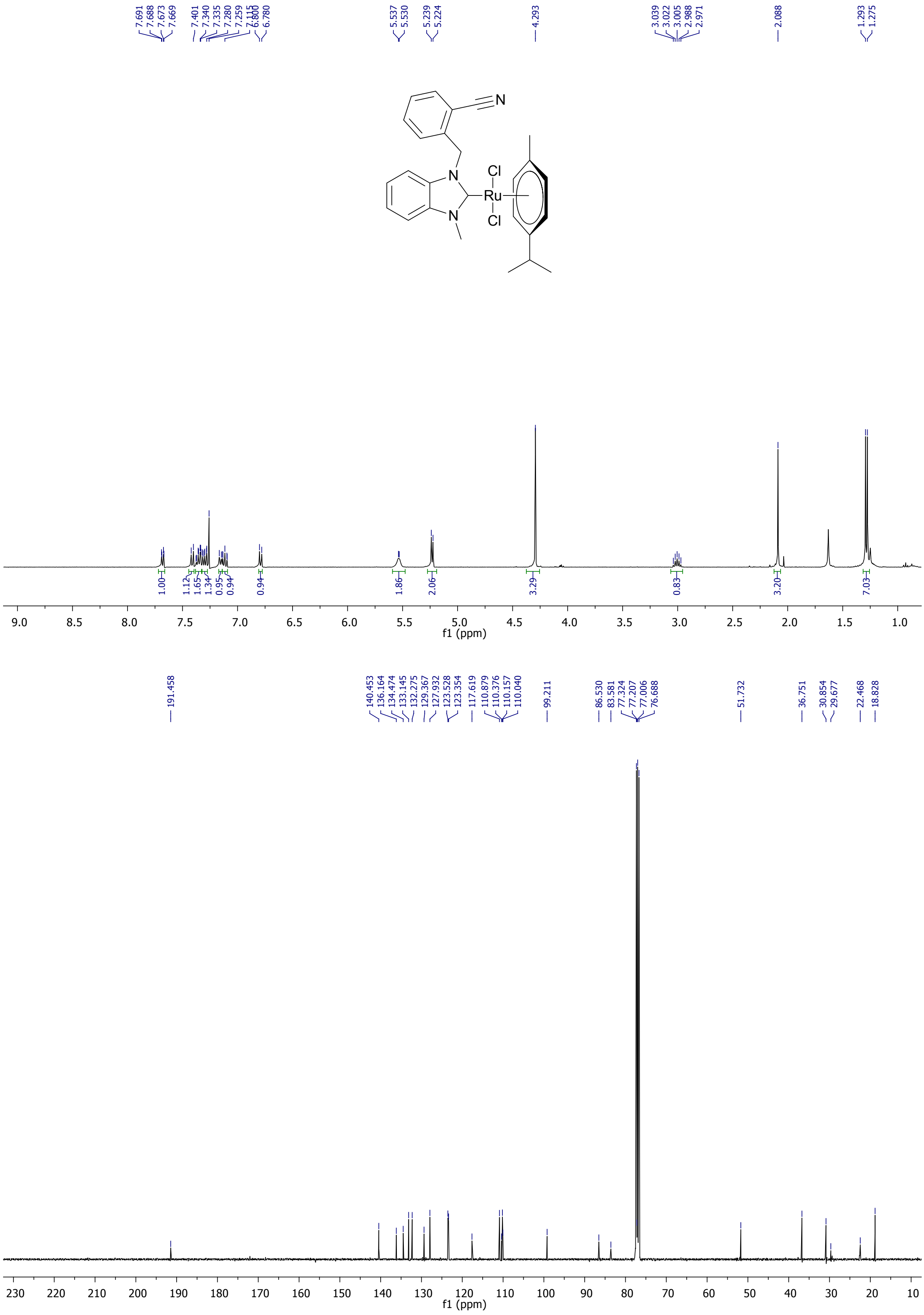

Figure S8. ^{1}H and ^{13}C NMR spectra of complex **3b** ($CDCl_3$).

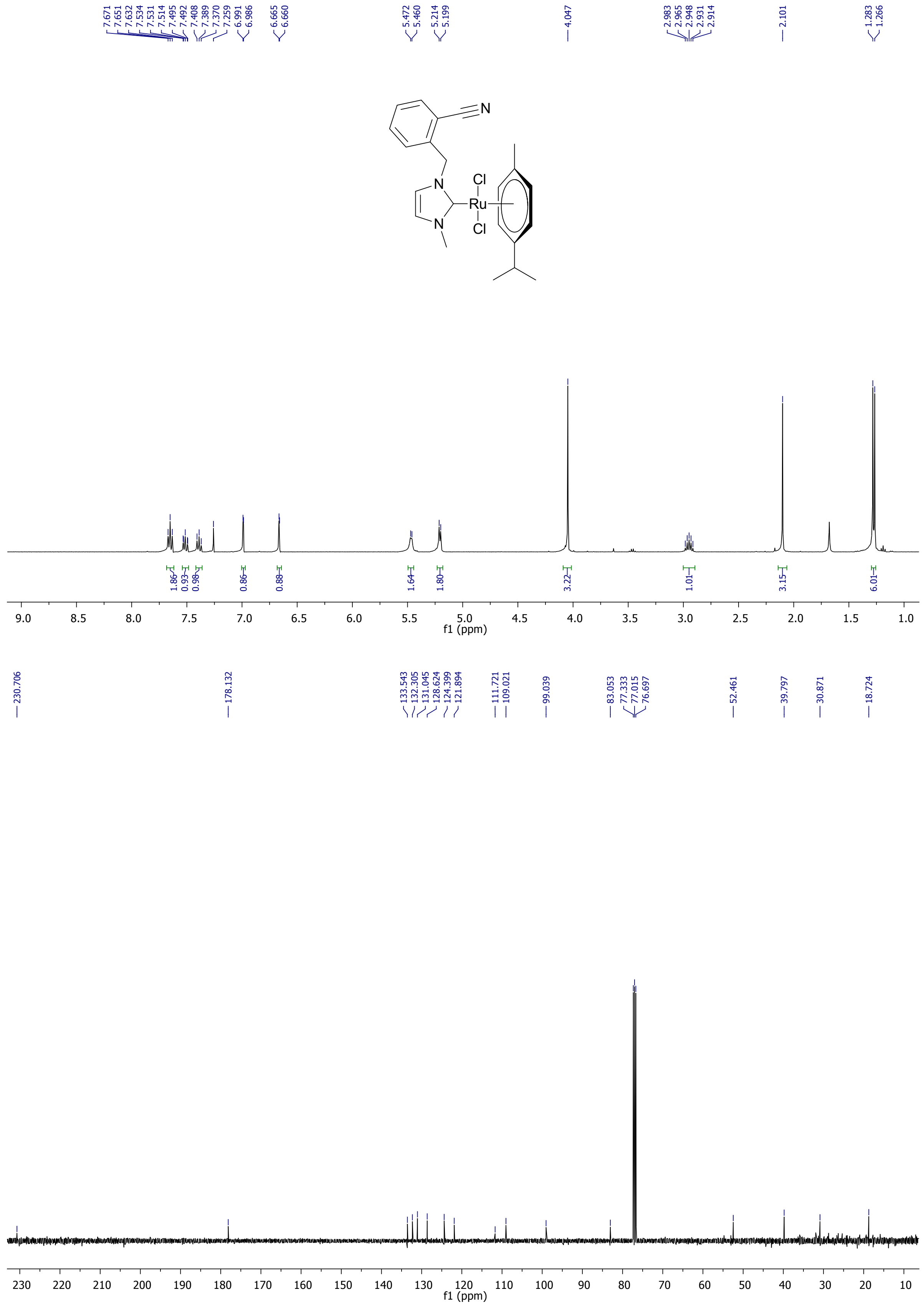

Figure S9. ^{1}H and ^{13}C NMR spectra of complex **3c** ($CDCl_3$).

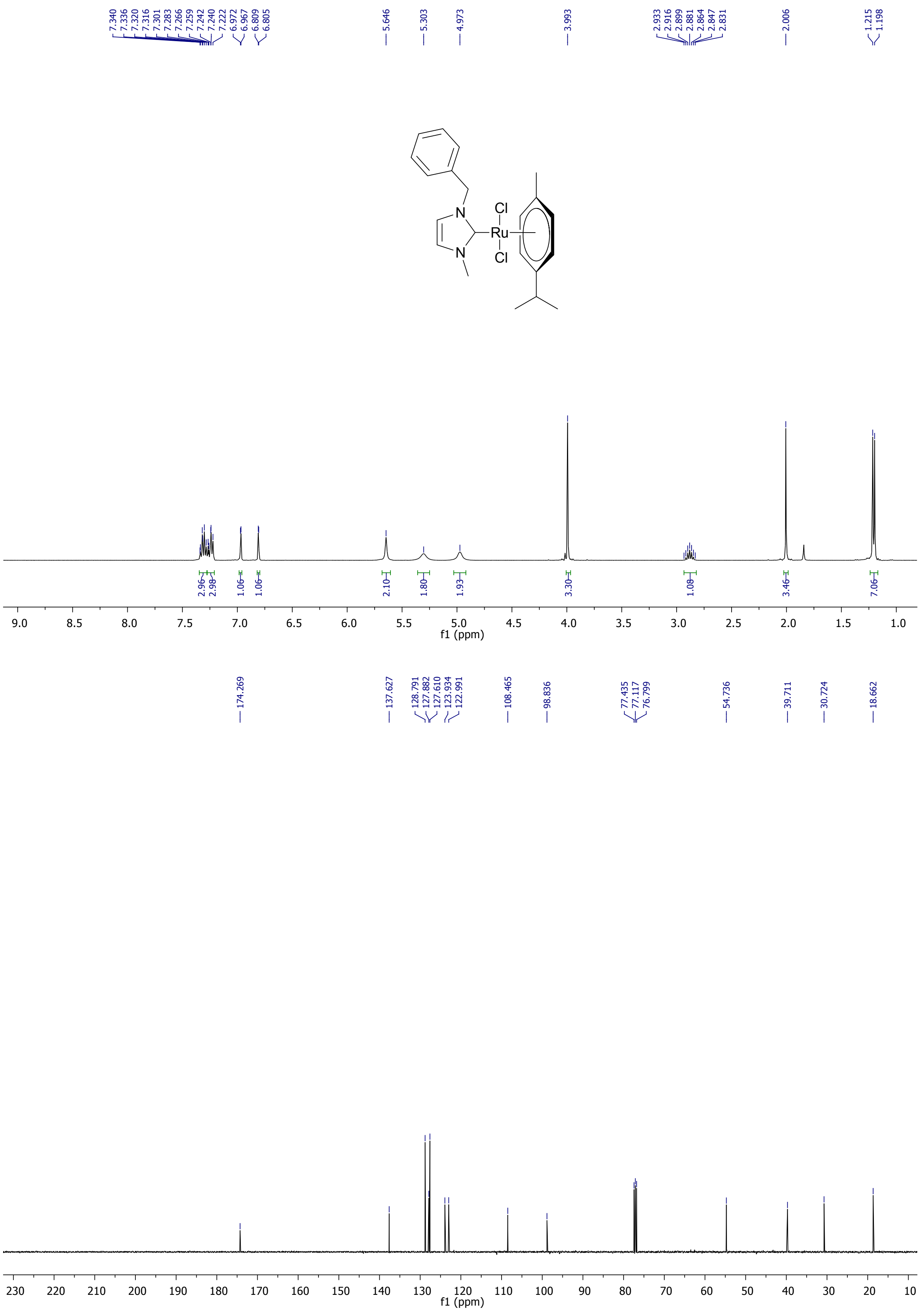

Figure S10. ^{1}H and ^{13}C NMR spectra of complex **3d** ($CDCl_3$).

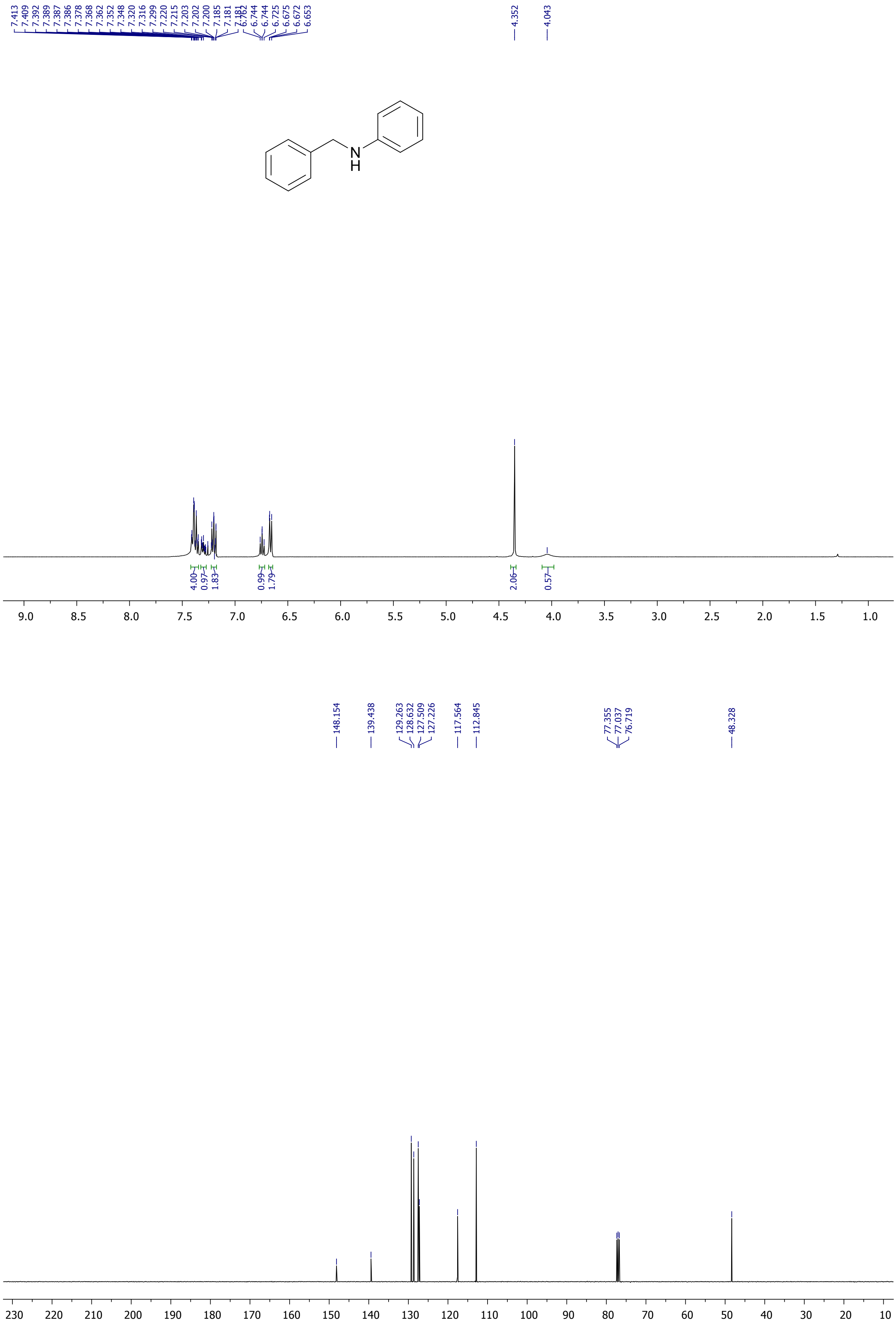

Figure S11. ^{1}H and ^{13}C NMR spectra of compound **12a** ($CDCl_3$).

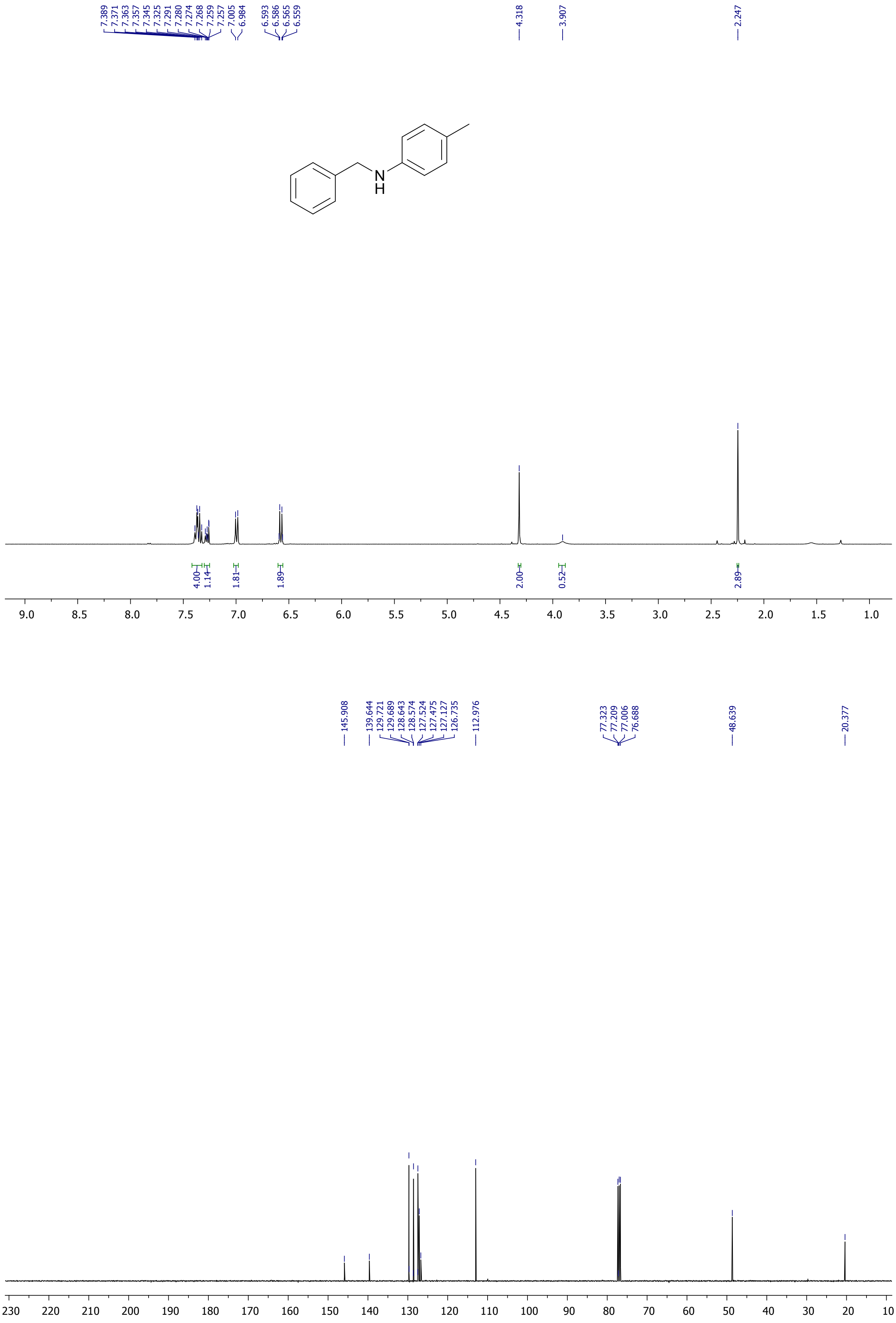

Figure S12. ^{1}H and ^{13}C NMR spectra of compound **12b** ($CDCl_3$).

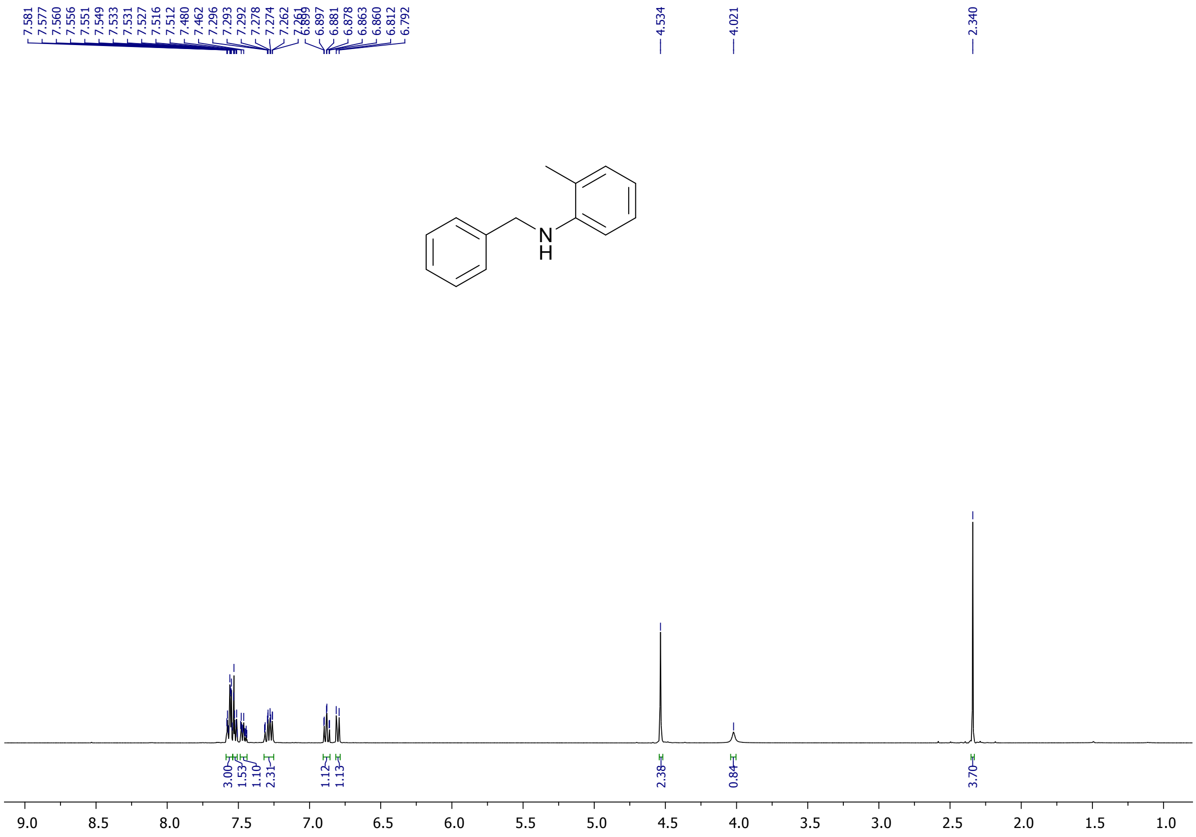

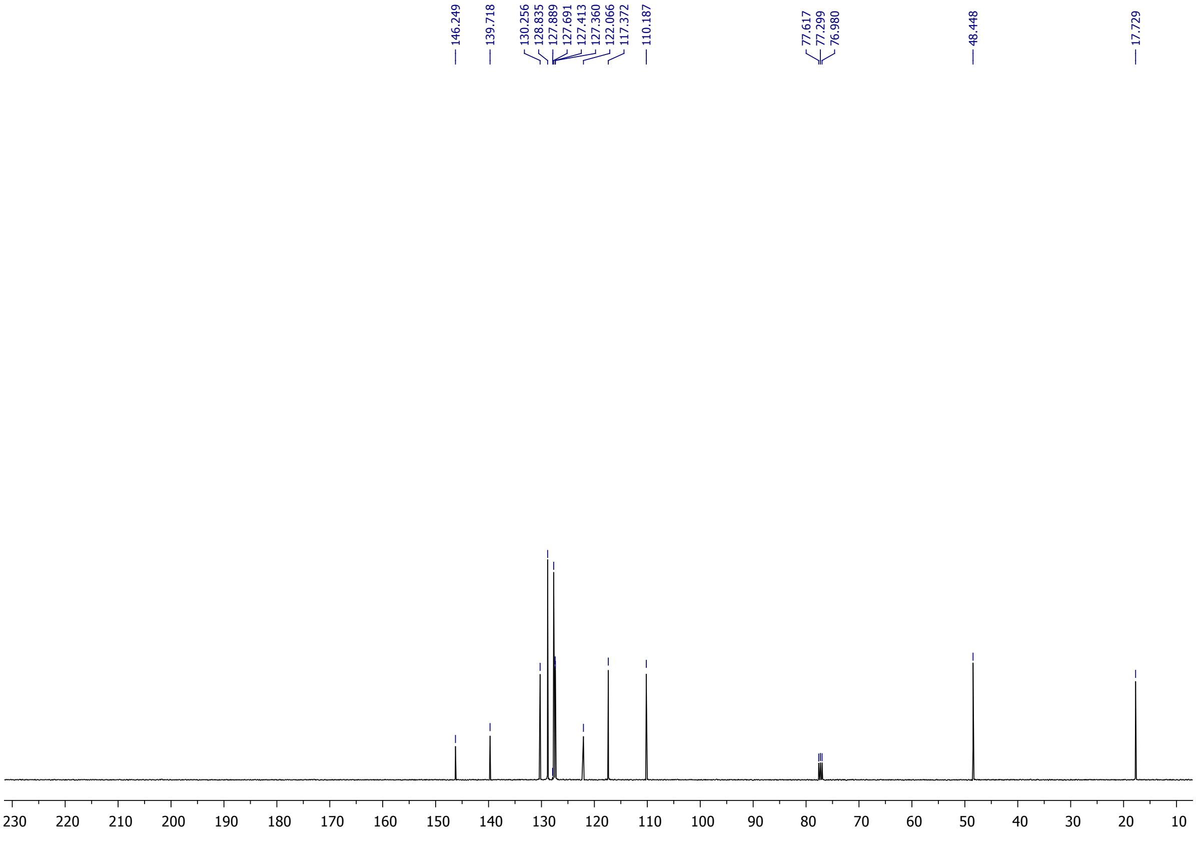

Figure S13. ^1H and ^{13}C NMR spectra of compound **12c** ($CDCl_3$).

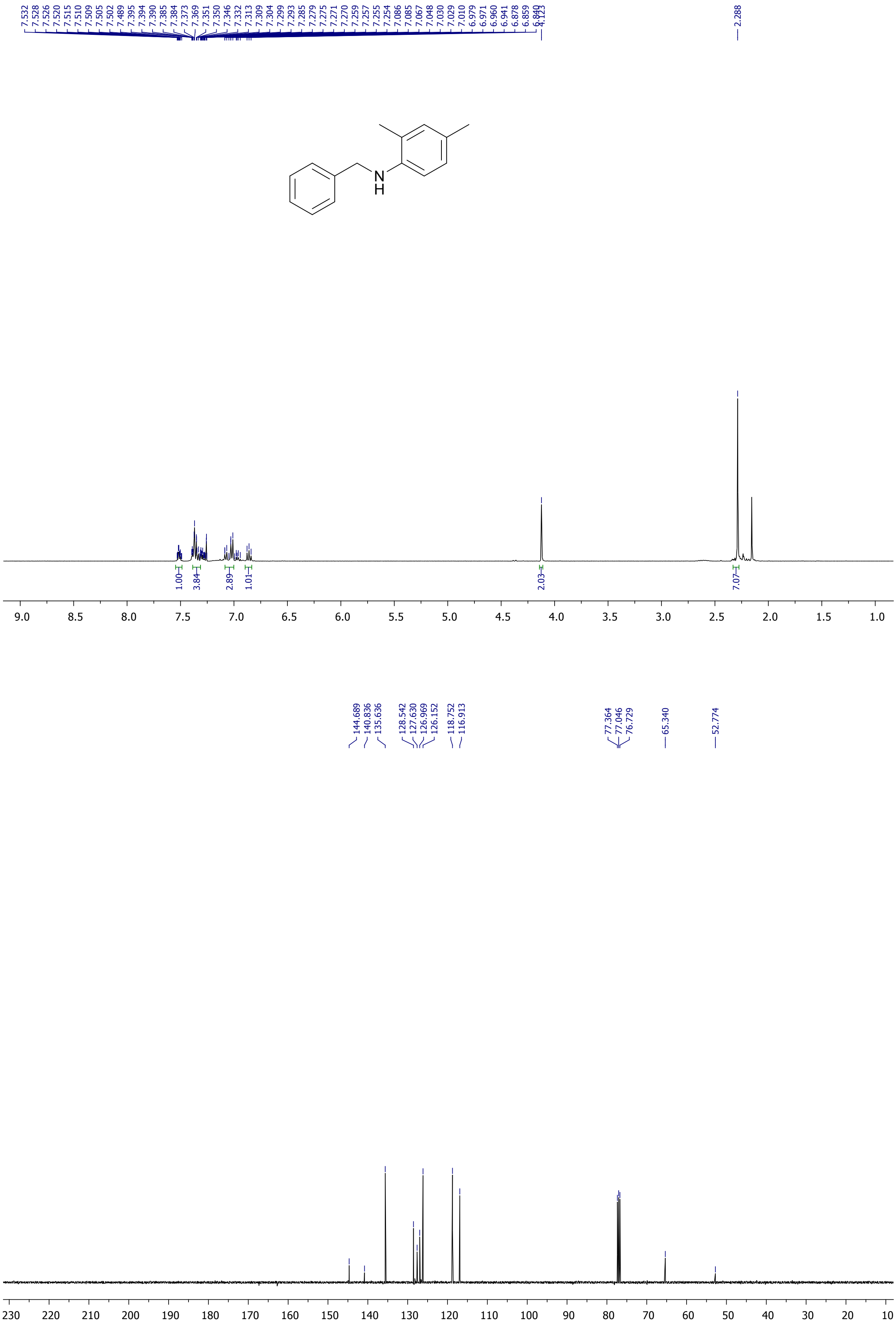

Figure S14. ^{1}H and ^{13}C NMR spectra of compound **12d** ($CDCl_3$).

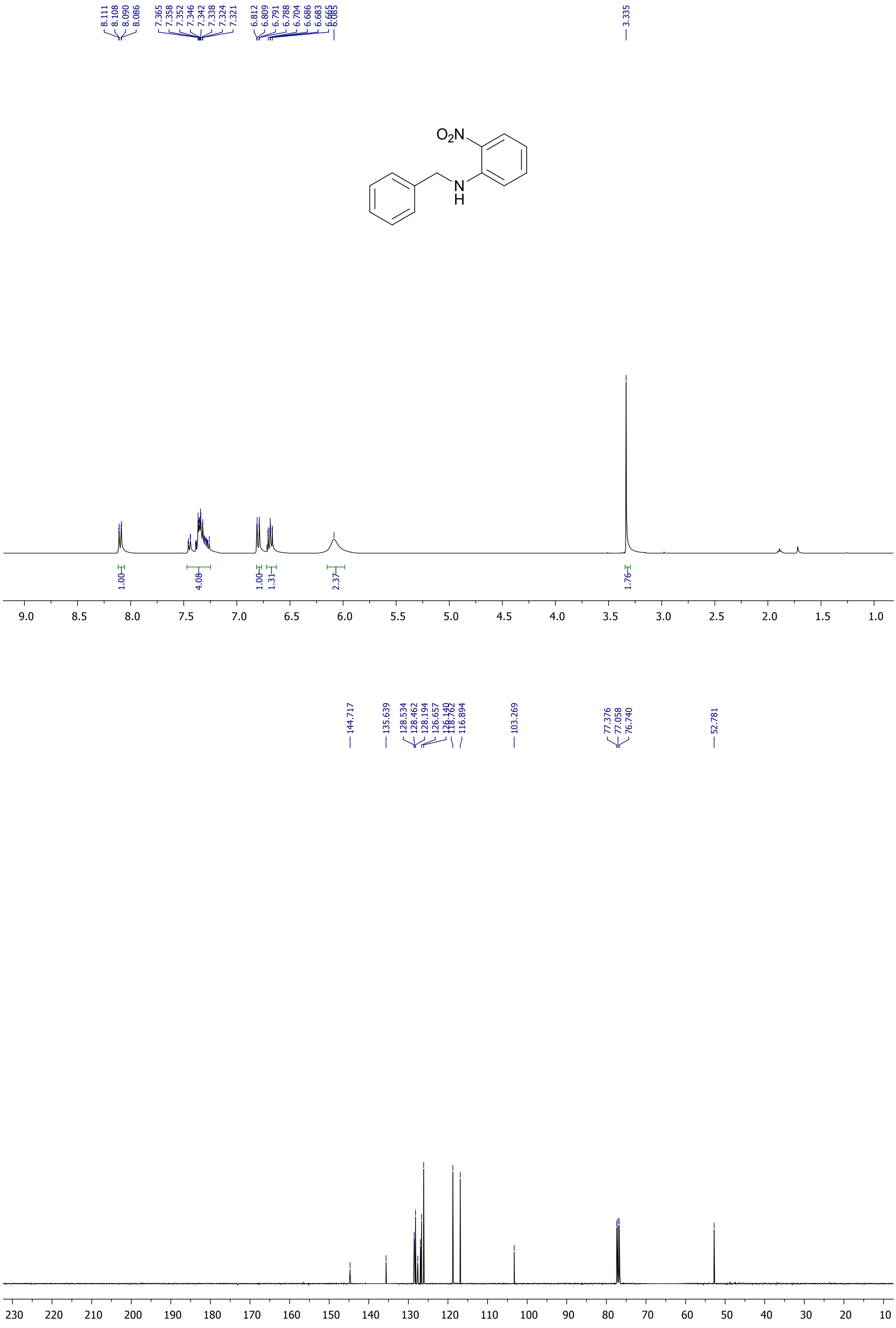

Figure S15. ^{1}H and ^{13}C NMR spectra of compound **12e** ($CDCl_3$).

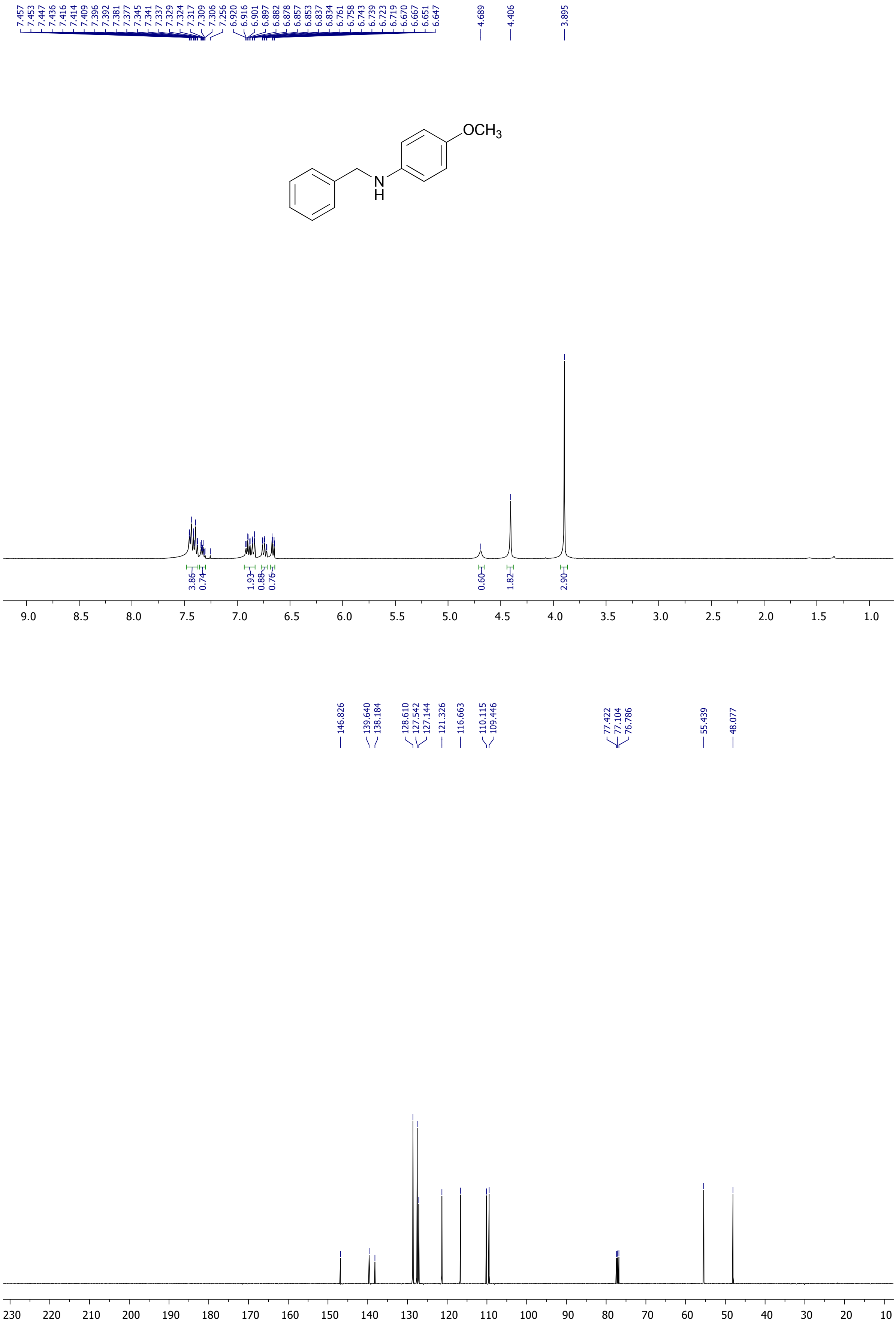

Figure S16. ^{1}H and ^{13}C NMR spectra of compound **12f** ($CDCl_3$).

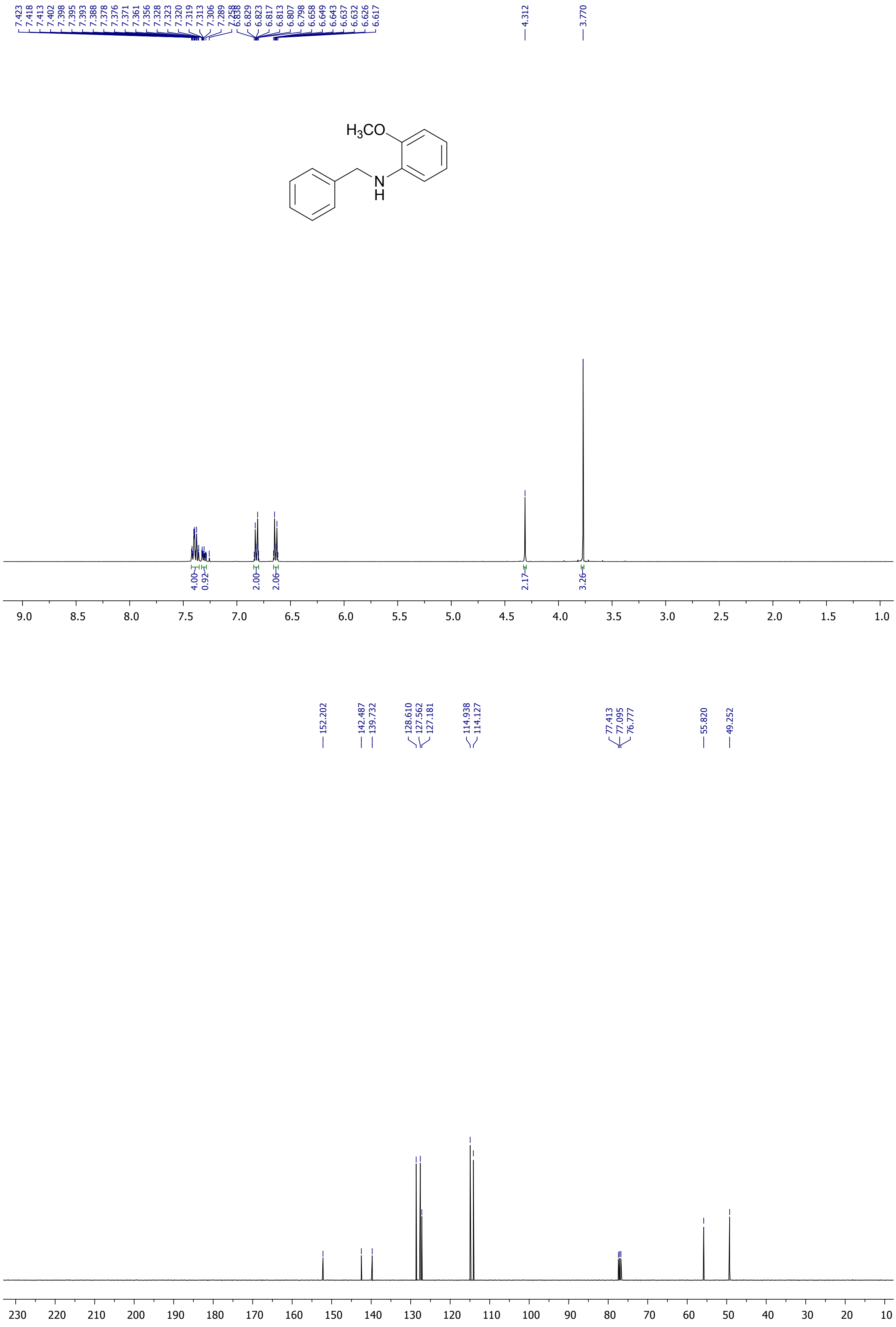

Figure S17. ^1H and ^{13}C NMR spectra of compound **12g** ($CDCl_3$).

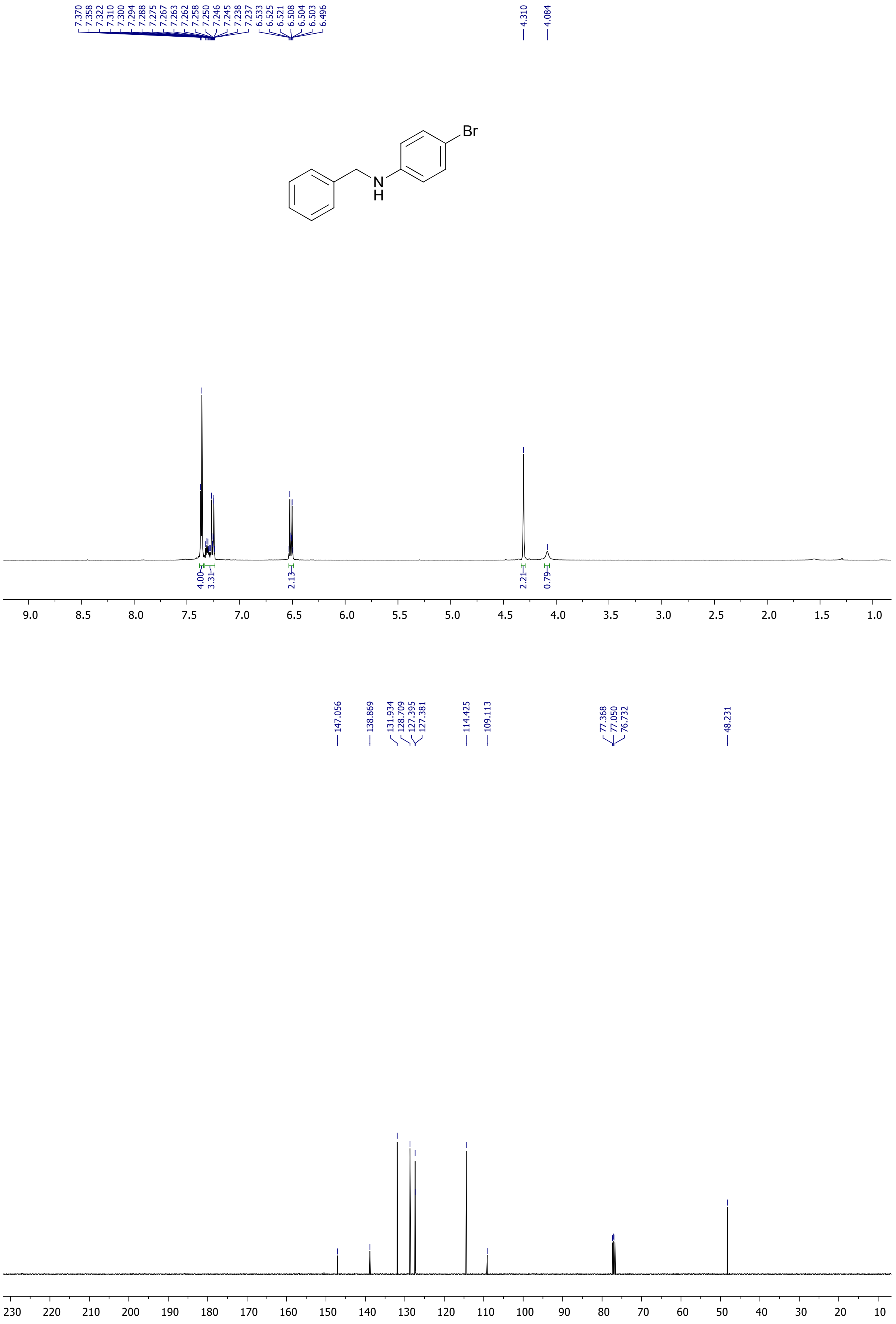

Figure S18. ^{1}H and ^{13}C NMR spectra of compound **12h** ($CDCl_3$).

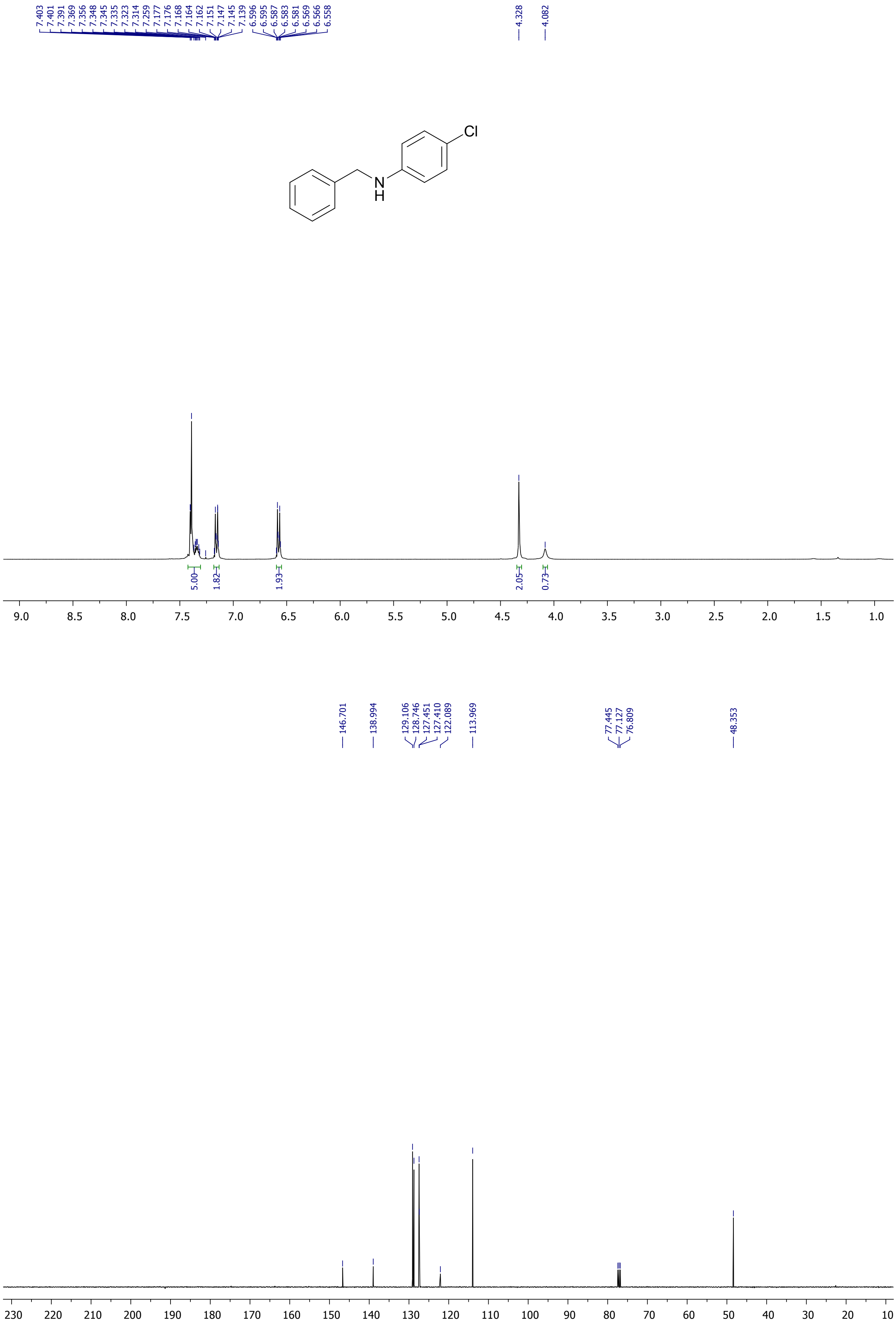

Figure S19. ^{1}H and ^{13}C NMR spectra of compound **12i** ($CDCl_3$).

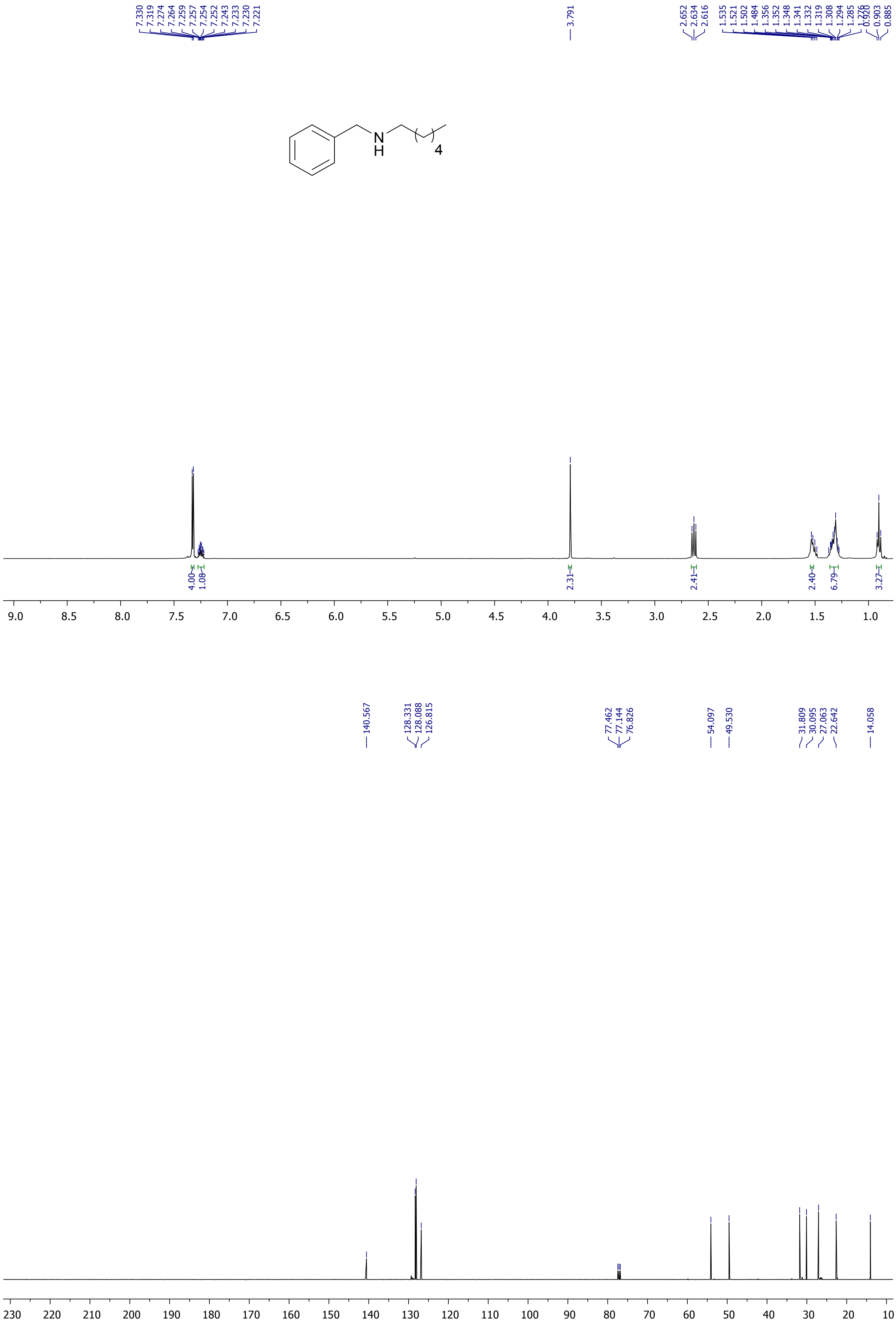

Figure S20. ^{1}H and ^{13}C NMR spectra of compound **12j** ($CDCl_3$).

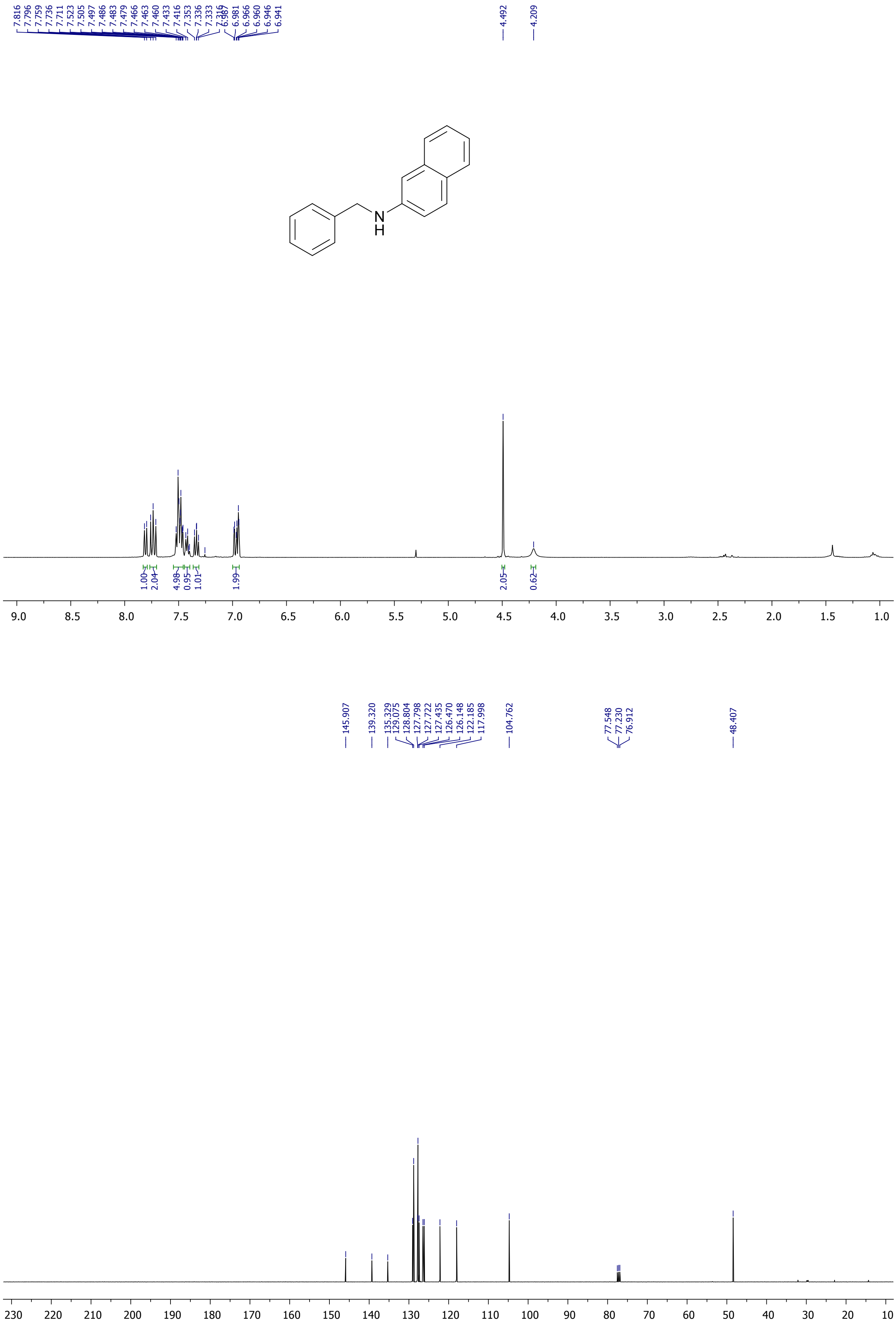

Figure S21. ^{1}H and ^{13}C NMR spectra of compound **12k** ($CDCl_3$).

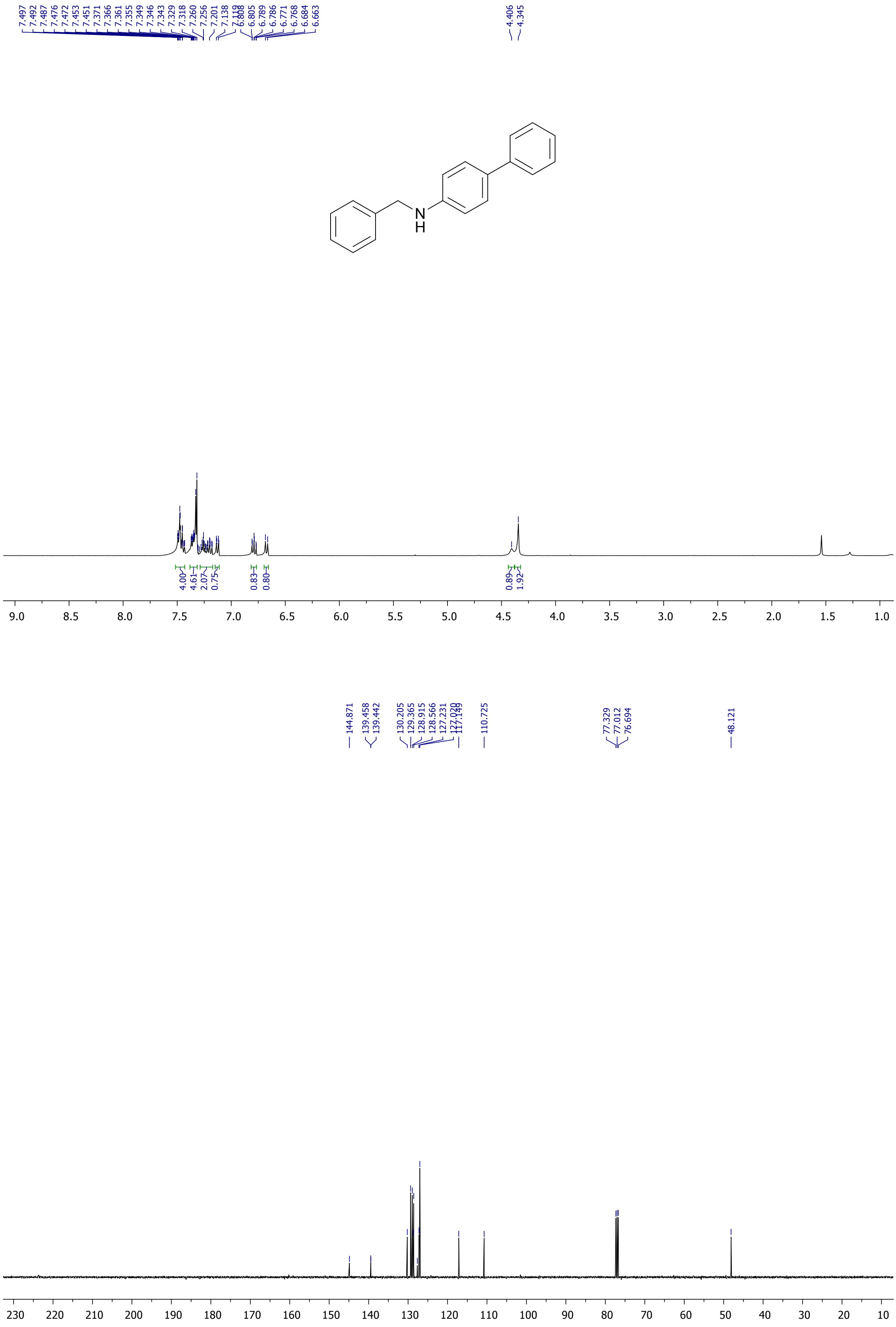

Figure S22. ^{1}H and ^{13}C NMR spectra of compound **12l** ($CDCl_3$).

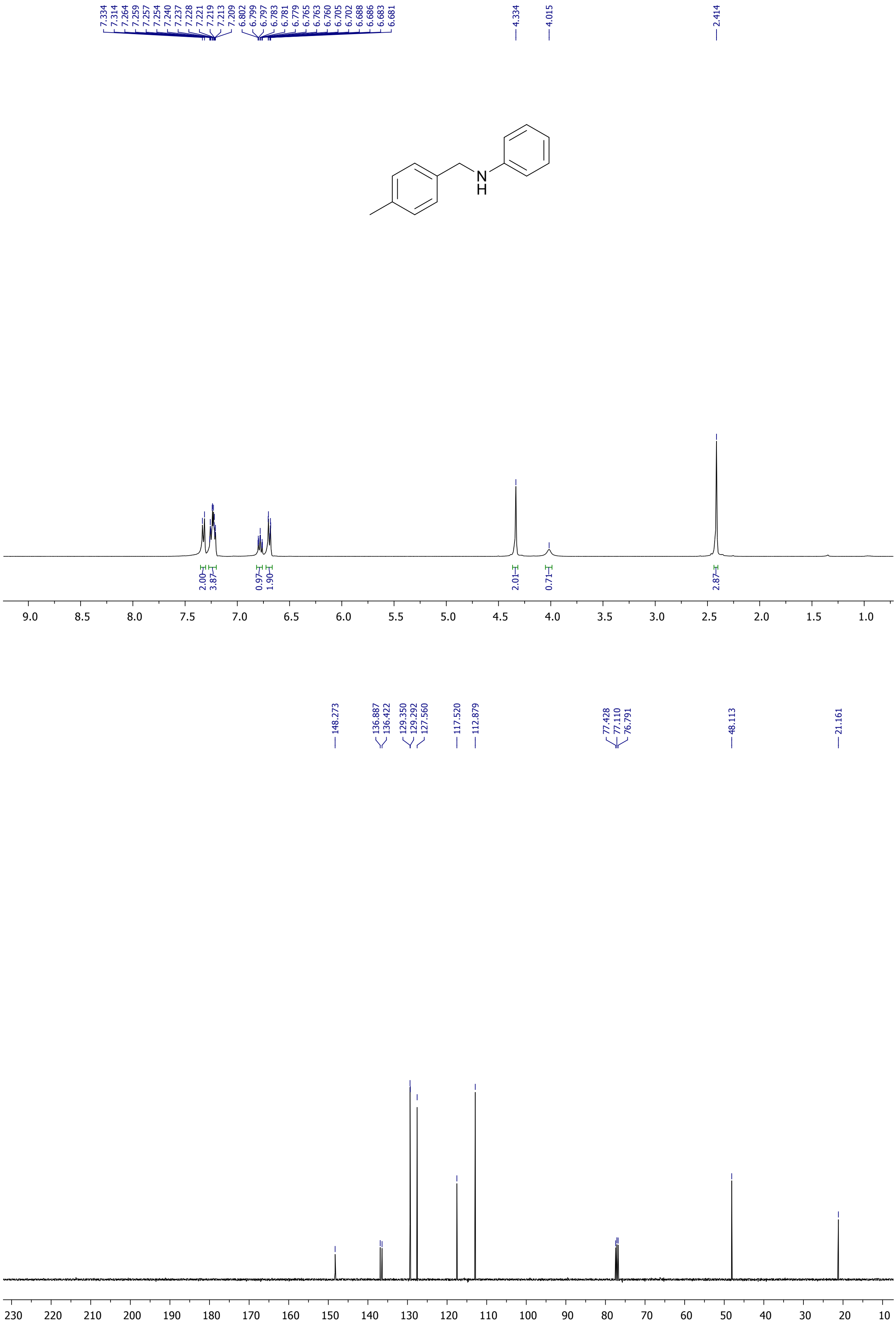

Figure S23. ^{1}H and ^{13}C NMR spectra of compound **13a** ($CDCl_3$).

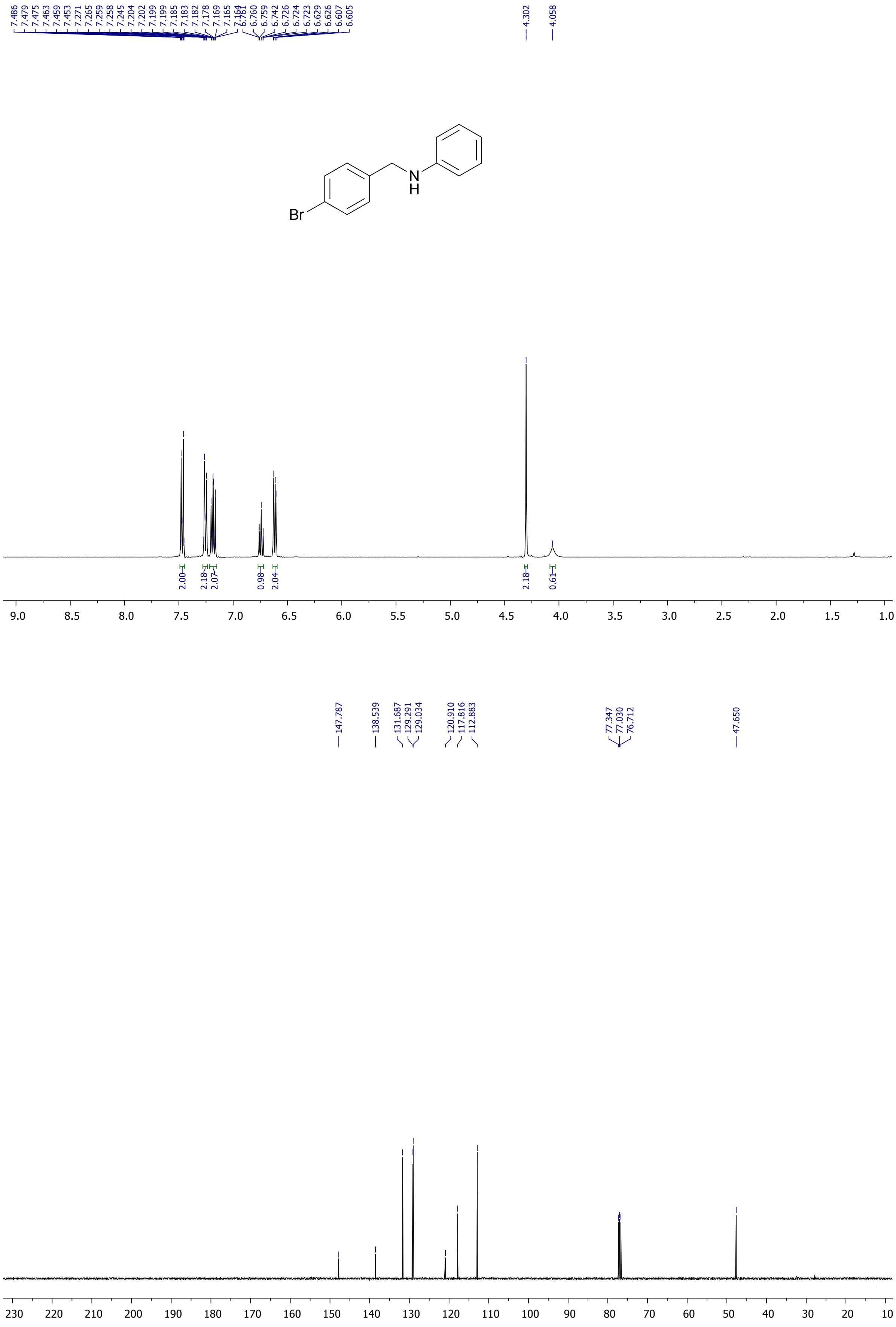

Figure S24. ^{1}H and ^{13}C NMR spectra of compound **13b** ($CDCl_3$).

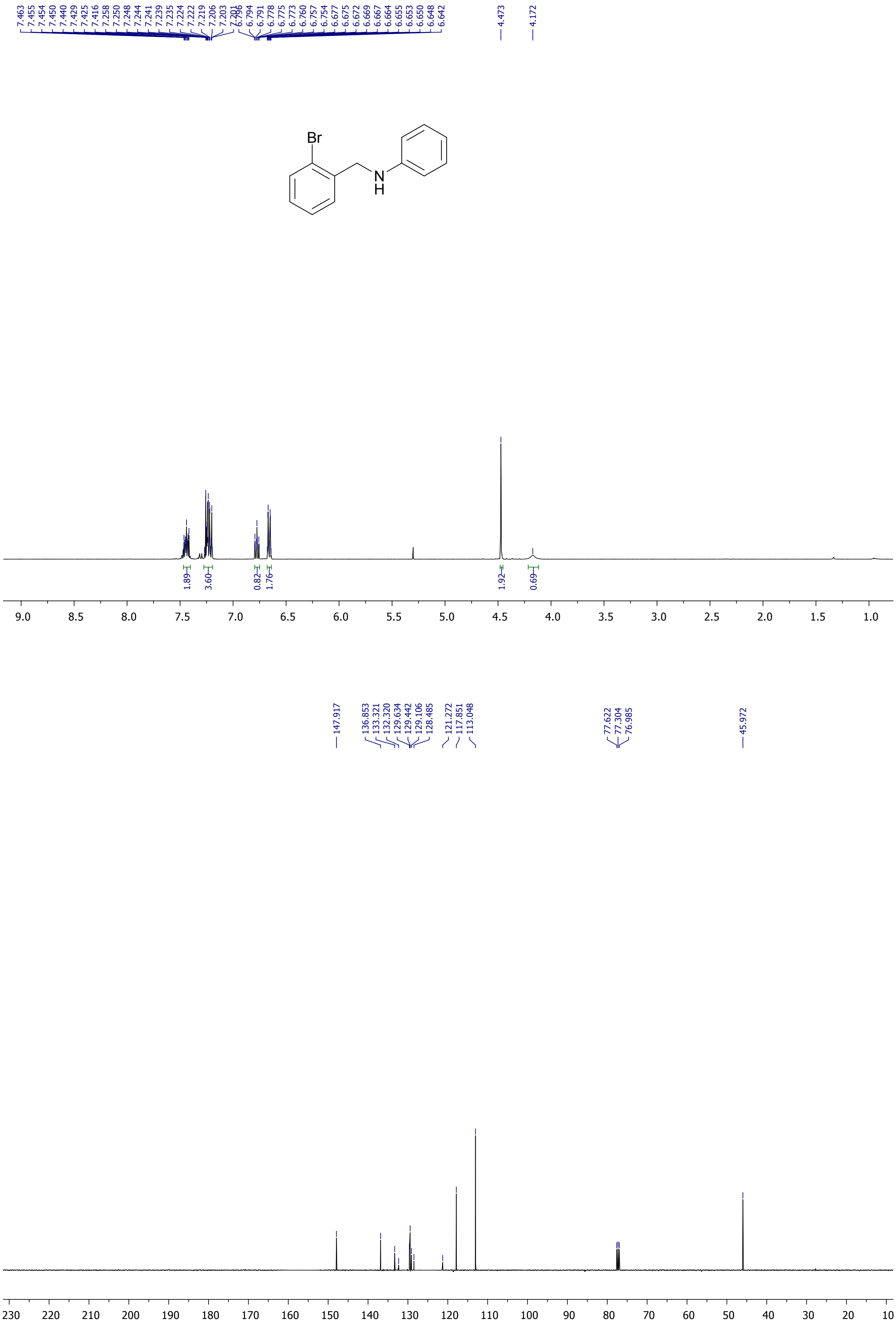

Figure S25. ^{1}H and ^{13}C NMR spectra of compound **13c** ($CDCl_3$).

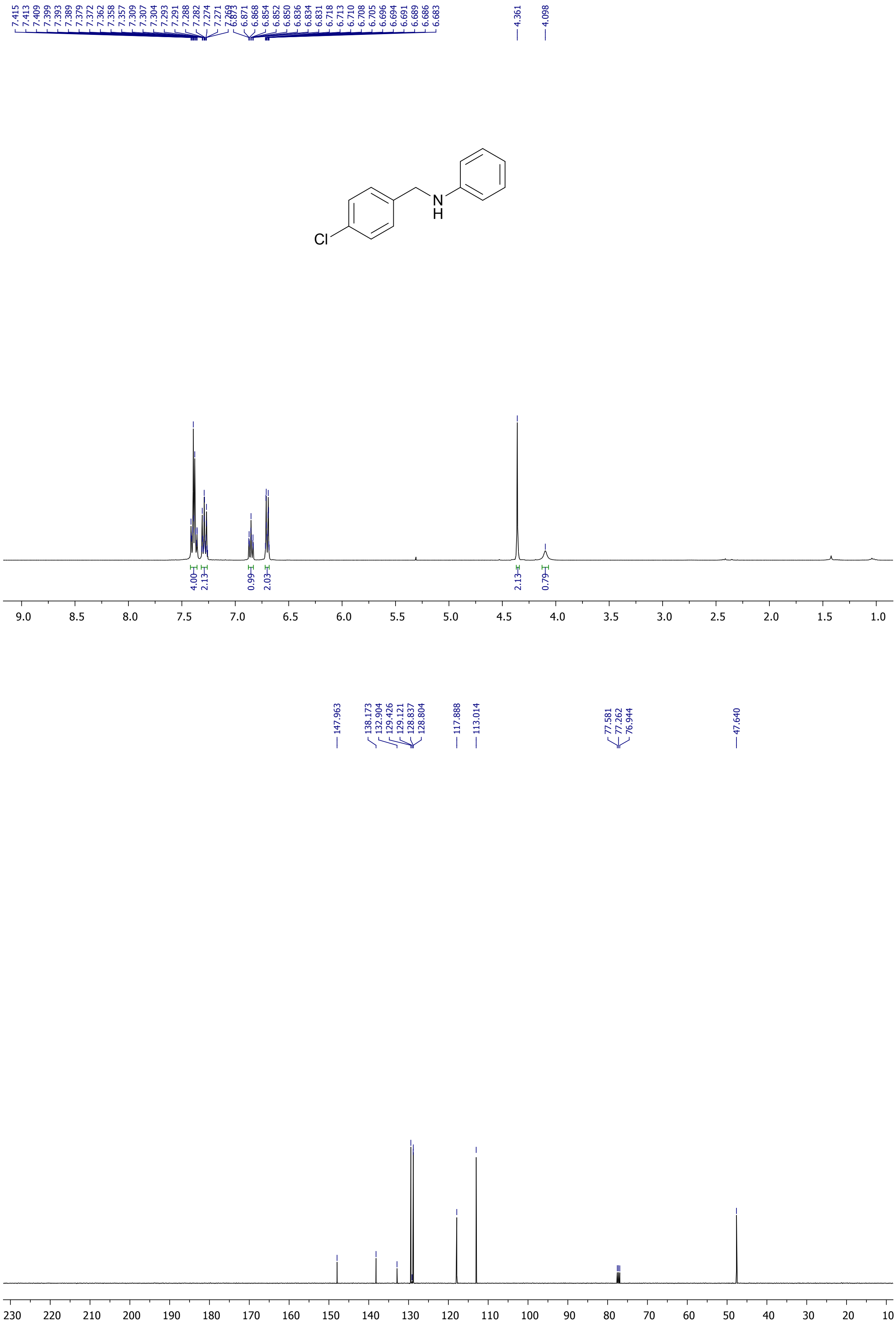

Figure S26. ^{1}H and ^{13}C NMR spectra of compound **13d** ($CDCl_3$).

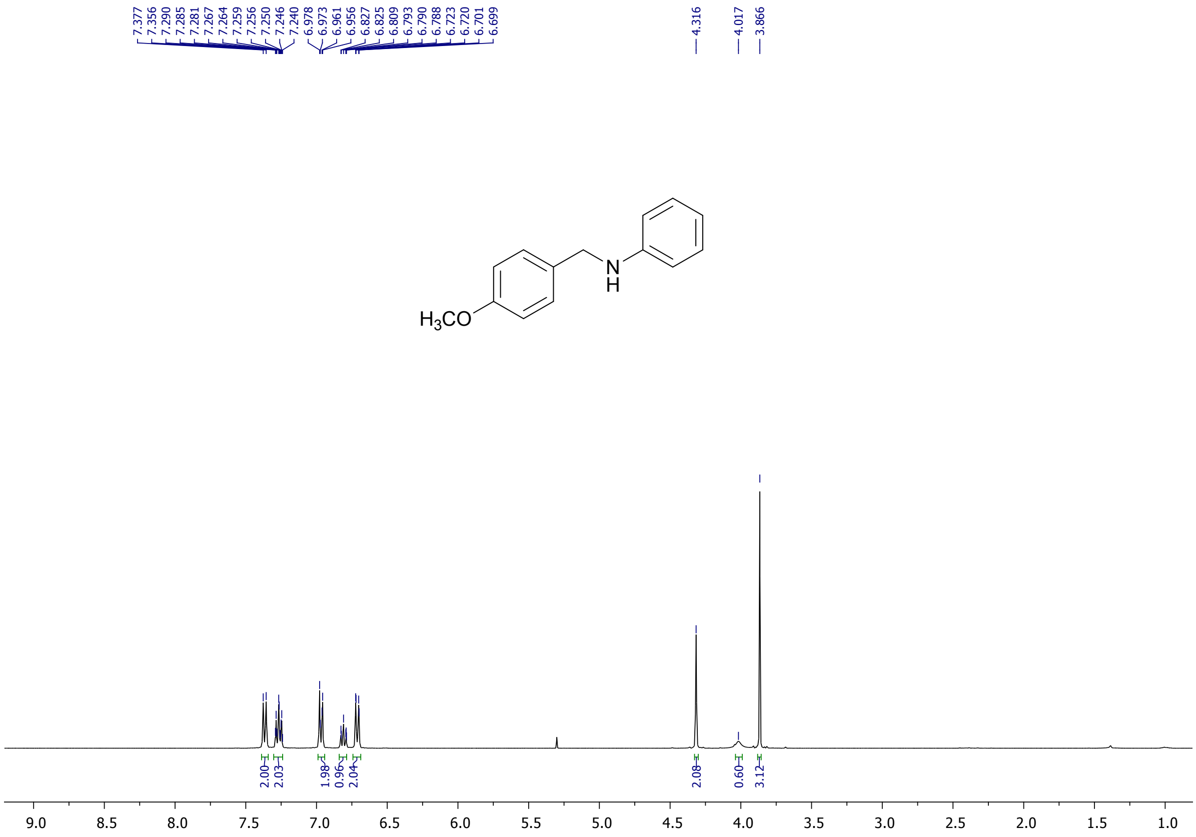

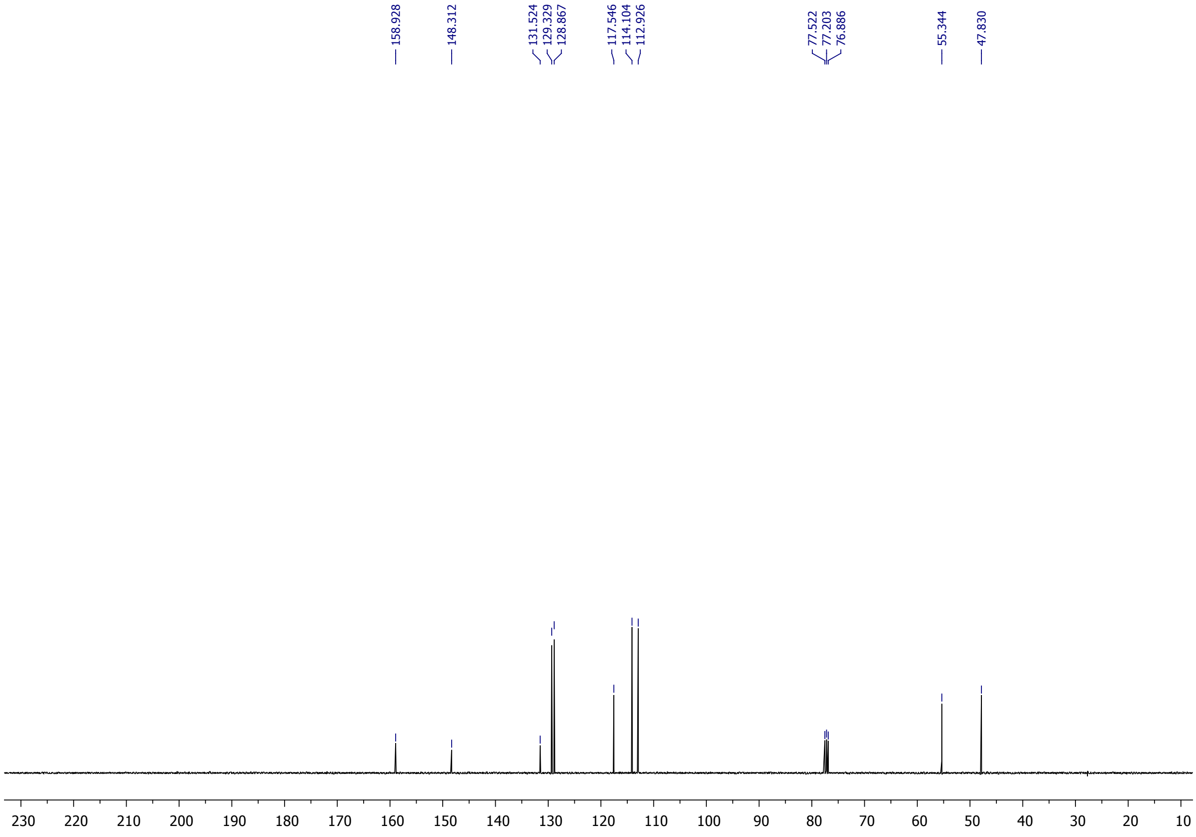

Figure S27. ^{1}H and ^{13}C NMR spectra of compound **13e** ($CDCl_3$).

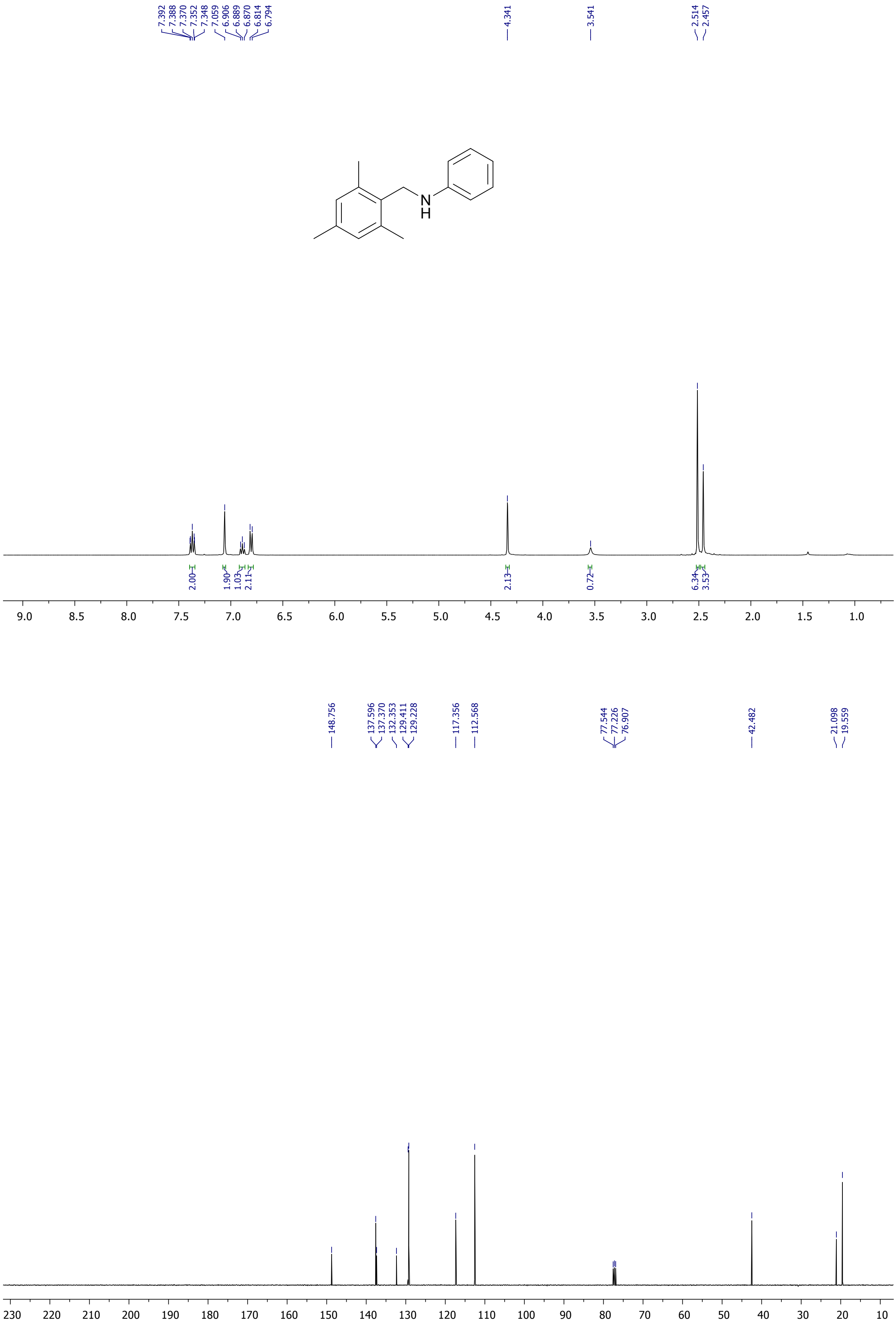

Figure S28. ^{1}H and ^{13}C NMR spectra of compound **13f** ($CDCl_3$).

Table S1 Elemental analysis results of the complexes **2a-d** and **3a-d**.

Sample	%C	%H	%N	%S
**2a**	**44.931**	**4.25.28**	**3.9534**	**2.7159**
**2b**	**46.466**	**4.8401**	**5.8835**	**-**
**2c**	**46.457**	**5.2603**	**6.2050**	**-**
**2d**	**42.032**	**4.9800**	**4.7528**	**-**
**3a**	**52.585**	**4.8610**	**4.4582**	**4.1602**
**3b**	**54.764**	**5.3754**	**7.2315**	**-**
**3c**	**49.137**	**5.2781**	**8.0532**	**-**
**3d**	**48.832**	**5.8370**	**5.6175**	

FTIR Results

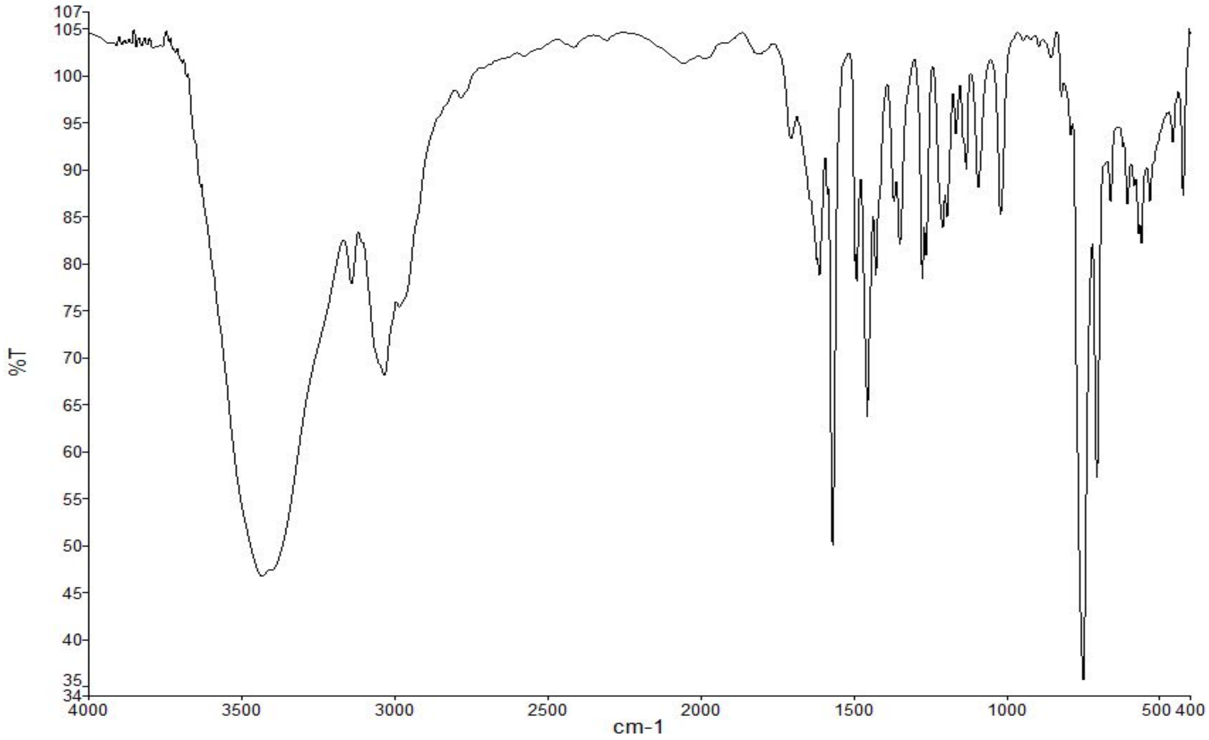

Figure S29. IR spectrum of **1e**.

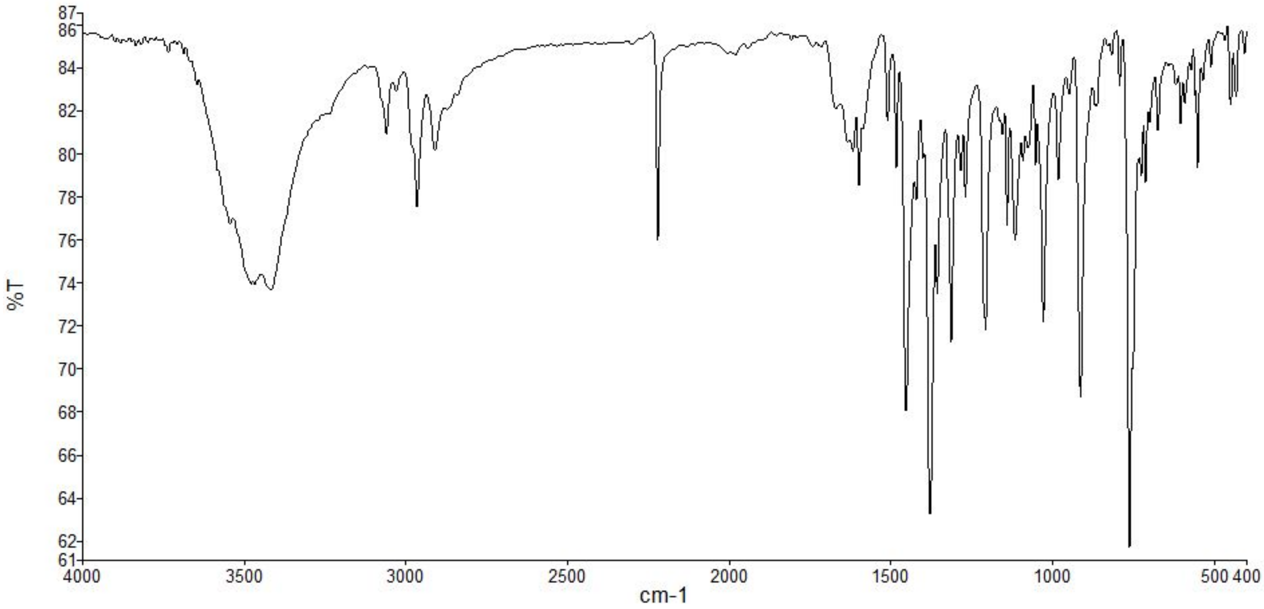

Figure S30. IR spectrum of **2a**.

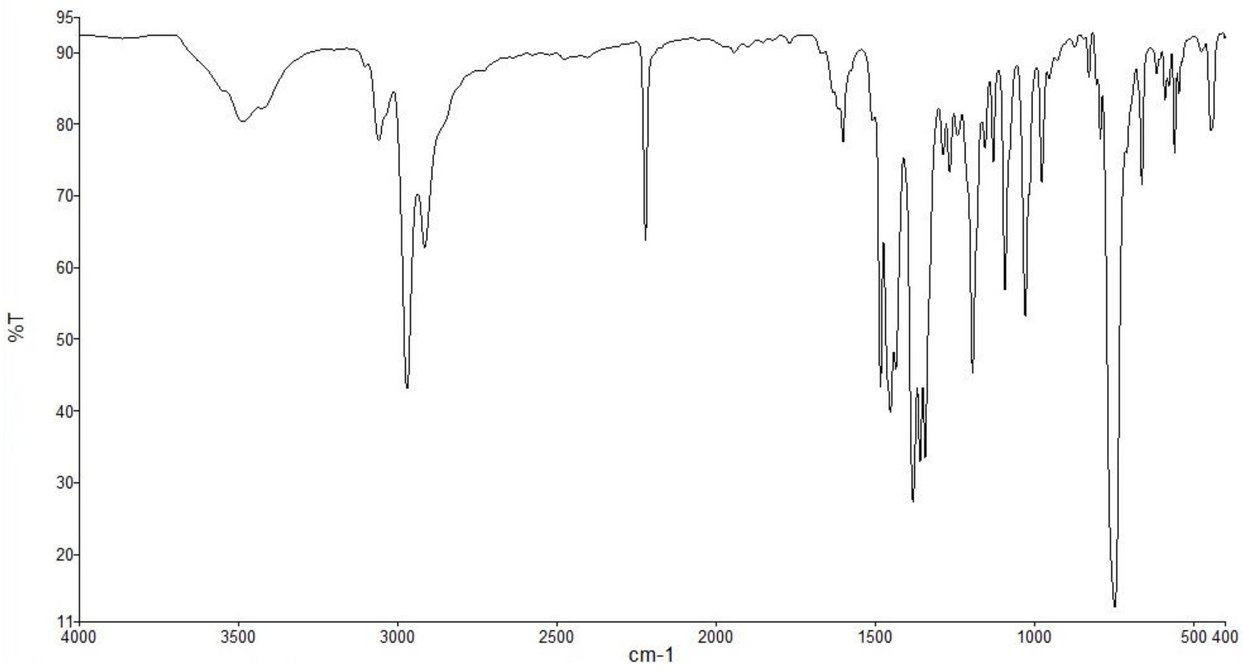

Figure S31. IR spectrum of **2b**.

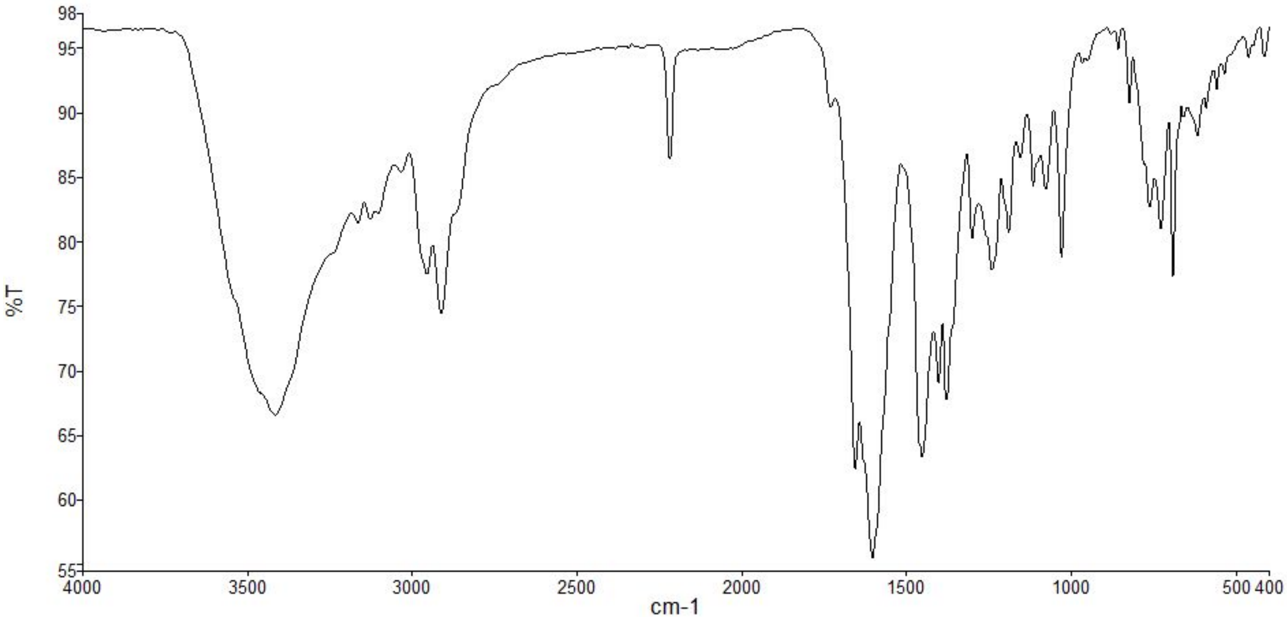

Figure S32. IR spectrum of **2c**.

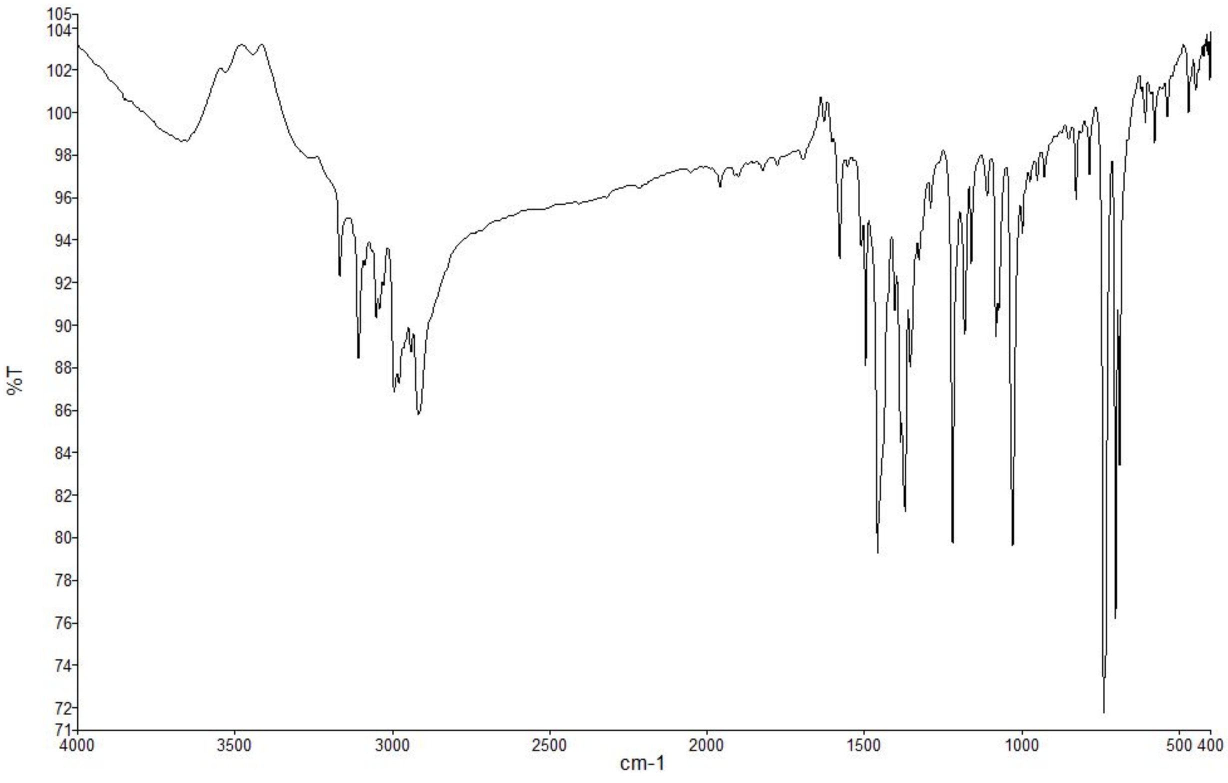

Figure S33. IR spectrum of **2d**.

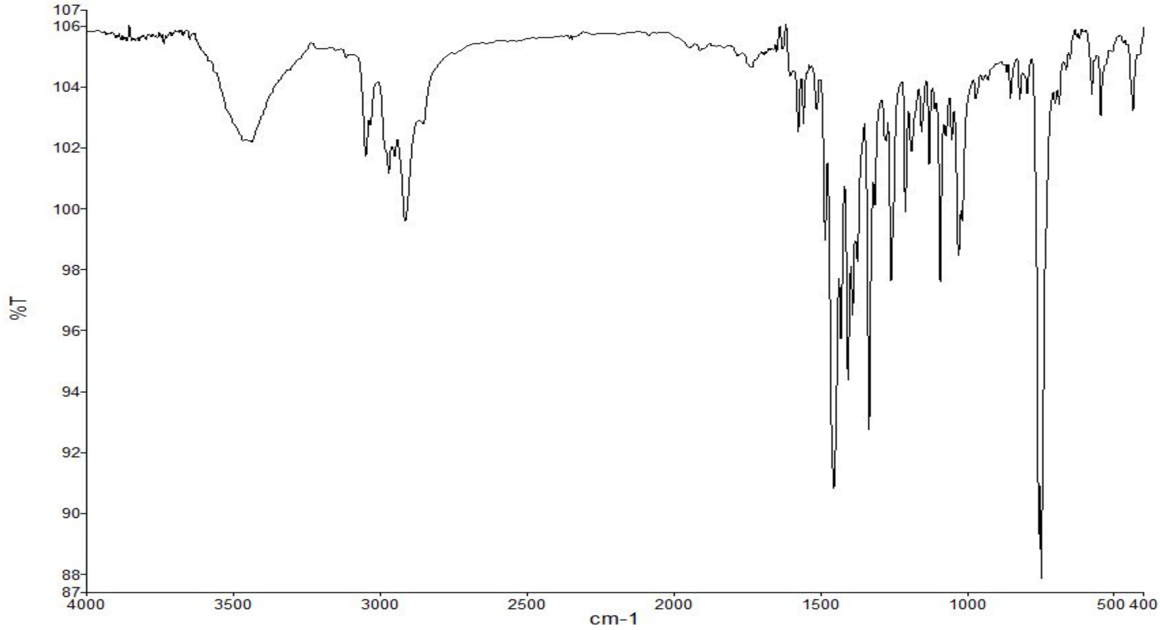

Figure S34. IR spectrum of **2e**.

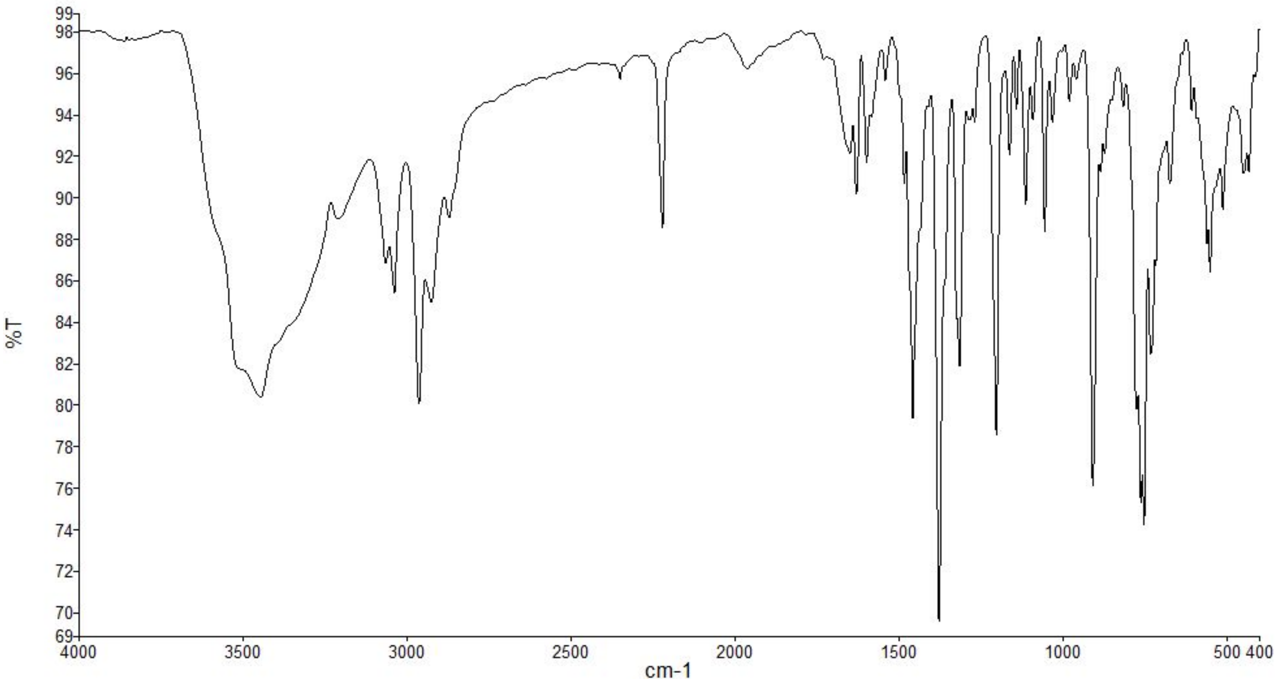

Figure S35. IR spectrum of **3a**.

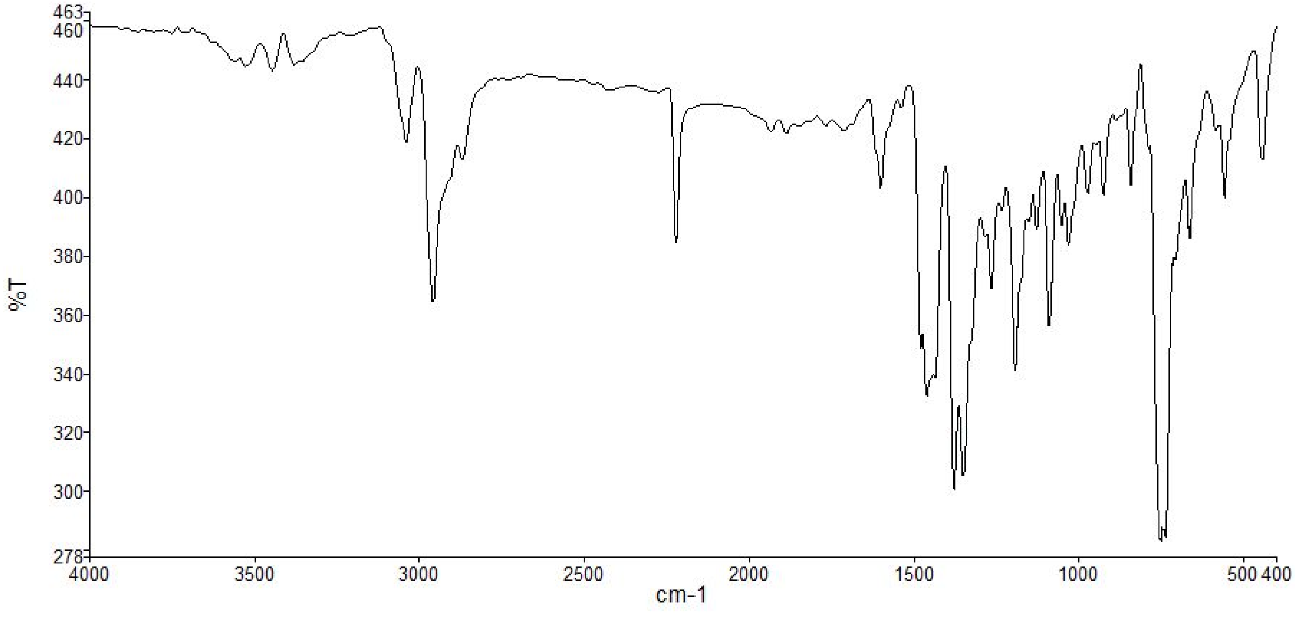

Figure S36. IR spectrum of **3b**.

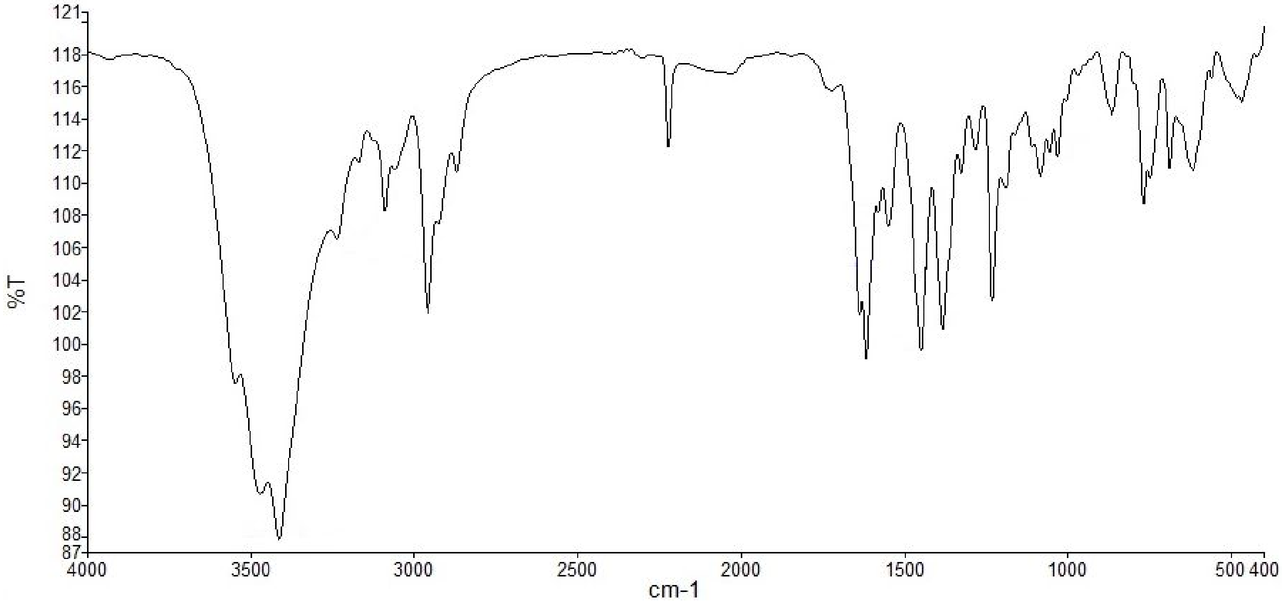

Figure S37. IR spectrum of **3c**.

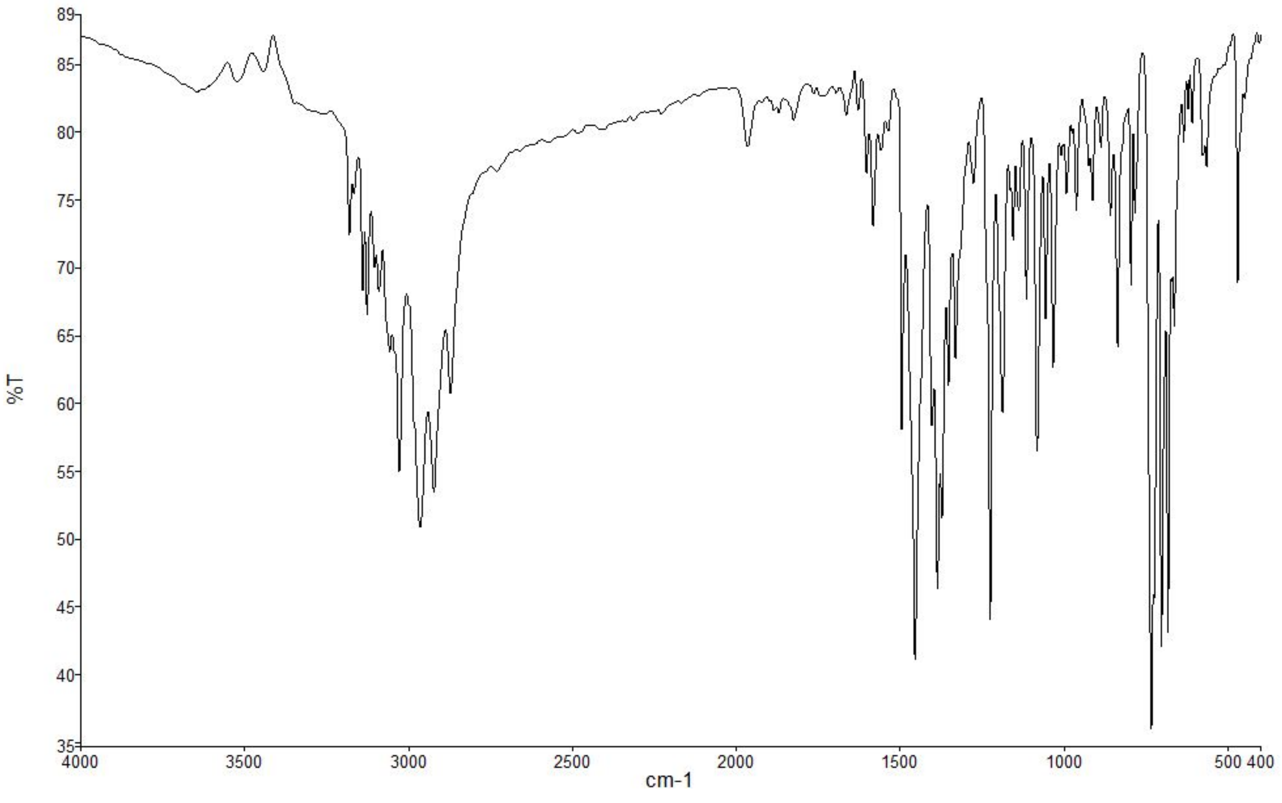

Figure S38. IR spectrum of **3d**.

Table S2 Crystal data and structure refinement parameters for complexes **2b, 3a** and **3c**.

	**2b**	**3a**	**3c**
Empirical formula	$C_{27}H_{35}Cl_5N_3IrO_3$	$C_{25}H_{24}Cl_2N_2RuS$	$C_{22}H_{25}Cl_2N_3Ru$
Formula weight	819.03	556.49	503.42
Crystal system	Triclinic	Triclinic	Monoclinic
Space group	P-1	P-1	$P2_1/n$
*a (*Å)	10.455 (7)	10.5955 (12)	15.852 (3)
*b (*Å)	11.712 (8)	15.2233 (16)	7.8121 (14)
*c (*Å)	16.887 (11)	18.699 (2)	17.689 (3)
*α (*º)	103.706 (12)	97.474 (5)	90.00
β (º)	92.694 (9)°	100.453 (5)	96.036 (6)°
*γ (*º)	110.322 (16)	108.332 (4)	90.00
*V (*Å^3)	1865 (2)	2758.4 (5)	2178.4 (7)
Z	2	4	4
D_c (g cm^{-3})	1.459	1.340	1.535
θ range (º)	2.3-26.6	2.9-26.9	2.9-25.9
Measured refls.	39509	71148	60019
Independent refls.	6911	10142	5459
R_{int}	0.125	0.083	0.053
S	1.05	1.26	1.09
R1/wR2	0.109/0.242	0.100/0.228	0.043/0.119
$\Delta\rho_{max}/\Delta\rho_{min}$ (eÅ^{-3})	1.91/-2.34	1.91/-1.67	0.79/-0.85

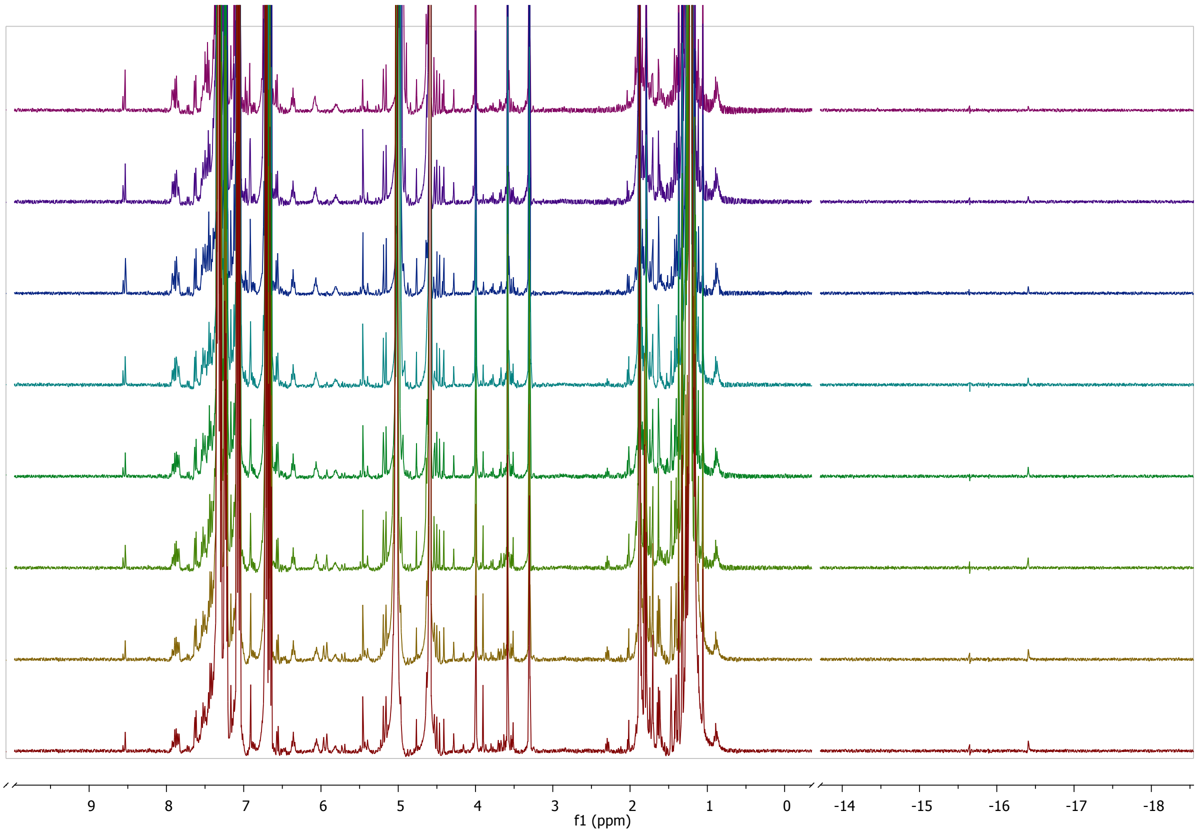

Figure S39. ^{1}H-NMR monitoring of *N*-alkylation of aniline with benzyl alcohol (From beginning to 24h).

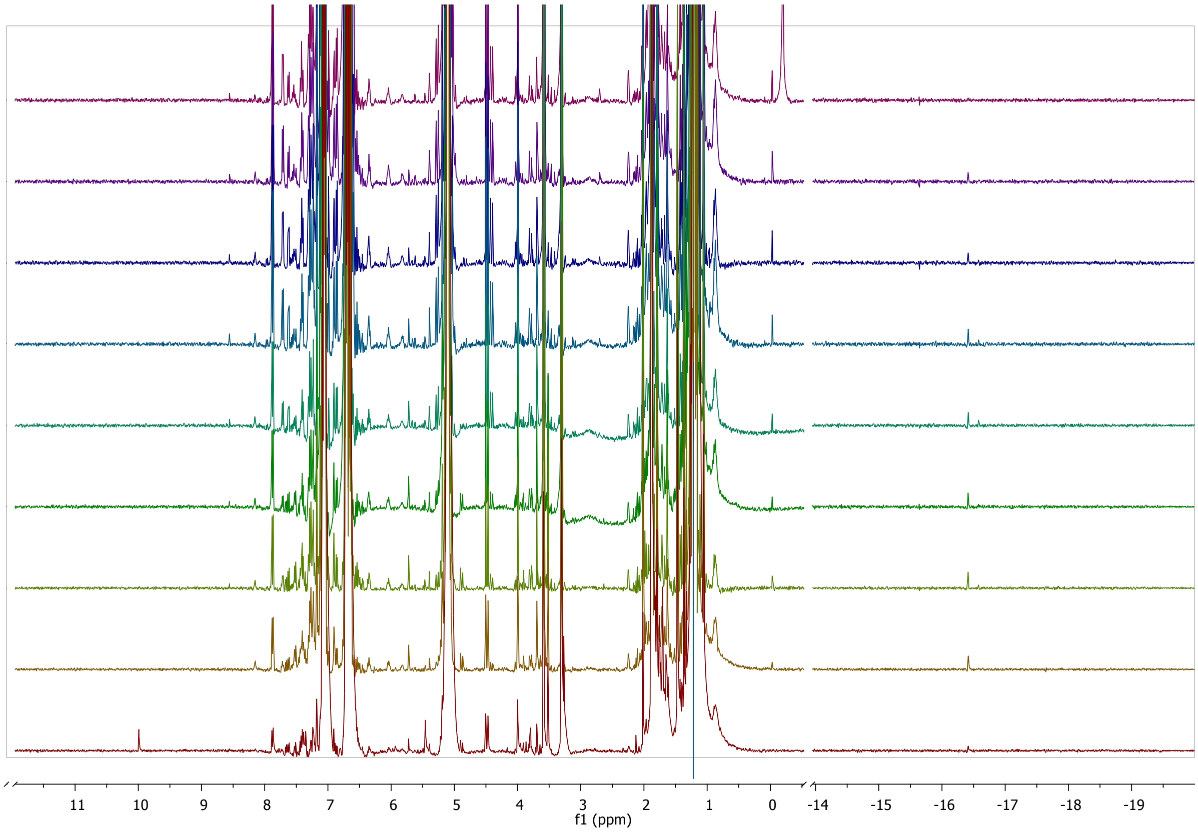

Figure S40. ^{1}H-NMR monitoring of *N*-methylation of aniline with methanol (From beginning to 24h).

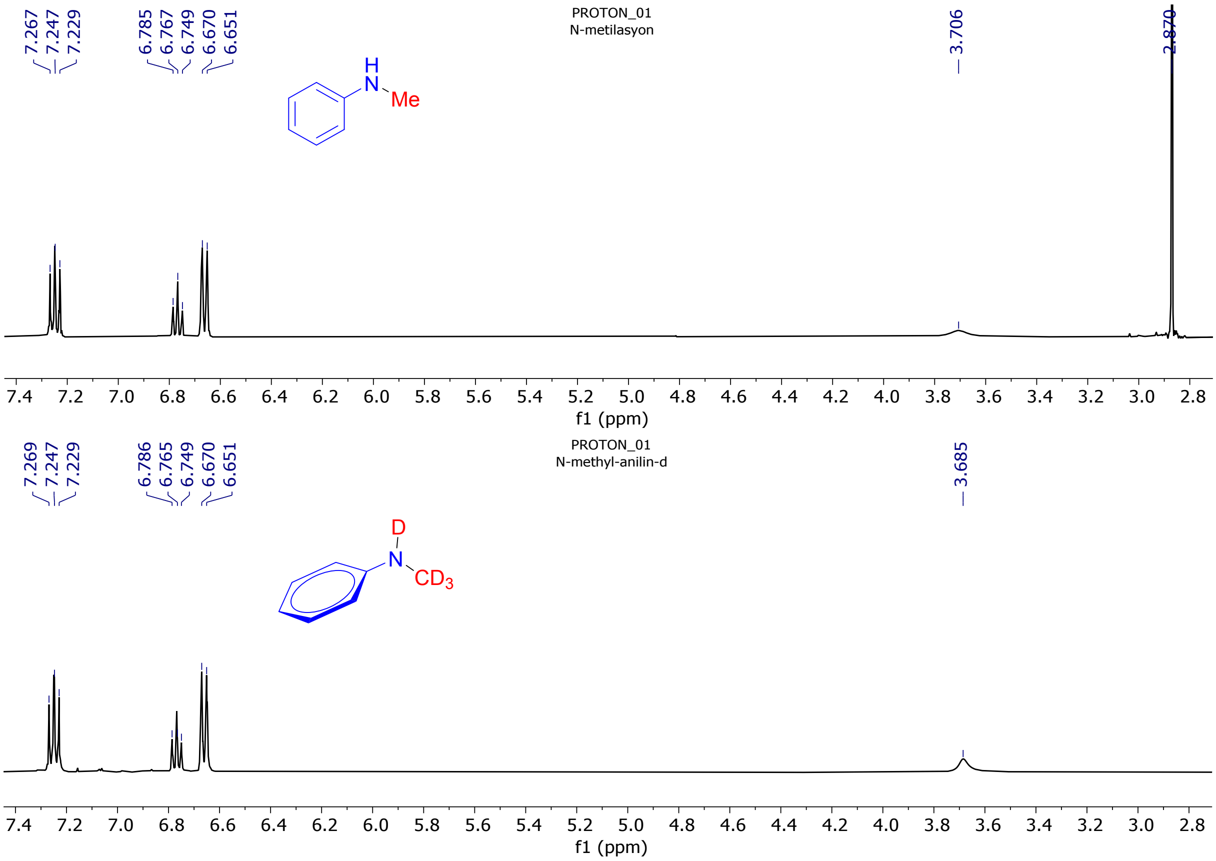

Figure S41. ^{1}H-NMR spectra of *N*-methylaniline (top) and *N*-(methyl-d_3)aniline-*d* (bottom).

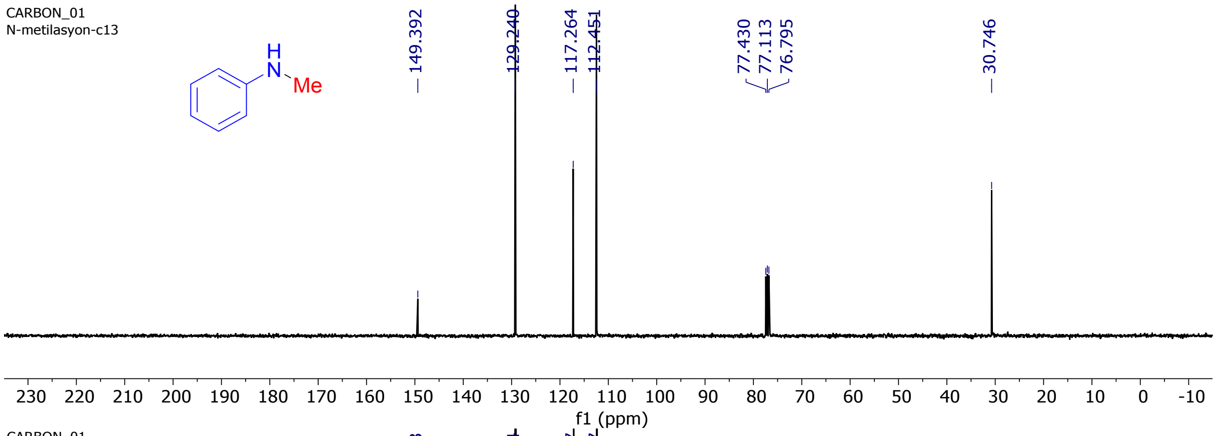

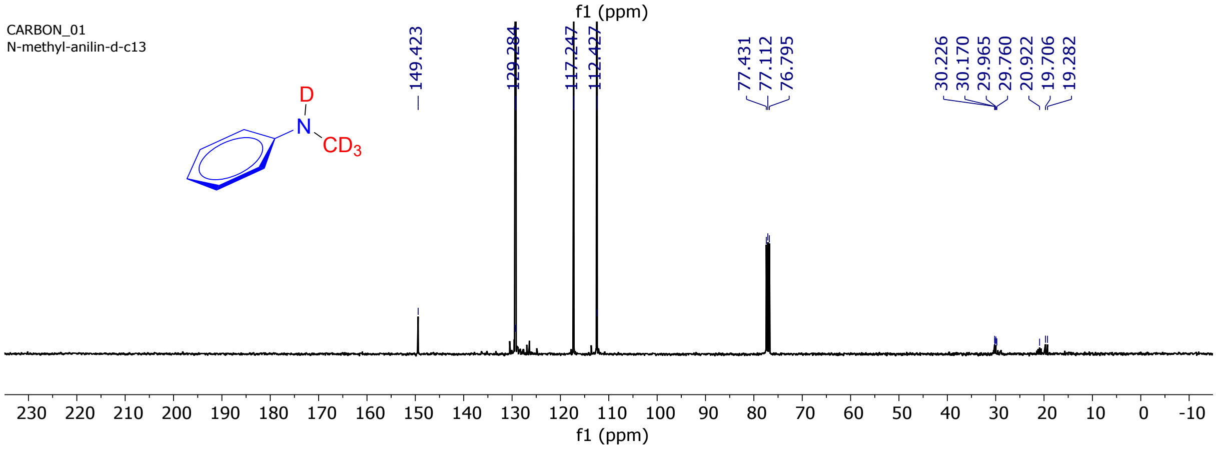

Figure S42. ^{13}C-NMR spectra of *N*-methylaniline (top) and *N*-(methyl-d_3)aniline-*d* (bottom).

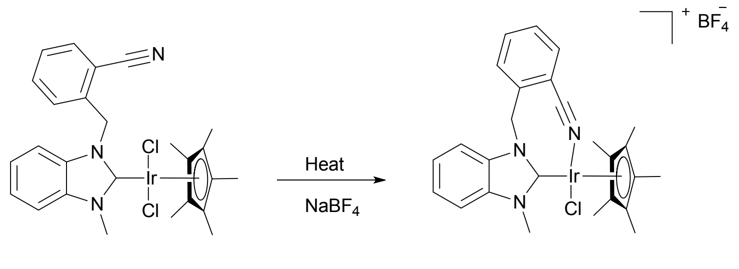

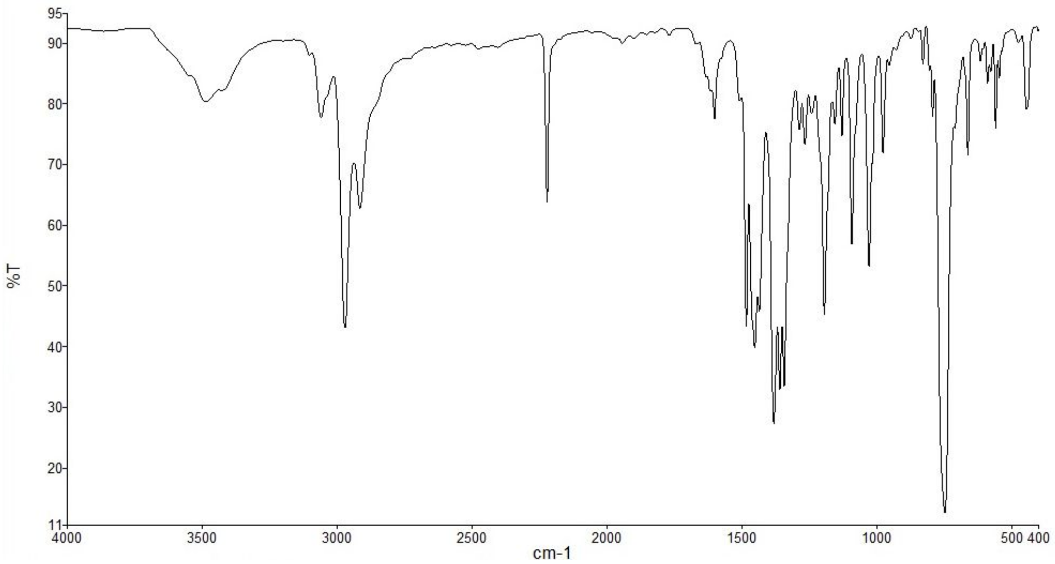

Figure S43. IR spectrum of **2b**.

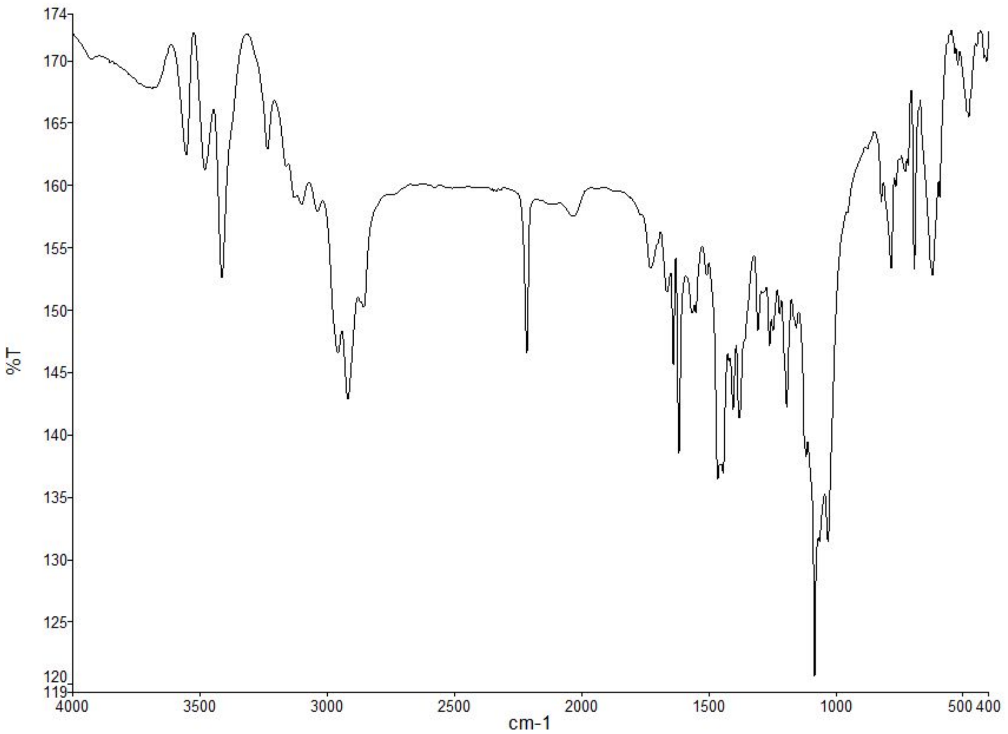

Figure S44. IR spectrum of **2b'**.
